# *Trypanosoma cruzi* transcriptome during axenic epimastigote growth curve

**DOI:** 10.1590/0074-02760170404

**Published:** 2018-04-03

**Authors:** Cyndia Mara Bezerra dos Santos, Adriana Ludwig, Rafael Luis Kessler, Rita de Cássia Pontello Rampazzo, Alexandre Haruo Inoue, Marco Aurélio Krieger, Daniela Parada Pavoni, Christian Macagnan Probst

**Affiliations:** 1 Universidade Federal do Paraná, Centro Politécnico, Programa de Pós-Graduação em Biologia Celular e Molecular, Curitiba, PR, Brasil; 2 Fundação Oswaldo Cruz-Fiocruz, Instituto Carlos Chagas, Curitiba, PR, Brasil; 3 Instituto de Biologia Molecular do Paraná, Curitiba, PR, Brasil

**Keywords:** Chagas disease, growth curve, transcriptomics, RNA-Seq, polysomes, RNA granules

## Abstract

**BACKGROUND:**

*Trypanosoma cruzi* is an important protozoan parasite and the causative agent of Chagas disease. A critical step in understanding *T. cruzi* biology is the study of cellular and molecular features exhibited during its growth curve.

**OBJECTIVES:**

We aimed to acquire a global view of the gene expression profile of *T. cruzi* during epimastigote growth.

**METHODS:**

RNA-Seq analysis of total and polysomal/granular RNA fractions was performed along the 10 days *T. cruzi* epimastigote growth curve *in vitro*, in addition to cell viability and cell cycle analyses. We also analysed the polysome profile and investigated the presence of granular RNA by FISH and western blotting.

**FINDINGS:**

We identified 1082 differentially expressed genes (DEGs), of which 220 were modulated in both fractions. According to the modulation pattern, DEGs were grouped into 12 clusters and showed enrichment of important gene ontology (GO) terms. Moreover, we showed that by the sixth day of the growth curve, polysomal content declined greatly and the RNA granules content appeared to increase, suggesting that a portion of mRNAs isolated from the sucrose gradient during late growth stages was associated with RNA granules and not only polyribosomes. Furthermore, we discuss several modulated genes possibly involved in *T. cruzi* growth, mainly during the stationary phase, such as genes related to cell cycle, pathogenesis, metabolic processes and RNA-binding proteins.

There is a substantial interest in understanding the cell biology of *Trypanosoma cruzi*, a protozoan parasite and the causative agent of Chagas disease, an endemic illness affecting 6-7 million people worldwide (mostly in Latin America), according to a 2016 estimate by the World Health Organization. *T. cruzi* belongs to the Trypanosomatidae family, which encompasses other human pathogens, such as *T. brucei* (responsible for African trypanosomiasis) and *Leishmania* spp. (responsible for leishmaniasis).

*T. cruzi* exhibits at least four stages in its complex life cycle that involves altering between two distinct hosts: insect vectors, where epimastigotes and metacyclic trypomastigotes are found, and mammalian hosts, where amastigotes and blood trypomastigotes are found. The epimastigote form is naturally found in the gut of infected insect vectors and can be maintained in the laboratory by cultivation in a rich medium, which is refreshed when the culture reaches high cell densities (Camargo 1964). Parasites multiply exponentially until they reach a stationary phase, when they respond to stresses, such as nutrient depletion, by ceasing growth and entering a non-proliferating quiescent state (Tyler and Engman 2000, Hernández et al. 2012). Moreover, a small proportion of cells in the stationary phase may undergo metacyclogenesis, giving rise to metacyclic trypomastigotes (Camargo 1964, Contreras et al. 1985). Several cellular changes occur during the growth of *T. cruzi* while transitioning from the exponential to stationary phase, such as elongation of both cell body and flagellum (Tyler and Engman 2000), reduction in nucleolus areas and its eventual disassembly, and a decrease in transcription and translation rates (Nepomuceno-Mejía et al. 2010, Hernández et al. 2012). Thus, the study of epimastigote growth in culture can help unveil several functional aspects of *T. cruzi*, from cell biology to gene expression regulation.

Trypanosomatids have a singular gene expression control mechanism (Clayton and Shapira 2007). These parasites transcribe long polycistronic arrays containing dozens of genes with no functional relationship among them. Next, polycistronic RNA is processed by concerted trans-splicing and polyadenylation reactions to produce mature mRNAs that contain the same 39-nucleotide species-specific spliced leader (SL), or mini-exon, at the 5’-end (Martínez-Calvillo et al. 2010). With the exception of the SL precursor gene, no RNA polymerase II promoter was detected in the upstream regions of polycistronic gene units in trypanosomatid genomes (El-sayed et al. 2005, Martínez-Calvillo et al. 2010). Interestingly, genes of the same polycistronic unit may show large differences in expression levels. It is believed that polycistrons are transcribed and processed constitutively and that gene expression is mainly regulated post-transcriptionally by selective transport to the cytoplasm, mRNA stability, and polysomal mobilisation (Clayton and Shapira 2007, Martínez-Calvillo et al. 2010, Pastro et al. 2017).

In the present work, we performed an RNA-Seq transcriptome analysis of the *T. cruzi* epimastigote life cycle stage *in vitro*, comparing total RNA and polysomal/granular (P/G) RNA during the entire growth curve. Our data provided insights into how epimastigotes regulate gene expression during transition from the exponential to stationary phase. Polysomal profile analysis showed a marked decrease in polysome formation during the stationary phase, whereas RNA granule content appeared to increase. Clustering and gene ontology (GO) analysis of RNA deep sequencing data of each RNA fraction provided insights into exploring and discussing the possible mechanisms that underlie epimastigote life cycle events during the axenic growth curve.

## MATERIALS AND METHODS

*T. cruzi maintenance and growth curve samples* - Epimastigotes of *T. cruzi* Dm28c were maintained in axenic conditions at 28ºC in liver infusion tryptose (LIT) medium supplemented with 10% heat-inactivated foetal bovine serum (Camargo 1964), and in logarithmic growth by continuous subculturing of 10^6^ cells/mL every three days. For RNA-Seq analysis, epimastigote culture samples were evaluated for 10 consecutive days in biological triplicates. For each replicate, the same original epimastigote culture was used to prepare 30 250-mL flasks with 100 mL of LIT containing 10^6^ cells/mL at the start of the experiment (day 0), to control for biological variation. Three culture flasks were used to determine cell density and growth curve daily, using an automatic counter (Z2Coulter®; Beckman Coulter). Based on this cell counting technique, a sufficient number of flasks for obtaining 10^8^ and 10^9^ cells were used daily for the extraction of total and P/G RNA, respectively. Differential counting analysis was performed by light microscopy using a Neubauer chamber to calculate the percentage of metacyclic trypomastigotes found in the culture. Total cell counts were within the same order of magnitude for both cell counting methods.

*Fluorescence*-*activated cell sorting fluorescence-activated cell sorting (FACS) analysis* - Cell cycle and cell viability analyses were performed using a FACS flow cytometer at the flow cytometry facility RPT08L of the Carlos Chagas Institute, Fiocruz Paraná, Brazil. For cell cycle assays, 2 × 10^6^ cells were harvested and fixed with 70% methanol at day 1, 3, 6, and 9 of the growth curve. After this step, cells were harvested again, washed in phosphate-buffered saline (PBS), and resuspended in 500 µL of a DNA staining solution containing 1.7 mM Tris-HCl, 15 µg/mL propidium iodide (PI), 0.05% NP-40 detergent, 5 mM NaCl, and 5 µg/mL RNase A. All experiments were performed at least in duplicate. A total of 20,000 events were acquired, and data analysis was also performed using FlowJo software (Treestar software). PE-Width × PE-Area gated cells were used to exclude debris and doublets during data analysis. The Dean-Jett-Fox model was used to estimate the percentage of cells in G0/G1, S, and G2/M phases of the cell cycle. For cell viability, 2 × 10^6^ cells were washed in PBS and incubated for 10 min at 28ºC in 5 µg/mL PI in PBS, and analysed by flow cytometry. The experiments were performed at least in triplicate during day 1-10 of the growth curve.

*Polyribosome isolation and analysis* - About 10^9^ cells were incubated with 100 µg/mL cycloheximide added directly into the culture medium and incubated at 28ºC for 10 min. After centrifugation at 7000 × *g* for 5 min, the cells were washed three times with NKM buffer (140 mM NaCl, 5 mM KCl, and 1.5 mM MgCl_2_) supplemented with 10 mM HEPES, 10 µg/mL cycloheximide, 10 µg/mL heparin, and 5 mM beta-mercaptoethanol. Pelleted cells were transferred to a new tube containing 15 mL hypotonic lysis buffer (10 mM Tris-HCl pH 7.5, 10 mM MgCl_2_, 10 mM NaCl, and 5 mM beta-mercaptoethanol) and briefly vortexed. Next, 0.25 M sucrose and 1% NP-40 were added to stabilise the lysate. The lysate was then centrifuged at 4ºC for 30 min at 10,000 × *g*. The cleared supernatant was collected and carefully layered onto a 2 M sucrose cushion and ultra-centrifuged at 4ºC for 2 h at 39,000 × *g* in a Beckman SW40 rotor. The polysomal fraction was recovered from the final pellet.

Polysome profile analysis was performed according to Gradia et al. (2009), with modifications to the TMK buffer (10 mM Tris-HCl pH 7.4, 10 mM MgCl_2_, and 300 mM KCl) and the linear 15-55% (w/v) sucrose gradient. The sucrose gradient was collected at a rate of 1 mL per minute and analysed spectrophotometrically (254 nm) using an ISCO Foxy JR system. Aliquots of each collected fraction (30 µL) were analysed by SDS-PAGE followed by western blotting, with antibodies against *T. cruzi* ribosomal protein S7 (1:500). After washing, the membrane was incubated with IRDye® 680RD secondary antibodies (LI-COR) for 1 h at room temperature. The membrane was washed three times with PBS containing 0.1% Tween-20, and antibody binding was detected using the Odyssey Infrared Imaging System (LI-COR).

*Microscopy analysis* - Parasites were harvested by centrifugation, washed in PBS (pH 7.4), fixed by incubation in 4% paraformaldehyde, and adhered to poly-L-lysine-coated slides for 10 min. For immunofluorescence assays, slides were washed in PBS and parasites were permeabilised by incubation with 0.01% Triton X-100 diluted in PBS for 2 min. Next, cells were blocked with blocking buffer containing 4% BSA diluted in PBS for 1 h at 37ºC. For detection of TcDhh1 granules, mouse anti-TcDhh1 antibody (1:500; kindly provided by Fabíola B Holetz and Jimena Ferreira da Costa), and Alexa Fluor 488-conjugated goat anti-mouse IgG secondary antibody (1:600; Invitrogen) were used. A test without addition of primary antibodies was used as a negative control.

For localisation of mRNA by fluorescent *in situ* hybridisation (FISH), slides were washed in PBS and parasites were permeabilised by incubation with 0.2 M HCl diluted in PBS for 10 min. Next, cells were incubated with pre-hybridisation buffer containing 35% formamide, 0.02% BSA in 2 × SSC buffer, 25 μg/mL tRNA, 1 mg/mL salmon sperm DNA (Sigma-Aldrich), and 40 U/mL RNaseOUT (Invitrogen) for 30 min at 37ºC. For detection of poly (A)^+^ RNA, digoxigenin-conjugated oligo (dT) probes (6 ng/μL) were diluted in pre-hybridisation buffer and denatured by heating at 65ºC for 3 min. As control, 100 μg/mL RNase A was added to the pre-hybridisation buffer before probe hybridisation. Hybridisation was performed for 16 h at 37ºC. Probe binding was detected by indirect immunofluorescence analysis with mouse monoclonal anti-digoxigenin antibody (1:300; Sigma-Aldrich) and Alexa Fluor 488-conjugated goat anti-mouse IgG secondary antibody (1:600).

DNA was stained by incubating with 5 µg/mL DAPI for 15 minutes. Slides were analysed with an inverted microscope (Leica DMI6000B) using the deconvolution software Leica AF6000 at the microscope facility RPT07C PDTIS of the Carlos Chagas Institute, Fiocruz Paraná. The mRNA distribution pattern and the TcDhh1 localisation were quantified by counting the number of granules observed in 100 cells on day 3, 6, and 9 using ImageJ software (version 1.51q; NIH, MD, USA). Cell number was calculated based on automatic counting using DAPI nuclear labelling. The number of granules (TcDhh1 or mRNA granules) was estimated by intensity and size of pixels defined by the fluorescence signal of 8-bit converted images.

*RNA isolation* - Total and P/G RNA were isolated using the RNeasy mini kit (Qiagen) and treated with RNase free DNase I (Qiagen). RNA samples were quantified by absorbance at 260 nm using the Nanodrop ND-1000 (Thermo Fisher Scientific). Subsequently, RNA integrity and quality was analysed using the Agilent 2100 bioanalyzer and the RNA 6000 Pico Kit (Agilent). mRNA was selected by oligo-dT chromatography using the PolyATtract® mRNA Isolation Kit (Promega).

*RNA-Seq* - Samples were prepared for RNA sequencing using the SOLiD^TM^ Total RNA-Seq Kit (#4445374; Life Technologies) according to the manufacturer’s instructions. Briefly, RNA samples were fragmented using RNase III and cleaned up with the Invitrogen RiboMinus™ Concentration Module. Strand-specific adaptors were hybridised and ligated to both extremities of RNA in an overnight reaction, followed by first strand cDNA synthesis with reverse transcriptase. cDNA was purified using the MinElute® PCR Purification Kit (Qiagen) and size-selected using a Novex TBE-Urea 6% gel (#EC6865BOX). On-gel cDNA was amplified by polymerase chain reaction (PCR) using SOLiD^TM^ RNA barcoding kit primers (#4427046 and #4453189; Life Technologies) and purified with the Invitrogen PureLink® PCR Micro Kit. Amplified DNA was quantified with the Qubit^®^ 2.0 Fluorometer and Qubit® dsDNA HS Assay Kit. Equal mass of each sample, containing specific barcodes, was pooled together and the mixtures were used as DNA templates for emulsion PCR using Applied Biosystems SOLiD® EZ Bead™ E80 system (#4453095). Barcoded libraries were sequenced on a SOLiDTM 4 system using multiplex fragment sequencing protocol (SOLiD ™ 4 System Library Preparation Guide, applied biosystems).

*RNA-Seq data processing* - Raw single reads of 50 nucleotides were quality-filtered using the standard SOLiD^TM^ WT Analysis Pipeline tool. Reads were mapped to *T. cruzi* CL Brener genome (El-Sayed et al. 2005), using SHRIMP2 (David et al. 2011). To exclude redundancy in *T. cruzi* gene annotation and read counts, clusters of orthologous genes were determined based on nucleotide sequence similarity and named as supra genes (SGs). SGs are groups of genes containing high similarity nucleotide sequences, which eliminate redundancy in the transcriptome profile. SG read counts were obtained by summing the number of reads aligned to each coding sequence (CDS) of all SG members using Perl programming language script. SG read counts were used to analyse differential expression. Low-expressed SGs were not considered in the analysis if counts per million was less than 1 for at least two samples. Read counts were normalised and analysed for differential expression using edgerR package (Robinson et al. 2010), considering different levels of statistical significance and false discovery rates (FDR) with a maximum value of 10% and a second filter log_2-_fold-change ≥ 1.0 (log_2_ FC). Non-linear regression data analysis was performed using R software to obtain an average measure of expression.

*Clustering of gene expression patterns* - Expression levels (normalised RNA-Seq counts) of differentially expressed genes (DEGs; FDR < 0.01) were normalised between samples using the Z value [(counts - mean counts)/counts standard deviation] to place all genes within the same scale with a mean of zero and standard deviation of one. The transformed profiles were then clustered with the fuzzy c-means (FCM) clustering algorithm (Futschik and Carlisle 2005) based on the open-source programming language R Bioconductor (bioconductor.org). The main parameters (c, number of clusters and m, fuzzification parameter) were derived by the iterative refinement procedure described by Futschik and Carlisle (2005). The final clustering of gene was performed with c = 12 and m = 1.13. Only those genes with a membership value higher than 0.5 were considered for GO analysis.

*GO analysis* - Improved annotation of identified proteins and GO classification was achieved with Blast2GO software (Conesa et al. 2005) using default parameters. Over representation of GO terms for specific gene sets was evaluated using Fisher’s exact test with an FDR of 0.01 and by seeking most specific GO terms.

## RESULTS

**Fig. 1:**
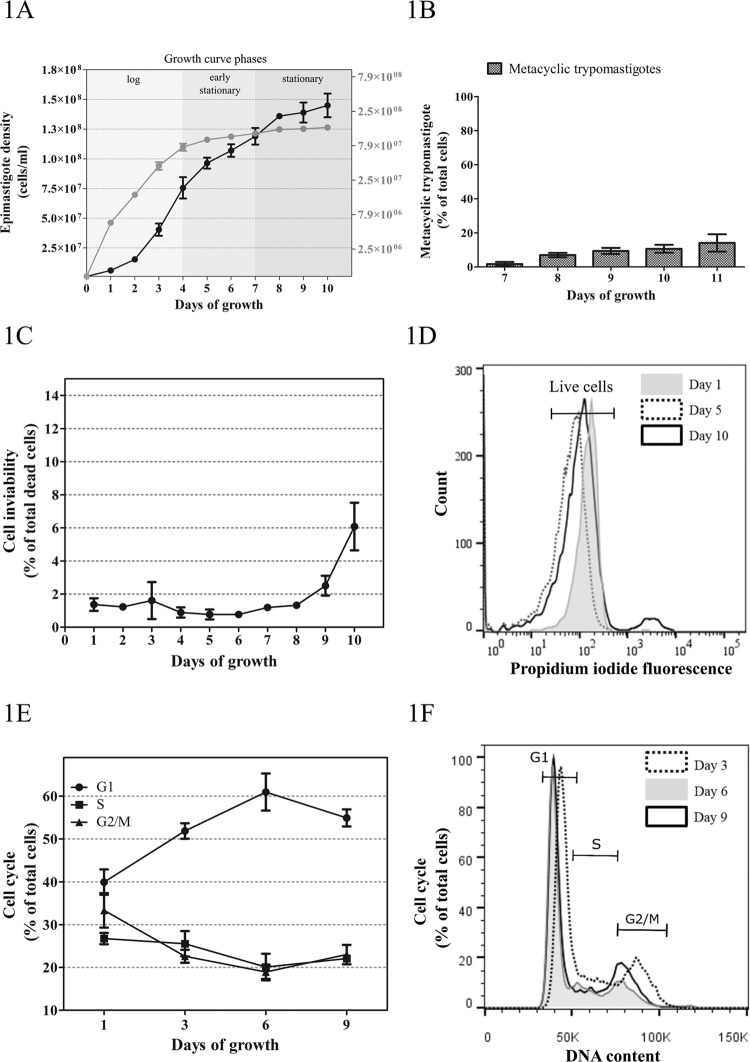
: analysis of axenic epimastigote growth. (A) Parasitic growth in culture was measured daily over a 10-day period and cell densities were plotted against time showing the growth curve. Left y-axis, linear scale; right y-axis, logarithmic scale. (B) Percentage of metacyclic trypomastigotes during epimastigote culture. (C) Cell viability was measured daily by propidium iodide (PI) staining over a 10-day period and plotted against time. (D) Cell viability profiles of epimastigote cells with PI fluorescence (indicating live vs. dead cells) at day 1, 5, and 10. (E) Cell cycle distribution was assessed at day 1, 3, 6, and 9 of epimastigote growth and plotted against time. (F) Flow cytometry profiles of epimastigote cells with PI fluorescence (indicating DNA content) at day 3, 6, and 9. Values are expressed as the mean ± SD of three biological replicates.

*T. cruzi epimastigote (Dm28c) growth curve patterns* - Epimastigote growth curve was initiated at 1 × 10^6^ cell/mL and followed for 10 consecutive days (Fig. 1A). We observed a typical growth curve with distinct phases. An exponential or log phase from day 1 to 4 reaching 7.56 ± 0.89 × 10^7^ cells/mL, an early stationary phase from day 4 to 7 where cells continue dividing with a clearly reduced growth rate reaching 11.9 ± 0.70 × 10^7^ cells/mL, and a stationary phase from day 7 to 10 reaching relatively stable cell density. There was a small percentage of metacyclic trypomastigotes first detected by day 7 (1.73 ± 1.24%), which gradually increased up to 10.66 ± 2.35% by day 10 (Fig. 1B).

By microscopic examination, we verified normal epimastigote morphology and flagellar motility for most of the growth curve. However, at the stationary phase, the parasites exhibited body and flagellar elongation, and a high number of cells without motility (45% by day 9; data not shown). Next, we analysed cell viability during the entire growth curve. The results showed that the percentage of dead parasites was stable until day 8 with an average of 1.14 ± 0.30%, reaching 6.08 ± 1.44% by day 10 (Fig. 1C-D). Thus, we assumed that the majority of immobile cells were still alive.

We also analysed the cell cycle on day 1, 3, 6, and 9 by flow cytometry (Fig. 1E-F). The proportion of parasites in G1 phase increased during the growth curve from 39.93 ± 2.94% at day 3 to 60.95% ± 4.34% by day 6, after which it remained relatively stable. Accordingly, cells in S and G2/M phases were present in smaller fractions of 26.73% (± 1.30%) and 33.33% (± 4.03%) at day 1, respectively. These fractions decreased during the growth curve, reaching 22.06% (± 0.48%) and 23.00% (± 2.24%) for S and G2/M phases by day 9, respectively.

**Fig. 2:**
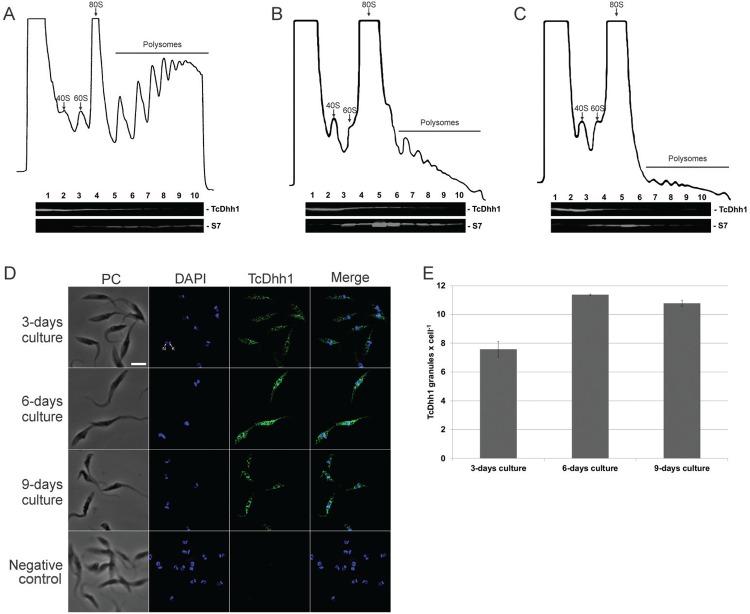
: polysomes and mRNA granule analysis. (A-C) Sedimentation profile on a sucrose density gradient (15-55%) of day (A) 3, (B) 6, and (C) 9 of the *Trypanosoma cruzi* growth curve. Fractions (1-10) were analysed by western blotting using antibodies against TcDhh1 and ribosomal protein S7. The 40S and 60S ribosomal subunits as well as the 80S ribosome monomer and polysomes are indicated in (A–C). (D) Cellular localisation of TcDhh1. Detection of TcDhh1 by indirect immunofluorescence. PC: phase contrast; DAPI: DNA stained with DAPI; TcDhh1: endogenous TcDhh1; MERGE: merged images of DAPI and TcDhh1 immunofluorescence; N: nucleus; K: kinetoplast. Scale bar = 5 μm. Negative control was assayed without primary antibody. (E) Histogram of TcDhh1 granule counts per cell.

*Polysome fraction decreased while the number of mRNA granules increased during stationary phase* - In actively growing cells, the rate of translation appears to remain constant. As a result, multiple ribosomes simultaneously engaged in translation are present on single mRNA molecules. We analysed the polysome profile of epimastigotes from day 3, 6, and 9 to evaluate when translation is affected in the *T. cruzi* growth curve. Polysome formation was widely disrupted on day 6 and 9 demonstrating decreased translation starting at the early stationary phase (Fig. 2A-C).

**Fig. 3:**
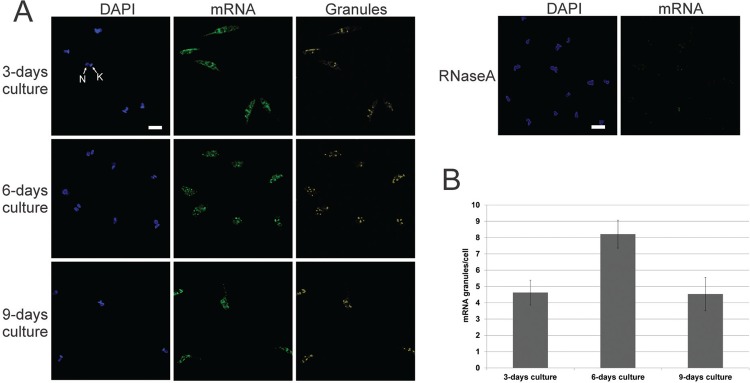
: localisation of mRNA. (A) Fluorescent *in situ* hybridisation assay using specific oligo (dT) probe. As the control, RNase A was incubated with the parasites before probe hybridisation. DAPI: DNA stained with DAPI; mRNA: mRNA detection; granules: labelling of pixels with high intensity fluorescence signals; N: nucleus; K: kinetoplast. Scale bar = 5 µm. (B) Histogram of mRNA granule counts per cell.

However, since polysome content decreased in the stationary phase, it is possible that mRNA isolated from the sucrose cushion is from ribonucleoprotein complexes rather than polysomal RNA. This notion was corroborated by FISH analysis (Fig. 3A); therefore, we termed the sucrose cushion fraction P/G RNA. At day 3, parasites showed a cytoplasmic mRNA localisation enriched around the nucleus and an average of 4.6 granules/cell, while parasites at day 6 showed a higher number of RNA granules (average of 8.2 granules/cell). By day 9, parasites showed an average of 4.53 granules/cell (Fig. 3B).

To confirm the increased number of granules, we performed western blot analysis using an antibody against the granule marker TcDhh1, an ATP-dependent DEAD-box RNA helicase present in *T. cruzi* cytoplasmic mRNA granules that resemble P-bodies and increase number during nutritional stress (Holetz et al. 2007). The sedimentation profile of TcDhh1 on a sucrose density gradient was similar in all tested conditions (Fig. 2A-C), indicating that TcDhh1 is present in polysome-independent complexes. TcDHH1-containing granules were quantified and the results indicated an increased number of granules in parasites by day 6 (11.3 granules/cell) and 9 (10.7 granules/cell; Fig. 2D-E).

**Fig. 4:**
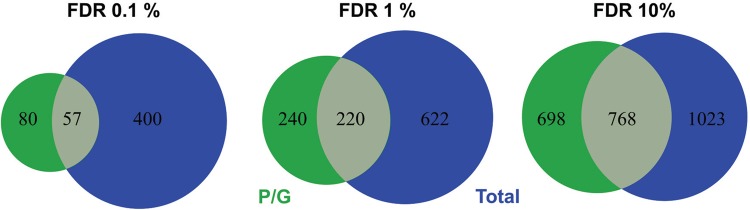
: comparison between total (blue) and polysomal/granular (green) Euler diagram of RNA-Seq differentially expressed genes (DEGs) identified during the growth curve. DEGs with 0.1, 1.0, and 10% false discovery rates (FDRs) were considered.

*RNA-Seq analysis and identification of differentially expressed genes (DEGs)* - We performed RNA-Seq analyses to evaluate spatiotemporal gene expression profiles along the epimastigote growth curve. For each of the 10 days, two libraries were constructed of total RNA and P/G RNA. The total number of reads generated was 455 million for the total RNA library and 412 million for the P/G RNA library. The average number of reads per sample was 15.7 million and 19.6 million for total and P/G RNA, respectively [Supplementary data (Tables I-II)]. Multiple sample test analysis identified a total of 1082 DEGs (FDR < 1%) during the growth wherein 842 and 460 DEGs were modulated in the total RNA and P/G RNA fractions, respectively. Approximately, 20% of DEGs (220) were modulated in both RNA fractions, 622 (57.48%) were modulated only in the total RNA fraction, and 240 (22.18%) only in the P/G RNA fraction. Fig. 4 shows the number and overlapping of DEGs between analysed RNA fractions at distinct FDR thresholds. Supplementary data (Tables III-IV) lists the DEGs found with FDR < 10% for total and P/G RNA fractions, respectively.

For FDR complete statistic and RPKM data of all genes: https://drive.google.com/open?id=1KniMMxvVZkAMkR8xbNW92GiupsKPYnd9.

**Fig. 5:**
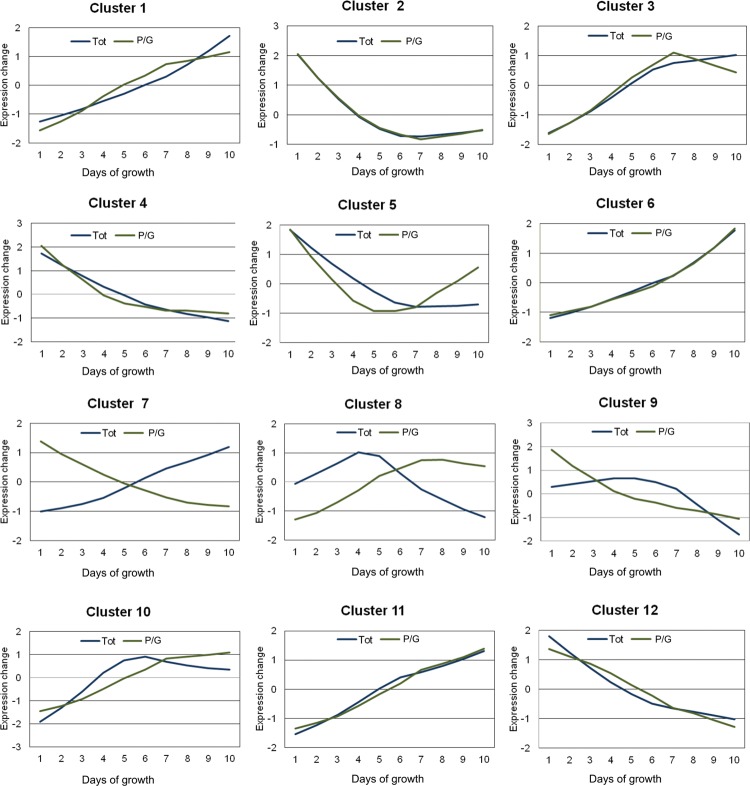
: temporal patterns of gene expression during the *Trypanosoma cruzi* growth curve identified by Fuzzy c-means clustering. Only centroids of the 12 identified clusters are shown.

*Correlation of gene expression over time* - To understand the dynamics of gene expression between total and P/G RNA fractions during the growth curve, we clustered DEGs according to their normalised expression patterns using Fuzzy c-means clustering. The optimal number of clusters was iteratively determined to be 12. This analysis considered all DEGs in total and P/G RNA fractions detected by multiple sample tests. For DEGs that are specific for one fraction, their respective expression data from the other RNA fraction was included. A total of 1801 genes were successfully determined to the most probable expression profile cluster (membership value > 0.5) and Fig. 5 shows the centroid expression pattern of the 12 clusters discussed above. Supplementary data (Table V) lists the genes in each cluster.

Clustering determined that 995 (92%) genes showed similar modulation patterns in both RNA fractions (clusters 1, 2, 3, 4, 6, 10, 11, and 12). Cluster 1 (142 genes), 6 (129 genes), 10 (34 genes), and 11 (211 genes) exhibited a constant increase in RNA expression in both RNA fractions, while cluster 2 (133 genes), 4 (171 genes), and 12 (87 genes) showed a decreasing expression profile. Cluster 3 (88 genes) displayed increasing RNA expression until day 7, after which total RNA levels remained constant while P/G RNA levels decreased. Cluster 7 (16 genes) showed an interesting inverse pattern between total and P/G RNA fractions, with increasing and decreasing expression patterns, respectively. Cluster 5 (38 genes) showed a reduction in gene expression for both fractions until day 7 where expression remained almost stable in the total RNA fraction and increased in the P/G RNA fraction. Cluster 8 (13 genes) exhibited similar modulation of both RNA fractions with an increase in gene expression followed by a decrease; however, this cluster switched patterns by day 4 for the total RNA fraction and by day 7 for the P/G RNA fraction. Cluster 9 (19 genes) RNA levels of the total fraction showed reduced expression after day 7, while the P/G RNA fraction showed a constant decrease along the growth curve. Cluster 10 (34 genes) showed an increase in RNA expression until day 6 for the total fraction and day 7 for the P/G fraction, after which expression declined in the total fraction and remained relatively stable in the P/G fraction. Interestingly, there was a slight change in RNA expression patterns of several clusters at day 7, mainly in the P/G RNA fraction (clusters 1, 2, 3, 5, 8, 9, and 10).

**Fig. 6:**
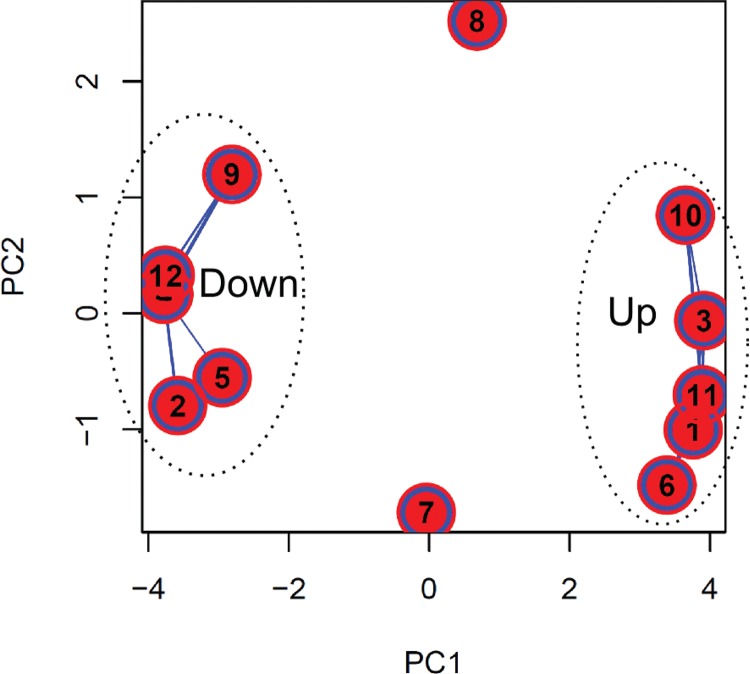
: principal component analysis (PCA) showing the distance between each cluster of differentially expressed genes (DEGs). Line width connecting the points represents the number of genes shared between groups during fuzzy clustering iterations. Dotted ellipses represent clusters with similar expression patterns that were grouped for gene ontology (GO) analysis (see text). This graph was plotted using the *overlap.plot* function of the fuzzy-c-means algorithm.

*Functional enrichment analysis* - To improve our understanding of gene expression clusters and provide insights into functional properties, cluster compositions were used for GO enrichment analysis by Blast2GO software. The large proportion of hypothetical genes in *T. cruzi* without GO annotation makes the identification of statistically enriched GO terms difficult. To optimise GO analysis and the probability of finding enriched terms, we grouped clusters with similar expression patterns (Fig. 6). Thus, clusters that presented a similar modulation pattern were grouped, while singular ones remained isolated. The first group was composed of clusters 2, 4, 5, 9, and 12 as they have a downregulated pattern of gene expression. The second group was composed of clusters 1, 3, 6, 10, and 11 as they have an upregulated pattern of gene expression for both RNA fractions. Finally, we also analysed clusters 7 and 8 that exhibited more distinct modulation patterns.

GO terms were assigned for 565 genes. Unfortunately, it was not possible to obtain significantly enriched GO terms for clusters 7 and 8, possibly because they are composed of a few genes that mainly encode hypothetical proteins.

**Fig. 7:**
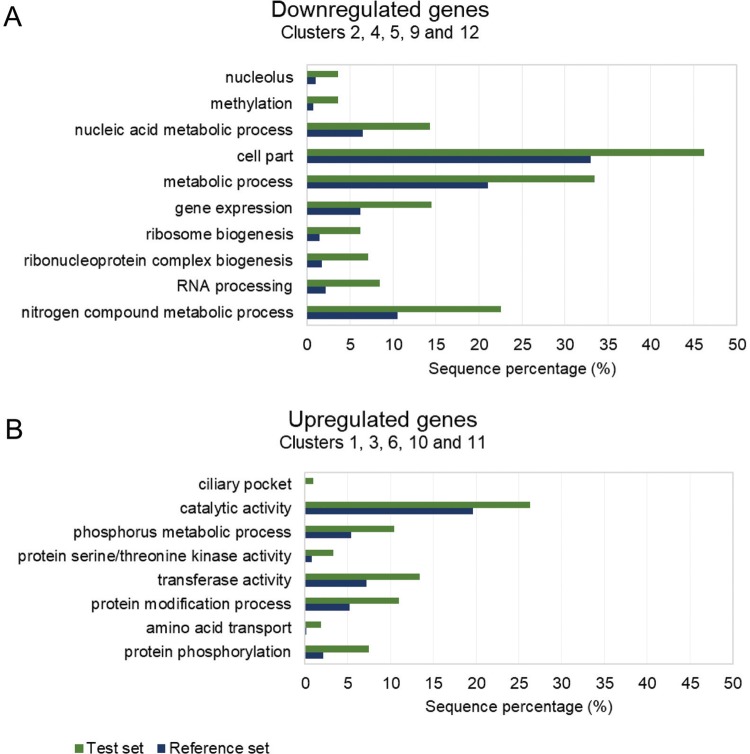
: enriched categories of gene ontology (GO) terms for different clusters. (A) Downregulated genes. (B) Upregulated genes.

Some functional terms found for the downregulated group (Fig. 7A) were nucleolus (GO: 0005730), methylation (GO: 0032259), nucleic acid metabolic process (GO: 0090304), cell part (GO: 0044464) including several mitochondrial-related genes, metabolic process (GO: 0008152), gene expression (GO: 0010467) including proteins mainly involved in the translation process, ribosome biogenesis (GO: 0042254), ribonucleoprotein complex biogenesis (GO: 0022613), RNA processing (GO: 0006396), and nitrogen compound metabolic process (GO: 0006807).

For the upregulated group, several GO terms were also identified (Fig. 7B) with the most enriched terms being ciliary pocket (GO: 0020016), catalytic activity (GO: 0003824), transferase activity (GO: 0016740), and amino acid transport (GO: 0006865). Another set of genes highly represented in these clusters are those encoding kinase proteins related to phosphorus metabolic process (GO: 0006793), protein serine/threonine kinase activity (GO: 0004674), protein modification process (GO: 0036211), and protein phosphorylation (GO: 0006468); additionally, several genes from this group are members of the trans-sialidase superfamily involved in pathogenesis of the parasite.

## DISCUSSION

*Evaluation of the T. cruzi growth curve* - We explored some cellular and biological aspects of the *T. cruzi* epimastigote growth curve over 10 consecutive days *in vitro*. Epimastigotes replicate by binary fission and show a typical *in vitro* growth curve with two major distinct phases, an exponential, and a stationary phase. The exponential phase is characterised by a constant exponential growth rate where population density increases. The stationary phase occurs when cells arrest their growth, mainly in response to starvation (Werner-Washburne et al. 1993). We also observed the existence of a transitory phase between the exponential and stationary phases (day 5-7), with a significant decrease in growth rate before virtually ceasing. These observations are expected because epimastigote growth appears to be controlled by nutritional depletion and cell density (Camargo 1964, Tyler and Engman 2000). Interestingly, clustering analyses showed many clusters with a slight change in RNA expression levels by the 7th day of the growth curve, coinciding with the beginning of the stationary phase. These changes could be explained, in part, by global RNA synthesis repression that is observed in non-proliferative stages of the *T. cruzi* life cycle (Elias et al. 2001). However, these changes may also be due to gene-specific modulations.

We observed a high number of parasites without motility at the stationary phase, yet cell viability analysis indicated that most of these cells were still alive. This observation corroborates the hypothesis that cells at the stationary phase reach a quiescent stage. For some microorganisms, the stationary phase is mainly represented by cells arrested in the G0 phase of the cell cycle (Werner-Washburne et al. 1993). In *T. cruzi*, we observed an increase in cell percentage in G0/G1 phase, although approximately 40% appeared to be dividing or arrested in S or G2/M. Cell cycle regulation is still poorly understood in trypanosomatids, but several genes whose products appear to be involved in this process were identified (Hammarton et al. 2007, Potenza et al. 2012). Some of these genes were found to be modulated in our data.

Specific molecules that regulate transition through different phases of the cell cycle, such as cyclins and Cdc2-related kinases (CRKs) have already been studied in *T. brucei*. Here, we investigated the mRNA expression patterns of their homologous genes in *T. cruzi*. Five of 10 cyclins were found to be upregulated during the growth curve [TcCLB.506791.10, CYC2-like cyclin in cluster 11; TcCLB.508207.250, CYC8 in cluster 3; TcCLB.507677.150, CYC5 in cluster 1; TcCLB.503885.100, CYC10, not clustered; TcCLB.503551.20, CYC11 (FDR < 0.1)]. RNA interference (RNAi) studies in *T. brucei* showed that CYC2 and CYC4 play major and minor roles in promoting G1/S transition, respectively (Li 2012). Moreover, four of 11 CRKs also showed modulation patterns (TcCLB.503617.10, CRK2; TcCLB.509099.150, CRK9; TcCLB.510329.70, CRK10; TcCLB.507023.180, CRK11 in clusters 6, 7, 11, and 3, respectively). CRK2 appears to be involved in G1/S transition and seems to interact with CYC2 (Gourguechon et al. 2007).

Downregulation of selected genes via RNAi identified several *T. brucei* genes as essential to cytokinesis (Hammarton et al. 2007). Interestingly, we found that some of the homologous genes in *T. cruzi* showed a downregulated pattern during the stationary phase. These genes mainly corresponded to those genes encoding flagellar related proteins and centrins [TcCLB.511729.60, MORN repeat-containing protein 1 in cluster 12; TcCLB.509171.100, hydin, not clustered; TcCLB.506887.20, parkin co-regulated protein (FDR < 0.1); TcCLB.508323.60, centrin 1 (FDR < 0.1); TcCLB.510763.100, trypanin (FDR < 0.1); TcCLB.506401.90, centrin 2, not clustered].

As expected, for asynchronous experiments, we did not find genes with a cyclic pattern of expression, evidenced by the cluster analysis performed in this study. Archer et al. (2011) performed a cell cycle transcriptome study in *T. brucei* under highly synchronous cell culture conditions and did not observe a cyclic pattern either, suggesting that the lack of synchronisation in our experiment did not result in the absence of a cyclic pattern of gene modulation.

*mRNA dynamics and their association with polyribosomes and granules during the growth curve* - Gene expression control in trypanosomatids occurs post-transcriptionally. A recent transcriptome study showed that nucleus and cytoplasmic compartmentalisation act as a control step in stage-specific gene regulation in *T. cruzi* (Pastro et al. 2017). Mature mRNAs exported to the cytoplasm are exposed to different modes of regulation that ultimately lead to mRNA translation, RNA degradation, and association with protein-RNA complexes (Clayton and Shapira 2007).

Like other eukaryotes, *T. cruzi* mRNA can be compartmentalised in ribonucleoprotein complexes called P-bodies or stress granules. The presence of mRNA granules in *T. cruzi* was observed under nutritional stress (Cassola et al. 2007, Holetz et al. 2007). In our results, TcDhh1 was found in polysome-independent complexes, as previously demonstrated for parasites under nutritional stress (Holetz et al. 2007). It has been suggested that mRNAs can be protected in granules for further translation, as a coping strategy for periods of starvation (Cassola et al. 2007). In this context, the transcriptome analysis of a complete set of mRNAs (total fraction) versus a set of mRNAs linked to polysomes or other ribonucleic complexes (P/G RNA fraction) may provide insights into the mechanisms underlying gene expression regulation.

We observed that the rate of translation appears to diminish during the stationary phase, as parasite polysome formation was disrupted on day 6 and 9. This is in agreement with a previous study, which showed that after epimastigotes are exposed to nutritional stress, polysomes are disrupted and global translation initiation is drastically inhibited, accompanied by phosphorylation of eIF2 alpha (Tonelli et al. 2011). Moreover, the increased number of RNA-containing granules during early stationary phase (day 6) is consistent with the findings of other that cultured parasites in a nutritionally poor environment (Holetz et al. 2007). However, granule content based on FISH analysis by day 9 was similar to the content exhibited by exponential-growth parasites at day 3. This could be attributed, at least in part, to a global reduction in mRNA abundance and increasing cell death, which led to a decreased FISH-staining intensity. Furthermore, since P-bodies are related to mRNA-degradation (Parker and Sheth 2007), and TcDhh1 granules were represented more on day 6 and 9, it is likely that the granular content of mRNAs decreased by day 9 due to degradation. Even though the ISCO polysome profile analyses are possibly not sensitive enough for low levels of translation, our observations suggest that most mRNAs isolated from the sucrose cushion during the stationary phase may be associated with ribonucleoprotein granules instead of free polyribosomes.

*Biological processes and molecular functions implicated in the T. cruzi growth curve* - Analysis of RNA expression data revealed a very similar modulation pattern between total and P/G RNA fractions. This was not exactly unexpected, as the polysomal portion is included in the total fraction. One of our goals was to identify differences in RNA fraction profiles that could reveal regulation dynamics along the growth curve.

We were able to recognise two clusters (7 and 8) that exhibited distinct total and P/G RNA modulation patterns. Cluster 7 genes showed a modulation pattern suggesting a constant reduction in translation, because their expression decreased in the P/G RNA fraction in spite of increasing in the total RNA fraction. The genes present in this cluster are DNA polymerase epsilon subunit B, ribosomal protein L7/L12, ADG2, cdc2-related kinase 9, and several hypothetical proteins. Genes from cluster 8 also encode hypothetical proteins as well as dynein light and heavy chains, among others. These genes appear to have a delay in protein production relative to mRNA synthesis, as their P/G RNA fraction expression pattern was very similar to total RNA, but delayed by approximately three days.

GO enrichment analyses can help identify important genes and processes involved in major cellular and molecular changes that occur during epimastigote growth curve. Unfortunately, a great number of the modulated genes were annotated as hypothetical proteins due to the lack of information regarding their probable functions. Nevertheless, we were able to obtain significant information about *T. cruzi* growth curve, mainly in relation to the stationary phase.

Trans-sialidases are important pathogenesis factors that mediate transfer of sialic acids from the host glycoconjugates to the surface of the parasite. In our study, several upregulated genes belong to the trans-sialidases superfamily, corroborating the notion that stationary epimastigotes are a pre-adaptive stage for metacyclic trypomastigotes, since after metacyclogenesis, the parasites are ready for interaction with a new host (Hernández et al. 2012).

During the stationary phase, we observed parasite flagellar elongation that is in accordance with the findings of Tyler and Engman (2000). The modulation of some genes (such as the flagellum-related genes) may be implicated in this process. Additionally, the gene encoding “paraflagellar rod protein” (TcCLB.509099.30; FDR < 0.1), an important structural component of flagellum (Portman and Gull 2010), was upregulated until day 7 in the P/G RNA fraction, after which its expression remained constant. Another cellular consequence observed in *T. cruzi* during the stationary phase is the significant reduction in nucleoli area (Nepomuceno-Mejía et al. 2010). In accordance with this observation, we found a general downregulation of nucleolus-related genes.

Nutritional stress by glucose exhaustion is a possible reason for the elongation of cell body and flagellum, reduction in the area of nucleolus, and diminished transcription and translation in the epimastigote form (Tyler and Engman 2000, Nepomuceno-Mejía et al. 2010). It has been reported that glucose is rapidly consumed during the exponential phase, after which cells obtain energy by amino acid catabolism (Urbina 1994). Accordingly, we observed an enrichment of amino acid transport-related genes among the upregulated group.

During the stationary phase, we also observed an enrichment of genes associated with metabolic processes in the downregulated group, such as non-coding RNA, rRNA, macromolecules, steroids, organophosphates, purine nucleosides, and ribonucleosides, among other processes.

*RNA-binding proteins (RBPs)* - Gene regulation in kinetoplastids is partly controlled by RBPs, which are trans-acting factors that are involved in several processes from initial mRNA processing to mRNA decay (Clayton 2013). A high number of RBP candidate genes were identified in trypanosomatids, including proteins containing the Alba, Pumilio, zinc-finger, and RNA recognition motif (RRM) domains (de Gaudenzi et al. 2005, Clayton 2013). Several *T. cruzi* RBP genes were found modulated along the growth curve.

Some specific RBP genes, which were found to be modulated in our data have been better studied in *T. brucei* and appear to play a role in regulation of mRNA levels or translation (Clayton 2013). For example, we found that the genes encoding two Pumilio-domain proteins were downregulated during the growth curve [TcCLB.511715.100, Pumilio/PUF 7 (FDR < 0.05); TcCLB.508799.70, Pumilio/PUF10 in cluster 4]. *T. brucei* PUF7 and PUF10 are necessary for rRNA maturation in the nucleolus (Clayton 2013).

Some Alba-domain proteins (ALBA1, 2, 3, and 4) can interact with each other and are involved in the translation process (Clayton 2013). We found that the mRNA levels of two *T. cruzi* Alba genes were reduced [TcCLB.504089.60, ALBA1 (FDR < 0.05); TcCLB.504001.20, ALBA2 in cluster 4]. Interestingly, ALBA2 in *T. brucei* was found to be associated with polyribosomes via sucrose gradient analysis (Mani et al. 2011). In accordance with this observation, we also found ALBA2 mRNA in the polysomal fraction, mainly during exponential growth phase, with a decreasing polysomal association along the growth curve.

Of the RRM-containing proteins whose genes were upregulated, some were found to be noteworthy. RBP42 (TcCLB.485683.10; upregulated, cluster 6) that in *T. brucei* is partially associated with polysomes and is involved with many mRNA target sites related to energy metabolism. RBP6 (TcCLB.508153.680; cluster 3) and RBP10 (TcCLB.510507.50; cluster 11) have been implicated in the developmental progression of *T. brucei*. RBP6 promotes differentiation to epimastigote and trypomastigote metacyclic forms, whereas RBP10 possibly prevents the expression of procyclic form-specific regulatory proteins (Clayton 2013).

Homologues of three important *T. brucei* zinc-finger proteins were found to be modulated during the *T. cruzi* growth curve. Of these three proteins, ZFP1 (TcCLB.511511.3; cluster 3) is specific to the procyclic stage and plays an important role in the differentiation from bloodstream to procyclic form, ZC3H20 (TcCLB.506859.204, upregulated during exponential phase, not clustered) is required for the growth of procyclic forms, and ZC3H11 (TcCLB.504929.5; cluster 11) is a post-transcriptional regulator that binds several chaperone mRNAs for stabilisation after heat shock of procyclic forms (Clayton 2013).

Few *T. cruzi* RBPs are well characterised and the homology-based function assignment based on *T. brucei* is a challenge. However, target mRNAs of some *T. cruzi* RBPs were identified and many of those targets were found to be modulated along the growth curve (Supplementary data, *Expression pattern of RBPs partners in Trypanosoma cruzi growth curve*). TcZC3H39 protein is an important component of ribonucleoprotein complexes that become more pronounced under stress conditions (Alves et al. 2014). In stressed parasites, TcZC3H39 targets were mRNAs encoding ribosomal proteins, and a remarkable enrichment in mRNAs for the cytochrome *c* complex (COX) was observed (Alves et al. 2014). Consistent with the increase in ribonucleoprotein complexes in the stationary phase, we found that several mRNAs of several of TcZC3H39 mRNAs targets were upregulated during the last days of the curve when the parasites are under severe nutritional stress [Supplementary data (Fig. 9)]. Elongation factor 1α (EF-1α) was also found to be associated with ribonucleoprotein complexes with a potential role in stress response (Alves et al. 2015). Most EF-1α mRNA targets in stress condition that were found to be modulated in our data were upregulated along the growth curve [Supplementary data (Fig. 3)].

In this study, the distinct patterns of mRNA expression for several RBPs and their mRNA targets corroborate the notion that RBPs may have diverse roles in the control of gene expression, triggering or impairing important cellular and molecular processes during the growth curve. In this context, our work can provide early insights into how these genes function in the epimastigote form.

**Fig. 8:**
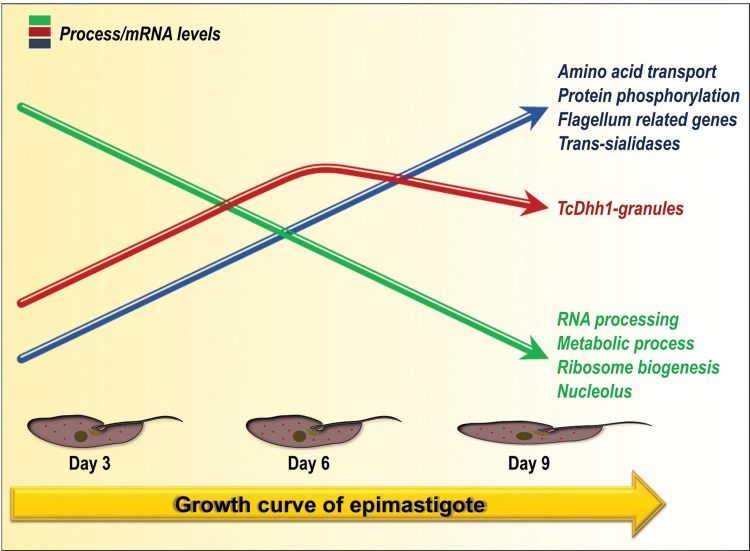
: illustration of the major characteristics that were analysed in relation to mRNA expression patterns and the process of stress granules formation in epimastigotes along the growth curve. TcDhh1 granules content increased by day 6 and decreased by day 9. Among the upregulated genes were trans-sialidases, flagellum-related genes, and genes involved in amino acid transport and protein phosphorylation. Important downregulated genes were related to RNA processing, ribosome biogenesis, nucleolus, and several metabolic processes. Illustrated parasites represent flagellar elongation that occurs during stationary phase and are not drawn to scale.

*In conclusion* - High-throughput approaches have been commonly used in recent years to study several biological aspects of organisms. RNA-Seq methods generate valuable information on regulation of gene expression on an omics scale, and accurately determine specific gene expression levels. This work represents the first assessment of gene expression modulation during the growth curve of a kinetoplastid protozoan. We have discussed some aspects of the *T. cruzi* growth curve mainly related to the stationary phase (Fig. 8), including the dynamics of RNA granule formation and the main downregulated or upregulated processes. Furthermore, we provided a significant amount of data that is available to the scientific community to gain insights into the critical processes of epimastigote development.
