# Supplementary material for: Trypanosoma cruzi transcriptome during axenic epimastigote growth curve
Source: Mem Inst Oswaldo Cruz. 2018 Apr 3;113(5):e170404. doi: 10.1590/0074-02760170404 (PMC5907844; doi:10.1590/0074-02760170404)
Supplement: Supplementary file 1 [file 0074-0276-mioc-113-5-e170404-suppl01.pdf]

Mem Inst Oswaldo Cruz, Rio de Janeiro, Vol. *113*(5): e1704041, 2018

TABLE I

RNA total sequencing date from SOLiD platform. Total number of reads generated, mapped genes and percentage ratio between reads number and mapped genes

Sample	Reads	Mapped genes	(%)
R1 d1	25,914,424	3,761,994	14.5%
R2 d1	14,423,190	4,365,746	30.3%
R3 d1	15,193,196	5,667,094	37.3%
R1 d2	8,009,108	1,267,551	15.8%
R2 d2	13,567,040	4,399,547	32.4%
R3 d2	13,511,044	4,939,298	36.6%
R1 d3	28,114,654	8,124,908	28.9%
R2 d3	9,197,397	2,038,984	22.2%
R3 d3	27,552,854	9,998,374	36.3%
R1 d4	7,240,263	1,358,227	18.8%
R2 d4	10,431,269	1,378,185	13.2%
R3 d4	13,559,166	5,273,825	38.9%
R1 d5	11,054,831	1,297,336	11.7%
R2 d5	9,393,150	3,480,423	37.1%
R1 d6	7,028,547	802,985	11.4%
R2 d6	12,316,046	1,685,044	13.7%
R3 d6	19,433,111	7,036,615	36.2%
R1 d7	18,592,523	6,771,367	36.4%
R2 d7	12,108,683	2,809,495	23.2%
R3 d7	14,299,758	4,879,346	34.1%
R1 d8	10,979,175	4,552,584	41.5%
R2 d8	11,003,776	4,593,353	41.7%
R3 d8	31,069,371	8,087,033	26.0%
R1 d9	6,491,121	2,277,764	35.1%
R2 d9	28,091,980	8,322,228	29.6%
R3 d9	12,397,971	2,787,894	22.5%
R1 d10	7,418,750	2,580,280	34.8%
R2 d10	26,134,377	9,344,508	35.8%
R3 d10	30,624,996	9,392,500	30.7%

R: biological replicates; d: day of growth.

TABLE II

RNA polysomal sequencing date from SOLiD platform. Total number of reads generated, mapped genes and percentage ratio between reads number and mapped genes

Sample	Reads	Mapped genes	(%)
R1 d1	26,629,052	625,439	2.3%
R2 d1	13,035,689	5,478,569	42.0%
R3 d1	14,258,745	5,500,564	38.6%
R1 d2	11,878,544	4,395,924	37.0%
R2 d2	15,400,642	5,638,885	36.6%
R2 d3	12,773,805	4,825,063	37.8%
R3 d3	15,798,712	5,993,916	37.9%
R1 d4	14,532,722	5,333,752	36.7%
R3 d4	11,200,086	4,544,160	40.6%
R1 d5	15,657,212	5,886,898	37.6%
R3 d5	18,145,506	6,714,337	37.0%
R2 d6	18,175,237	7,579,212	41.7%
R1 d7	15,231,345	5,503,538	36.1%
R2 d7	12,802,809	5,180,039	40.5%
R3 d7	9,215,150	209,266	2.3%
R1 d8	14,418,080	5,437,061	37.7%
R2 d8	16,996,317	5,620,611	33.1%
R3 d8	15,719,249	6,584,459	41.9%
R2 d9	15,817,710	6,548,630	41.4%
R3 d9	110,269,949	43,012,501	39.0%
R3 d10	14,189,340	5,386,171	38.0%

R: biological replicates; d: day of growth.

TABLE III

List of differentially expressed genes (DEGs) with their respective false discovery rates (FDR) for total RNA fraction

Supra gene	Representative ID	FDR
	FDR < 0.001	
	FDR < 0.01	
	FDR < 0.1	
SG1026	TcCLB.509215.40	1.01056444300841e-07
SG2525	TcCLB.506573.50	1.03018566600143e-05
SG4321	TcCLB.511529.160	1.03636558107401e-05
SG4861	TcCLB.510305.40	1.04269665988517e-07
SG6307	TcCLB.506229.64	1.06651613696932e-06
SG7928	TcCLB.506627.100	1.06651613696932e-06
SG5927	TcCLB.510575.210	1.07869870673197e-05
SG3633	TcCLB.503559.70	1.09522598565253e-06
SG4884	TcCLB.511283.124	1.0996102959851e-06
SG6867	TcCLB.504153.260	1.0996102959851e-06
SG2527	TcCLB.508661.70	1.1167321598022e-08
SG1642	TcCLB.505183.30	1.15015503587389e-11
SG4904	TcCLB.509045.20	1.17492916624975e-06
SG0120	TcCLB.506355.100	1.17859941290475e-05
SG0251	TcCLB.510581.9	1.17859941290475e-05
SG2810	TcCLB.508669.10	1.17859941290475e-05
SG4455	TcCLB.507737.20	1.17859941290475e-05
SG6463	TcCLB.509253.30	1.17859941290475e-05
SG6280	TcCLB.511635.40	1.19025062022882e-05

Supra gene	Representative ID	FDR
SG1918	TcCLB.509795.30	1.22963384070983e-06
SG3402	TcCLB.506529.50	1.22963384070983e-06
SG4814	TcCLB.506821.80	1.22963384070983e-06
SG7552	TcCLB.505683.5	1.22963384070983e-06
SG8466	TcCLB.511391.120	1.23036648028499e-05
SG0727	TcCLB.508411.50	1.25752717640162e-06
SG2870	TcCLB.510581.20	1.2637725251021e-14
SG4641	TcCLB.507765.20	1.27131810515774e-05
SG0350	TcCLB.507059.60	1.2779774511191e-06
SG6862	TcCLB.482097.20	1.28102221102764e-05
SG4292	TcCLB.508737.70	1.28828806362647e-07
SG2461	TcCLB.511383.80	1.28871595741381e-07
SG2281	TcCLB.506581.30	1.30432524993638e-05
SG4532	TcCLB.511825.240	1.30958136727905e-07
SG3266	TcCLB.510665.30	1.31216967240945e-13
SG0039	TcCLB.507753.10	1.37496949142047e-05
SG4110	TcCLB.510099.30	1.37496949142047e-05
SG2714	TcCLB.508409.160	1.38595869835385e-06
SG3838	TcCLB.507609.40	1.39190414675295e-06
SG7101	TcCLB.510091.110	1.39290571524628e-10
SG4497	TcCLB.503925.90	1.39933069887826e-06
SG4165	TcCLB.510335.10	1.40189420442077e-06
SG2306	TcCLB.509901.100	1.43330849443164e-05
SG0466	TcCLB.509065.60	1.44031591648107e-05
SG5265	TcCLB.511389.130	1.44965294437024e-05
SG1447	TcCLB.509717.60	1.46114536440967e-05
SG0346	TcCLB.504223.20	1.47484637707156e-05
SG6486	TcCLB.511545.140	1.48344980920558e-05
SG0635	TcCLB.506775.30	1.49664282500327e-06
SG5438	TcCLB.507519.164	1.53628927251928e-05
SG6155	TcCLB.511903.20	1.57548980275785e-13
SG7214	TcCLB.507629.39	1.65045047629061e-09
SG8015	TcCLB.508461.240	1.65421698248415e-06
SG4308	TcCLB.507913.20	1.70398768186271e-09
SG3440	TcCLB.510445.50	1.70951793715063e-08
SG3295	TcCLB.509161.10	1.71207854352295e-05
SG2752	TcCLB.506399.80	1.74319278372277e-05
SG4998	TcCLB.510155.20	1.74319278372277e-05
SG3769	TcCLB.509713.30	1.74619452623287e-10
SG5948	TcCLB.503733.30	1.77735657468575e-05
SG6911	TcCLB.508307.170	1.78801682422977e-07
SG0823	TcCLB.506629.220	1.79641051831574e-06
SG2286	TcCLB.508193.60	1.81772376062515e-06
SG5506	TcCLB.508387.100	1.81772376062515e-06
SG7386	TcCLB.504147.260	1.81772376062515e-06
SG6227	TcCLB.511159.14	1.84651138811437e-05
SG4732	TcCLB.506413.70	1.86377173421176e-05
SG1958	TcCLB.508399.10	1.87937279322769e-06
SG5213	TcCLB.509715.40	1.88030270797382e-05
SG1113	TcCLB.503975.50	1.89300326210863e-05
SG1740	TcCLB.509693.80	1.92062839492702e-06
SG2923	TcCLB.508257.30	1.95052402997933e-05
SG8258	TcCLB.506247.50	2.01053795153087e-05

Supra gene	Representative ID	FDR
SG2347	TcCLB.506195.290	2.0174737651711e-07
SG6152	TcCLB.510305.70	2.03736219486661e-08
SG6583	TcCLB.503925.80	2.11316315841838e-07
SG1533	TcCLB.509001.20	2.12424240452456e-06
SG2855	TcCLB.511277.60	2.16136115758532e-14
SG5368	TcCLB.508507.40	2.16497044245886e-08
SG2025	TcCLB.507515.90	2.17464056324085e-05
SG2176	TcCLB.509053.70	2.20473708743508e-07
SG6096	TcCLB.509643.70	2.20473708743508e-07
SG2247	TcCLB.503735.40	2.2588763169519e-05
SG2297	TcCLB.510225.40	2.2588763169519e-05
SG4726	TcCLB.510065.40	2.2588763169519e-05
SG6972	TcCLB.510431.230	2.26130669858727e-06
SG2477	TcCLB.511529.68	2.28034800694424e-07
SG3123	TcCLB.508981.20	2.28034800694424e-07
SG4075	TcCLB.508547.140	2.28252759091974e-05
SG5274	TcCLB.506775.180	2.28382385949458e-05
SG3764	TcCLB.508273.30	2.30173474030742e-05
SG1074	TcCLB.511899.40	2.34541008895979e-11
SG1279	TcCLB.504173.30	2.36970466836888e-06
SG0145	TcCLB.511911.90	2.41297253167476e-07
SG8187	TcCLB.510687.120	2.41297253167476e-07
SG8124	TcCLB.511003.160	2.50225005471539e-07
SG3882	TcCLB.506821.110	2.54843919429066e-06
SG6408	TcCLB.507081.130	2.54843919429066e-06
SG5058	TcCLB.503829.10	2.57477607334454e-06
SG0042	TcCLB.510787.10	2.63696971356176e-05
SG3403	TcCLB.508111.30	2.63696971356176e-05
SG4050	TcCLB.506193.60	2.63696971356176e-05
SG4260	TcCLB.505983.20	2.63696971356176e-05
SG2142	TcCLB.509429.280	2.63772514828082e-06
SG0868	TcCLB.504625.70	2.68085407931893e-08
SG3526	TcCLB.508909.20	2.68085407931893e-08
SG7922	TcCLB.510873.10	2.68085407931893e-08
SG8729	TcCLB.506247.40	2.68085407931893e-08
SG3320	TcCLB.511355.30	2.70196229937254e-07
SG2393	TcCLB.511281.40	2.70745401945982e-11
SG5360	TcCLB.509161.20	2.70931247547749e-06
SG4095	TcCLB.506479.120	2.71211015575622e-06
SG6201	TcCLB.508153.1140	2.72657337523708e-06
SG4695	TcCLB.511431.54	2.74076485662872e-05
SG2229	TcCLB.510039.64	2.74085480670086e-08
SG7049	TcCLB.511421.170	2.75661397564418e-07
SG2986	TcCLB.506195.300	2.85554381854888e-08
SG4581	TcCLB.509051.40	2.85554381854888e-08
SG8143	TcCLB.511807.125	2.85554381854888e-08
SG1587	TcCLB.510647.30	2.88151808420383e-08
SG4182	TcCLB.509499.10	2.88151808420383e-08
SG0148	TcCLB.511325.40	2.89218316812259e-11
SG1829	TcCLB.507057.40	2.95017660167688e-06
SG2422	TcCLB.508593.130	2.97151159352258e-06
SG0514	TcCLB.508015.10	2.98719404968148e-06
SG3246	TcCLB.511295.60	3.04726679823411e-07

Supra gene	Representative ID	FDR
SG2358	TcCLB.421619.10	3.20181370251771e-05
SG0476	TcCLB.504191.10	3.20365980897923e-05
SG4526	TcCLB.504087.10	3.32426269798743e-05
SG1784	TcCLB.504021.100	3.34287836501881e-07
SG8968	TcCLB.509105.90	3.38095690968622e-06
SG5740	TcCLB.436521.9	3.5026999922761e-05
SG0828	TcCLB.508961.20	3.51087526596532e-06
SG4159	TcCLB.511277.100	3.57312100217816e-10
SG7457	TcCLB.511439.70	3.57312100217816e-10
SG3604	TcCLB.507023.50	3.67168011248812e-05
SG4757	TcCLB.511761.60	3.67168011248812e-05
SG8050	TcCLB.508017.30	3.67409656125025e-06
SG8806	TcCLB.508461.410	3.77750845237401e-06
SG1789	TcCLB.509551.70	3.78516352589202e-09
SG5521	TcCLB.511819.30	3.78517848166758e-05
SG5855	TcCLB.507927.80	3.801931620843e-05
SG6238	TcCLB.503809.75	3.80726326295479e-05
SG0081	TcCLB.504015.30	3.88736749442746e-11
SG6761	TcCLB.504213.100	3.96182842098215e-06
SG5133	TcCLB.508917.30	3.99551576122465e-05
SG3070	TcCLB.509937.170	4.00944464974503e-05
SG6323	TcCLB.509337.19	4.01442680182237e-05
SG2699	TcCLB.506355.130	4.13055258020184e-05
SG7815	TcCLB.508971.30	4.15816079138076e-06
SG2453	TcCLB.504001.20	4.22353529583195e-06
SG7726	TcCLB.510977.9	4.25371061987708e-07
SG3684	TcCLB.511151.54	4.36205176300856e-05
SG7998	TcCLB.511511.3	4.36205176300856e-05
SG4574	TcCLB.509599.60	4.366132367914e-05
SG5578	TcCLB.506025.40	4.36898241455572e-06
SG4053	TcCLB.504255.20	4.38045451320531e-08
SG6371	TcCLB.507787.140	4.38045451320531e-08
SG7820	TcCLB.504131.60	4.47701218704361e-05
SG8239	TcCLB.508479.274	4.52594431582249e-06
SG0297	TcCLB.511575.130	4.53590733380483e-05
SG0766	TcCLB.509049.30	4.56743338864575e-05
SG0304	TcCLB.506469.80	4.60101452522282e-09
SG8302	TcCLB.506335.130	4.64304207892595e-05
SG5732	TcCLB.509789.40	4.64769467094346e-06
SG8318	TcCLB.508799.240	4.66200925876879e-07
SG4358	TcCLB.511517.164	4.7380003984662e-05
SG3677	TcCLB.508817.10	4.79215771118602e-07
SG6597	TcCLB.509999.140	4.79709639809834e-05
SG1862	TcCLB.506443.70	4.82692750832172e-09
SG5464	TcCLB.508737.150	4.88933885839239e-10
SG4548	TcCLB.509237.50	4.90874097938717e-06
SG2002	TcCLB.503879.110	4.91140881054754e-06
SG2991	TcCLB.508269.40	4.91902708691792e-06
SG8527	TcCLB.511491.70	4.91902708691792e-06
SG3329	TcCLB.508307.180	4.92757510726064e-12
SG6522	TcCLB.509179.70	5.10665592012775e-05
SG1575	TcCLB.508999.260	5.28078281089821e-05
SG8807	TcCLB.508173.180	5.28078281089821e-05

Supra gene	Representative ID	FDR
SG0306	TcCLB.506201.70	5.38640833317888e-06
SG2362	TcCLB.508859.80	5.48052811970786e-05
SG8762	TcCLB.511367.240	5.51598141670676e-06
SG6762	TcCLB.511189.70	5.66569533207648e-07
SG5075	TcCLB.504161.50	5.66960543319985e-07
SG0084	TcCLB.506551.10	5.74428444458141e-06
SG3321	TcCLB.509003.30	5.76275755920617e-08
SG2267	TcCLB.506835.40	5.76883669879035e-05
SG2057	TcCLB.510735.40	5.86059394107253e-05
SG2389	TcCLB.506211.250	5.92183788711253e-11
SG1276	TcCLB.509601.140	6.05744244364385e-07
SG8723	TcCLB.508667.40	6.12989415116471e-06
SG3079	TcCLB.508917.20	6.15190022559121e-05
SG8505	TcCLB.508257.180	6.15324375038038e-05
SG2627	TcCLB.511127.180	6.19292141731651e-05
SG1540	TcCLB.509053.30	6.24478543274979e-05
SG2985	TcCLB.510149.140	6.24478543274979e-05
SG3061	TcCLB.511807.64	6.24478543274979e-05
SG8730	TcCLB.509647.180	6.24478543274979e-05
SG2019	TcCLB.509109.160	6.30488123945194e-05
SG0380	TcCLB.511757.70	6.56388464887519e-06
SG2054	TcCLB.507801.130	6.58855422133355e-05
SG6879	TcCLB.506635.139	6.80669411762092e-06
SG7394	TcCLB.507099.30	6.95013469562337e-07
SG7223	TcCLB.441241.10	6.96020857724748e-07
SG3072	TcCLB.509033.80	6.97058426443546e-06
SG3859	TcCLB.510397.10	6.99218779670384e-05
SG2513	TcCLB.509059.50	7.01406021196298e-05
SG2015	TcCLB.507241.30	7.0231494620328e-06
SG7626	TcCLB.503419.50	7.10469389251848e-05
SG8461	TcCLB.508479.370	7.15996441667255e-07
SG5443	TcCLB.509151.90	7.16471627191549e-08
SG5338	TcCLB.503653.60	7.20074504721647e-07
SG5829	TcCLB.508153.164	7.24341517438601e-05
SG4950	TcCLB.508445.90	7.25072180381048e-05
SG3001	TcCLB.508257.70	7.28056026171613e-05
SG2683	TcCLB.508153.820	7.28163743649029e-05
SG1620	TcCLB.511277.539	7.37700204452579e-05
SG0846	TcCLB.508065.70	7.39573063363406e-11
SG3947	TcCLB.507031.29	7.44412712555917e-08
SG6020	TcCLB.508965.39	7.44412712555917e-08
SG2257	TcCLB.507049.100	7.54735350443382e-05
SG2690	TcCLB.507031.64	7.72668697986093e-05
SG2327	TcCLB.506625.120	7.76385072584385e-07
SG2285	TcCLB.507659.10	7.77876934300435e-06
SG1548	TcCLB.511865.40	7.8627739043801e-05
SG5362	TcCLB.509471.30	7.8627739043801e-05
SG7876	TcCLB.506629.40	7.88127075868704e-06
SG5193	TcCLB.511017.25	7.99510835636018e-06
SG7857	TcCLB.508173.100	8.00785289106158e-08
SG5685	TcCLB.511823.70	8.19797080222239e-05
SG7583	TcCLB.511421.210	8.35425400078286e-09
SG7901	TcCLB.511909.30	8.36948852094795e-05

Supra gene	Representative ID	FDR
SG5710	TcCLB.509319.20	8.42222912419568e-08
SG8006	TcCLB.507053.170	8.52000228155904e-05
SG3256	TcCLB.507159.14	8.55999147088633e-07
SG0245	TcCLB.511657.20	8.64457214795336e-06
SG2910	TcCLB.507963.10	8.69306479164945e-05
SG8224	TcCLB.511367.210	8.70698823488967e-09
SG5858	TcCLB.504229.110	8.81077751693698e-05
SG7933	TcCLB.510187.290	8.83093195168873e-08
SG3115	TcCLB.511321.60	8.91807975149179e-07
SG7589	TcCLB.503975.40	8.93727212092524e-07
SG5717	TcCLB.505997.200	8.9622590220625e-08
SG0638	TcCLB.510719.20	9.07980436174219e-05
SG8127	TcCLB.508479.350	9.16070765986115e-07
SG2028	TcCLB.509505.40	9.18511022126231e-09
SG1392	TcCLB.506989.30	9.43832862029548e-05
SG3990	TcCLB.511827.100	9.43832862029548e-05
SG5915	TcCLB.503975.100	9.43832862029548e-05
SG6383	TcCLB.509733.70	9.43832862029548e-05
SG7106	TcCLB.509831.33	9.43832862029548e-05
SG7272	TcCLB.508307.150	9.48902148819923e-05
SG2924	TcCLB.510409.20	9.49078501921184e-05
SG1304	TcCLB.508707.130	9.53600027127768e-05
SG6369	TcCLB.504797.80	9.53600027127768e-05
SG5641	TcCLB.506543.10	9.54590175028695e-05
SG8386	TcCLB.510689.30	9.68879930771321e-12
SG0814	TcCLB.508909.330	9.75825465675192e-06
SG8613	TcCLB.510303.200	9.90469612444901e-06
SG8724	TcCLB.506679.20	9.92429315191171e-06
SG3583	TcCLB.508461.40	0.000113411071277938
SG1528	TcCLB.504105.160	0.000115226111299698
SG4131	TcCLB.507007.74	0.000115226111299698
SG2793	TcCLB.509229.60	0.000115796327763553
SG4040	TcCLB.508601.90	0.000117145898889255
SG1031	TcCLB.507811.30	0.000124944690850237
SG0072	TcCLB.505699.10	0.00012939106977822
SG4441	TcCLB.506263.40	0.000129756518487277
SG5059	TcCLB.505945.60	0.000133020305171217
SG0560	TcCLB.504255.30	0.000136155138955973
SG1761	TcCLB.506581.10	0.000136811916379176
SG0441	TcCLB.505999.10	0.000141564177607332
SG2117	TcCLB.509563.10	0.000141564177607332
SG4320	TcCLB.508699.40	0.000141564177607332
SG4924	TcCLB.508851.160	0.000141564177607332
SG7706	TcCLB.510187.250	0.000145700916926008
SG1812	TcCLB.509601.20	0.000151680854644768
SG7098	TcCLB.506925.10	0.000151735933486557
SG6294	TcCLB.503599.10	0.000156800163733703
SG3964	TcCLB.507711.50	0.000158129240484591
SG7690	TcCLB.511283.184	0.000158667185572096
SG1942	TcCLB.508153.260	0.000159110380004006
SG3564	TcCLB.509029.80	0.000159395619655299
SG4556	TcCLB.506777.20	0.000159621795370686
SG3542	TcCLB.510001.10	0.000162611922368521

Supra gene	Representative ID	FDR
SG8736	TcCLB.511725.174	0.000165010379593963
SG0310	TcCLB.506153.10	0.000167843108126103
SG6948	TcCLB.509007.50	0.00017459852510774
SG5199	TcCLB.506991.10	0.000186235838317282
SG5811	TcCLB.507017.64	0.000186235838317282
SG6595	TcCLB.407335.9	0.000186535379775839
SG4228	TcCLB.510829.10	0.000190545392398022
SG1315	TcCLB.509207.120	0.000194790458083441
SG7609	TcCLB.508813.80	0.000200062071591417
SG4962	TcCLB.507951.170	0.000202160913188388
SG5639	TcCLB.506181.10	0.000202160913188388
SG0661	TcCLB.458241.10	0.000203713006337639
SG7990	TcCLB.507949.250	0.000203713006337639
SG4501	TcCLB.503945.40	0.000207525225700087
SG3283	TcCLB.508153.640	0.000209117044486174
SG1926	TcCLB.510667.60	0.000210173379678997
SG1996	TcCLB.508909.10	0.000210173379678997
SG0749	TcCLB.504051.40	0.000212251845498371
SG3585	TcCLB.508153.454	0.000212251845498371
SG5209	TcCLB.511289.70	0.000212251845498371
SG5309	TcCLB.509569.40	0.000212251845498371
SG7988	TcCLB.509647.50	0.000212251845498371
SG4508	TcCLB.503465.30	0.000214167871682298
SG5645	TcCLB.509429.120	0.000214167871682298
SG5640	TcCLB.510421.180	0.00021732084146479
SG6491	TcCLB.506241.80	0.000224676089154083
SG5852	TcCLB.507677.150	0.000229938540827474
SG3861	TcCLB.510423.50	0.000234439199553835
SG3011	TcCLB.504085.20	0.000235554894451083
SG3930	TcCLB.506301.20	0.000237063606113338
SG2892	TcCLB.508831.66	0.000248812092926471
SG0377	TcCLB.508621.10	0.000251327348946487
SG2600	TcCLB.503923.30	0.000251327348946487
SG0281	TcCLB.509639.10	0.000254687745300747
SG4422	TcCLB.507305.40	0.000254687745300747
SG5170	TcCLB.504069.90	0.000254687745300747
SG5769	TcCLB.511181.60	0.000261769715632981
SG2474	TcCLB.509561.70	0.000267199188024254
SG7549	TcCLB.510297.119	0.000267199188024254
SG0848	TcCLB.508461.80	0.000272736518907165
SG2322	TcCLB.506825.100	0.000272736518907165
SG5294	TcCLB.503891.40	0.000272736518907165
SG6675	TcCLB.510339.100	0.000280587931224313
SG2930	TcCLB.511481.70	0.000284631116731022
SG5701	TcCLB.508175.90	0.000287353510745692
SG4271	TcCLB.508577.140	0.000303928551768393
SG7066	TcCLB.510513.70	0.000305498566820537
SG7516	TcCLB.506625.70	0.000308113021332929
SG0533	TcCLB.506107.10	0.000319566669180563
SG2819	TcCLB.511857.50	0.000326546594620313
SG5960	TcCLB.505183.84	0.000326546594620313
SG5419	TcCLB.510221.20	0.00032776501617883
SG7393	TcCLB.504423.15	0.00032776501617883

Supra gene	Representative ID	FDR
SG1345	TcCLB.506825.200	0.000334622452159167
SG6057	TcCLB.509695.50	0.000334622452159167
SG3594	TcCLB.504125.64	0.000339706351607527
SG3189	TcCLB.511499.59	0.000342909230402351
SG5541	TcCLB.506999.120	0.000342909230402351
SG1589	TcCLB.508999.250	0.000343166678284028
SG3474	TcCLB.511181.20	0.000353317489742391
SG7638	TcCLB.508257.220	0.000354076315231367
SG1407	TcCLB.510359.290	0.000356060970529331
SG1850	TcCLB.510155.10	0.000356060970529331
SG5943	TcCLB.511653.20	0.000356060970529331
SG8482	TcCLB.511655.20	0.000356060970529331
SG2096	TcCLB.510155.160	0.000361280103755018
SG0593	TcCLB.504053.10	0.000363224370856328
SG6439	TcCLB.508273.70	0.000364949072112695
SG7919	TcCLB.511367.138	0.000364949072112695
SG4938	TcCLB.503431.90	0.000368810431465046
SG5586	TcCLB.508153.920	0.000369727937586257
SG2428	TcCLB.506739.200	0.000383385184863792
SG6185	TcCLB.509859.50	0.000386504747268651
SG6134	TcCLB.509791.30	0.000397814759628858
SG5007	TcCLB.509109.114	0.000400514569019696
SG6564	TcCLB.511323.60	0.000403317693826751
SG0049	TcCLB.510275.255	0.000408086432704943
SG4603	TcCLB.511257.30	0.000408086432704943
SG8660	TcCLB.511727.140	0.000408086432704943
SG6176	TcCLB.511559.50	0.000411697256290668
SG0040	TcCLB.511643.110	0.000412854388924951
SG4485	TcCLB.510329.330	0.000415867217425011
SG8878	TcCLB.507019.60	0.000429937188471998
SG0717	TcCLB.506287.80	0.000430623763030432
SG6387	TcCLB.504427.50	0.000432312632418745
SG7867	TcCLB.508409.110	0.000432312632418745
SG6906	TcCLB.505073.10	0.000439799398327124
SG2090	TcCLB.510659.44	0.000442262874618575
SG0887	TcCLB.506851.10	0.000446700531560587
SG1463	TcCLB.510729.230	0.000446700531560587
SG0980	TcCLB.504069.56	0.000447496203570687
SG3790	TcCLB.506009.20	0.000447496203570687
SG0268	TcCLB.509239.10	0.000448523173872932
SG2226	TcCLB.504125.100	0.000465835894685788
SG1082	TcCLB.509835.10	0.000468414396520983
SG4179	TcCLB.509791.60	0.000468414396520983
SG7153	TcCLB.510859.17	0.000472281870436794
SG0452	TcCLB.503731.9	0.00047694374781764
SG6489	TcCLB.503917.7	0.000484191323208449
SG1017	TcCLB.504199.10	0.000486698085922064
SG8971	TcCLB.511249.44	0.000486698085922064
SG3356	TcCLB.510303.60	0.000492890911544352
SG2451	TcCLB.503409.10	0.000496454385526839
SG1355	TcCLB.503565.29	0.000515395036135195
SG2828	TcCLB.504199.20	0.000515395036135195
SG2541	TcCLB.509099.150	0.000526059235423362

Supra gene	Representative ID	FDR
SG6423	TcCLB.509791.100	0.000526922316690177
SG2387	TcCLB.506175.50	0.000531007425237288
SG1233	TcCLB.510099.120	0.000551517009630718
SG3966	TcCLB.511031.40	0.000551517009630718
SG6478	TcCLB.509937.20	0.00057512783582601
SG1257	TcCLB.504949.30	0.000575623728637971
SG3285	TcCLB.509003.40	0.000581635450662447
SG5962	TcCLB.510323.60	0.000586949085540555
SG4721	TcCLB.510303.50	0.000588270489323341
SG4488	TcCLB.506477.20	0.000592130218180341
SG3827	TcCLB.507049.20	0.000599903435032779
SG4920	TcCLB.503681.20	0.000602398412455305
SG7392	TcCLB.504125.60	0.000630962636832953
SG2881	TcCLB.504129.40	0.000638924403208571
SG6217	TcCLB.503955.100	0.000650347229989038
SG0708	TcCLB.507009.30	0.000660198775336808
SG3678	TcCLB.510899.50	0.000660198775336808
SG4590	TcCLB.508231.200	0.000660198775336808
SG3377	TcCLB.511903.170	0.000662897491645456
SG0020	TcCLB.510583.80	0.000676746639247965
SG5421	TcCLB.511383.50	0.000687473921479146
SG7402	TcCLB.504057.130	0.00068893243291224
SG5456	TcCLB.508351.40	0.000690240152778556
SG6230	TcCLB.510729.140	0.000697413824557236
SG7828	TcCLB.506577.5	0.000708041767387593
SG0095	TcCLB.510783.20	0.000717101618010216
SG0669	TcCLB.504023.10	0.000717101618010216
SG0829	TcCLB.511821.170	0.000717101618010216
SG4683	TcCLB.510667.120	0.000717101618010216
SG3146	TcCLB.507275.40	0.000730786175191966
SG4602	TcCLB.511165.100	0.000758206896760016
SG5232	TcCLB.403875.10	0.000763438574236463
SG5556	TcCLB.510091.100	0.000765121490466007
SG2321	TcCLB.506825.80	0.000809646994732127
SG3362	TcCLB.506315.80	0.000809646994732127
SG3249	TcCLB.510349.20	0.000809747803007318
SG0121	TcCLB.511401.90	0.000814945420239379
SG1008	TcCLB.509237.90	0.000814945420239379
SG5713	TcCLB.506713.14	0.000819598155523427
SG7274	TcCLB.509895.20	0.00082192505982217
SG8668	TcCLB.507209.10	0.000828384770780291
SG6892	TcCLB.509705.10	0.00082984370278698
SG6407	TcCLB.511353.10	0.000830197325146995
SG5647	TcCLB.507061.34	0.000846866487584512
SG3234	TcCLB.506289.60	0.00084722497179096
SG0677	TcCLB.504125.80	0.000856076750958695
SG4394	TcCLB.511181.80	0.000856076750958695
SG3779	TcCLB.506709.10	0.000860184013475659
SG4265	TcCLB.511407.40	0.00086067827412805
SG6362	TcCLB.510311.100	0.000876072543996713
SG5650	TcCLB.503929.20	0.000896525307261925
SG1609	TcCLB.509595.30	0.000938078013415483
SG5418	TcCLB.510339.54	0.000939273312104305

Supra gene	Representative ID	FDR
SG6440	TcCLB.507089.30	0.000941514567993354
SG0884	TcCLB.485683.10	0.000948463805251628
SG7102	TcCLB.503865.60	0.000954477569366741
SG2772	TcCLB.508637.120	0.000957222346309509
SG3449	TcCLB.510861.70	0.000957222346309509
SG7003	TcCLB.508727.24	0.000957222346309509
SG3397	TcCLB.510357.80	0.000961024202008758
SG7824	TcCLB.506993.160	0.000961024202008758
SG3943	TcCLB.510155.94	0.000965262076344066
SG1122	TcCLB.504019.3	0.000969860932850609
SG6749	TcCLB.504117.50	0.000982167013737877
SG1896	TcCLB.503773.24	0.000984442637043313
SG4689	TcCLB.506975.20	0.000984442637043313
SG0230	TcCLB.506789.334	0.000987341395632467
SG6989	TcCLB.506885.90	0.000987341395632467
SG2499	TcCLB.511593.20	0.0010025986937168
SG2099	TcCLB.510155.115	0.00100681167957184
SG2700	TcCLB.509167.20	0.00103663407708214
SG3315	TcCLB.506791.10	0.00105429750575586
SG1607	TcCLB.511647.10	0.00107913273943214
SG0776	TcCLB.510003.20	0.00108532315749467
SG4813	TcCLB.506727.110	0.00109101433127715
SG0327	TcCLB.511385.110	0.00113696667380214
SG4322	TcCLB.506931.70	0.00113696667380214
SG5512	TcCLB.508741.370	0.00114261209822271
SG2395	TcCLB.510887.30	0.00115691167635128
SG0115	TcCLB.510795.10	0.00119091582700703
SG3844	TcCLB.506999.180	0.00119610937771672
SG5721	TcCLB.507031.90	0.00119610937771672
SG8531	TcCLB.511725.230	0.00119610937771672
SG0879	TcCLB.503531.40	0.00119895588356781
SG6374	TcCLB.510877.140	0.00119895588356781
SG1634	TcCLB.506727.140	0.00120977474273555
SG0101	TcCLB.506499.105	0.00123888203502991
SG4013	TcCLB.506175.10	0.00123888203502991
SG4169	TcCLB.507491.119	0.00123888203502991
SG2917	TcCLB.508593.30	0.00126737501065541
SG4150	TcCLB.510311.30	0.00126823148112674
SG5969	TcCLB.511353.50	0.00126823148112674
SG6686	TcCLB.508637.160	0.00126823148112674
SG7444	TcCLB.508647.174	0.00126823148112674
SG1998	TcCLB.508707.50	0.0012888104596629
SG4628	TcCLB.507017.70	0.00130692276016585
SG1780	TcCLB.506401.280	0.00130794066786695
SG2039	TcCLB.511025.70	0.00130794066786695
SG2302	TcCLB.509207.90	0.00130794066786695
SG4385	TcCLB.508891.30	0.00130794066786695
SG8682	TcCLB.504109.140	0.00131839604523259
SG2661	TcCLB.509695.100	0.00135161828517396
SG3311	TcCLB.509749.30	0.00135699608907242
SG1085	TcCLB.507669.110	0.00137370058790978
SG5304	TcCLB.510357.10	0.00137779092409794
SG0527	TcCLB.506357.130	0.00139871350503308

Supra gene	Representative ID	FDR
SG0620	TcCLB.509999.120	0.00139871350503308
SG1413	TcCLB.503515.14	0.00140985922997773
SG8306	TcCLB.508707.200	0.00141205851292071
SG1362	TcCLB.509573.10	0.00141776104788085
SG8248	TcCLB.506985.50	0.00141776104788085
SG2351	TcCLB.508593.100	0.00142374895271999
SG2701	TcCLB.506443.40	0.00142374895271999
SG2911	TcCLB.506475.113	0.00142374895271999
SG5555	TcCLB.511277.140	0.00142374895271999
SG1705	TcCLB.510689.80	0.00143832031612459
SG1823	TcCLB.510285.20	0.00144568799864631
SG1366	TcCLB.508799.70	0.00147652953999608
SG2416	TcCLB.508269.60	0.00147652953999608
SG3998	TcCLB.509509.50	0.00147652953999608
SG2367	TcCLB.503595.10	0.00148046914449595
SG5797	TcCLB.503943.14	0.00148046914449595
SG2097	TcCLB.503939.50	0.00148110392430497
SG3853	TcCLB.508641.184	0.00148110392430497
SG6334	TcCLB.503781.30	0.00148110392430497
SG6840	TcCLB.506285.30	0.00148110392430497
SG2565	TcCLB.509945.24	0.00150031331264031
SG1781	TcCLB.511167.20	0.00150362497858572
SG1347	TcCLB.504113.30	0.0015161768560001
SG1038	TcCLB.508645.40	0.0015169985964491
SG2995	TcCLB.511287.130	0.00152879235157445
SG8940	TcCLB.506559.434	0.0015493487706853
SG7606	TcCLB.427247.10	0.00156771124781898
SG2329	TcCLB.509055.50	0.00157118754172063
SG2789	TcCLB.506691.60	0.00157118754172063
SG1124	TcCLB.508823.50	0.00159398945156369
SG4679	TcCLB.511167.110	0.00159398945156369
SG7753	TcCLB.506977.80	0.00159398945156369
SG8398	TcCLB.504071.110	0.00159398945156369
SG2977	TcCLB.506221.120	0.00160076312564989
SG7720	TcCLB.510733.50	0.00161378175455046
SG0702	TcCLB.507659.20	0.00161493442935634
SG3019	TcCLB.506241.160	0.00162528336217694
SG6079	TcCLB.504741.250	0.00162693343102312
SG8081	TcCLB.508153.184	0.00162973837506034
SG4507	TcCLB.509799.20	0.0016408504956081
SG8401	TcCLB.511725.134	0.00166887872533114
SG8052	TcCLB.506679.80	0.00167475599249688
SG3978	TcCLB.511287.90	0.00172760351156448
SG6637	TcCLB.506559.524	0.0017378574664376
SG5286	TcCLB.506593.40	0.00175066835918659
SG1524	TcCLB.508909.300	0.00175967706379931
SG4638	TcCLB.510659.270	0.00180785487419403
SG1541	TcCLB.506579.10	0.0018096864432437
SG7978	TcCLB.509857.40	0.00181091488089181
SG8669	TcCLB.511367.170	0.00181091488089181
SG5604	TcCLB.511459.50	0.00186093604677333
SG5299	TcCLB.509065.140	0.00186137285380764
SG3109	TcCLB.506295.130	0.00189787031471231

Supra gene	Representative ID	FDR
SG1416	TcCLB.509213.110	0.0019278017941305
SG0330	TcCLB.510763.30	0.0019449961494174
SG2630	TcCLB.511517.130	0.00195570352471526
SG5238	TcCLB.507943.60	0.00195570352471526
SG5742	TcCLB.510007.20	0.00196082715610032
SG5112	TcCLB.506363.110	0.00196112944384983
SG7730	TcCLB.511391.110	0.00201545858251748
SG3689	TcCLB.509879.40	0.00201683796908789
SG6827	TcCLB.506925.510	0.0020200716867345
SG1993	TcCLB.503465.40	0.00203404437528953
SG1994	TcCLB.504153.30	0.00203404437528953
SG4384	TcCLB.510333.10	0.00203404437528953
SG2313	TcCLB.504003.30	0.00206952155450176
SG3084	TcCLB.511529.50	0.00206952155450176
SG6406	TcCLB.508153.520	0.00208007363759711
SG5968	TcCLB.508999.40	0.00209201682326367
SG5619	TcCLB.508965.70	0.00214473118161577
SG8020	TcCLB.510129.20	0.00223474361787102
SG2520	TcCLB.508973.60	0.00224420430462155
SG1377	TcCLB.508405.50	0.00226400578985617
SG2239	TcCLB.511805.20	0.0022701280211525
SG2965	TcCLB.509429.130	0.0022701280211525
SG6219	TcCLB.511465.20	0.0022701280211525
SG6576	TcCLB.510859.10	0.0022701280211525
SG3644	TcCLB.507831.60	0.00227785078238929
SG8488	TcCLB.508815.120	0.00229603821329914
SG4767	TcCLB.503625.10	0.00230568269982701
SG8485	TcCLB.511907.270	0.0023165991971216
SG4378	TcCLB.507951.140	0.00231864836439036
SG7791	TcCLB.509105.130	0.00231905518252907
SG6791	TcCLB.506945.240	0.00232642524469532
SG8889	TcCLB.508879.160	0.00234771019622625
SG6496	TcCLB.506297.350	0.00235250982332827
SG3159	TcCLB.511761.50	0.00236610781482683
SG7707	TcCLB.510187.280	0.00236944902547051
SG8670	TcCLB.503603.10	0.00236944902547051
SG0392	TcCLB.509237.120	0.00238558414070001
SG6214	TcCLB.506855.350	0.00238558414070001
SG2908	TcCLB.510395.20	0.00239007958043842
SG0037	TcCLB.506435.370	0.00241882226900596
SG7365	TcCLB.503479.70	0.00243110950326525
SG1330	TcCLB.511583.10	0.00247768134035095
SG3887	TcCLB.510667.80	0.0024780707113297
SG2738	TcCLB.509023.50	0.0025238480500148
SG3393	TcCLB.511635.30	0.00253701398563633
SG4362	TcCLB.510031.70	0.00260233491339933
SG4860	TcCLB.505071.140	0.00260233491339933
SG5673	TcCLB.506123.30	0.00260330688481149
SG7572	TcCLB.506925.540	0.00262076156712293
SG0044	TcCLB.511593.40	0.00262143668878857
SG1560	TcCLB.509643.100	0.00264816561374491
SG8347	TcCLB.510889.100	0.00266950667409964
SG1251	TcCLB.511857.10	0.00267718817968389

Supra gene	Representative ID	FDR
SG2093	TcCLB.504431.30	0.00267732905765635
SG6601	TcCLB.511633.79	0.00267732905765635
SG6392	TcCLB.511033.20	0.00270754944410044
SG6500	TcCLB.509997.60	0.00272175904858488
SG6090	TcCLB.509229.10	0.00275319895262949
SG0269	TcCLB.508671.20	0.00275641135165448
SG3651	TcCLB.509717.100	0.00276965402688302
SG4317	TcCLB.510507.50	0.00276965402688302
SG0311	TcCLB.509599.130	0.00277087109685899
SG7853	TcCLB.510901.170	0.00277735817945062
SG6404	TcCLB.511903.90	0.00278746852471374
SG8704	TcCLB.511259.50	0.00278746852471374
SG2796	TcCLB.509631.160	0.00281393515240322
SG1919	TcCLB.507143.60	0.00283306131118543
SG5505	TcCLB.510425.28	0.00284035147144793
SG5235	TcCLB.508799.150	0.00284090418329824
SG8016	TcCLB.507073.30	0.00284090418329824
SG1605	TcCLB.509937.220	0.00284322368558198
SG2596	TcCLB.506985.40	0.00284837014729528
SG2833	TcCLB.511653.40	0.00288323071818906
SG6075	TcCLB.510663.40	0.00289004341163929
SG0257	TcCLB.509201.15	0.0029171775712253 1
SG7302	TcCLB.504147.240	0.00291936355546668
SG0882	TcCLB.509029.40	0.00295729491291614
SG3438	TcCLB.503923.10	0.00295729491291614
SG6785	TcCLB.506529.400	0.0030298004269024
SG5838	TcCLB.508501.250	0.00306052009943191
SG4172	TcCLB.506327.80	0.00308519121340538
SG8722	TcCLB.511367.159	0.00309312309691449
SG4751	TcCLB.508777.40	0.00316023176520497
SG4971	TcCLB.509229.130	0.00318356752625099
SG2489	TcCLB.509099.130	0.00319829482952214
SG6484	TcCLB.506871.180	0.00320446287413002
SG2237	TcCLB.506855.130	0.00321704888746975
SG3258	TcCLB.511127.340	0.00323998625030725
SG4408	TcCLB.506905.30	0.00328564526104176
SG7173	TcCLB.503955.80	0.00329865039844109
SG2038	TcCLB.506425.80	0.00334441729864586
SG3179	TcCLB.506491.10	0.0033718269377602
SG0747	TcCLB.511303.40	0.00338866771802726
SG1874	TcCLB.510149.120	0.00338866771802726
SG8089	TcCLB.506445.100	0.00341155135396439
SG1835	TcCLB.507583.20	0.00342279216748633
SG1165	TcCLB.506825.70	0.00342566126628011
SG8029	TcCLB.511491.40	0.00353428486187771
SG3538	TcCLB.503687.30	0.00362478270791825
SG3457	TcCLB.511167.60	0.00367954241785975
SG5196	TcCLB.510667.10	0.00368640357268502
SG7492	TcCLB.508837.160	0.00369120832805509
SG7262	TcCLB.504153.140	0.00369707434337389
SG4064	TcCLB.510691.70	0.00369848162755132
SG8942	TcCLB.511511.5	0.00374718777549154
SG3409	TcCLB.508919.94	0.0038387877757389

Supra gene	Representative ID	FDR
SG5693	TcCLB.511745.60	0.0038387877757389
SG6154	TcCLB.508641.180	0.00389321498840107
SG2049	TcCLB.508741.380	0.00400672992754516
SG4018	TcCLB.508613.30	0.00403560157458711
SG2363	TcCLB.504137.50	0.00406162092118613
SG4754	TcCLB.503459.10	0.00406162092118613
SG4109	TcCLB.508717.36	0.00409624294035188
SG3755	TcCLB.511517.30	0.00412606811574631
SG3485	TcCLB.503987.39	0.00414374030273034
SG3846	TcCLB.506629.20	0.00417835647999747
SG7777	TcCLB.508173.70	0.00417835647999747
SG0052	TcCLB.506289.140	0.00419859665924364
SG6694	TcCLB.508983.20	0.00423564102374228
SG5557	TcCLB.510421.270	0.00423919244155913
SG8743	TcCLB.511317.50	0.00423919244155913
SG6207	TcCLB.510719.120	0.00426043354145699
SG4130	TcCLB.506871.70	0.00430861477683285
SG4437	TcCLB.503697.70	0.00430861477683285
SG2732	TcCLB.506755.50	0.00437825839850949
SG6025	TcCLB.511257.10	0.00437825839850949
SG4090	TcCLB.511285.40	0.00439878343755249
SG8867	TcCLB.511277.180	0.00439878343755249
SG0439	TcCLB.508821.50	0.00440506088165993
SG7770	TcCLB.511367.60	0.00444584700414622
SG0400	TcCLB.510571.30	0.00445918364761605
SG1057	TcCLB.510007.50	0.00445918364761605
SG6469	TcCLB.510359.170	0.00445918364761605
SG0712	TcCLB.506559.129	0.00446174044230484
SG2231	TcCLB.511229.50	0.00446174044230484
SG4439	TcCLB.506627.110	0.00454238194939383
SG5096	TcCLB.508823.20	0.00459449303743446
SG1477	TcCLB.511307.20	0.00465129863643342
SG2702	TcCLB.510993.10	0.00465129863643342
SG8233	TcCLB.511003.30	0.00465129863643342
SG3250	TcCLB.511815.40	0.00470884447353068
SG3018	TcCLB.506195.150	0.00473383536264033
SG2356	TcCLB.509683.20	0.00478028332723772
SG4926	TcCLB.503617.31	0.00478028332723772
SG5052	TcCLB.506435.110	0.00478028332723772
SG3464	TcCLB.506963.70	0.00479426271292232
SG4190	TcCLB.509835.30	0.00479426271292232
SG8863	TcCLB.506321.280	0.00479426271292232
SG5985	TcCLB.507023.180	0.00480845197138674
SG6067	TcCLB.508817.140	0.00482798167927889
SG3080	TcCLB.509859.40	0.00483417637890296
SG2654	TcCLB.506583.30	0.00484897631590435
SG7747	TcCLB.508827.110	0.00484897631590435
SG8638	TcCLB.511367.199	0.00493822966467415
SG3591	TcCLB.511903.134	0.00494645797248594
SG2200	TcCLB.510615.10	0.00495079437545899
SG5520	TcCLB.507491.70	0.00504827888260521
SG3467	TcCLB.503617.10	0.00508075692062969
SG6803	TcCLB.510879.180	0.00508760218941109

Supra gene	Representative ID	FDR
SG1526	TcCLB.506885.300	0.00510428130952481
SG7159	TcCLB.510743.10	0.00514456015616567
SG2973	TcCLB.511517.160	0.00515688636279232
SG1898	TcCLB.507817.80	0.00520078932421305
SG4903	TcCLB.511253.20	0.00527337972990147
SG1239	TcCLB.508637.150	0.00530620392773876
SG6931	TcCLB.510761.30	0.00530620392773876
SG8510	TcCLB.511283.240	0.00530620392773876
SG1452	TcCLB.509599.100	0.00534163878136394
SG3814	TcCLB.508153.680	0.00534163878136394
SG2976	TcCLB.508689.20	0.00540245050165069
SG1104	TcCLB.507715.20	0.00550799163070301
SG0640	TcCLB.507951.299	0.00552083995374304
SG8767	TcCLB.510187.380	0.00556821205209992
SG1317	TcCLB.511217.120	0.0055869326830733
SG3711	TcCLB.511021.90	0.0055869326830733
SG7599	TcCLB.511211.70	0.00562386670968057
SG6937	TcCLB.445635.10	0.00564567302893335
SG4885	TcCLB.511361.10	0.00564857133635784
SG1380	TcCLB.507021.50	0.00565973807855172
SG5374	TcCLB.506735.40	0.00565973807855172
SG5391	TcCLB.504013.55	0.00565973807855172
SG0419	TcCLB.508153.1050	0.00571001485436844
SG8573	TcCLB.507053.20	0.00573244948701495
SG5216	TcCLB.503679.30	0.00573510045770737
SG3028	TcCLB.511821.110	0.00574437756042132
SG2492	TcCLB.506797.110	0.00574981511741126
SG4789	TcCLB.511289.90	0.005816159890649
SG3739	TcCLB.506975.63	0.00603981372237957
SG3518	TcCLB.511511.150	0.0060735013049542
SG2465	TcCLB.503465.10	0.00610904932297616
SG2826	TcCLB.509717.30	0.00610904932297616
SG5750	TcCLB.510735.90	0.00610904932297616
SG8213	TcCLB.508547.190	0.00614972442501329
SG7329	TcCLB.504147.110	0.00616402582021793
SG2169	TcCLB.507049.60	0.0062160537481741 5
SG6986	TcCLB.507641.50	0.0062173810372491 8
SG4951	TcCLB.503579.40	0.00629010384546653
SG7011	TcCLB.506795.70	0.00631029839497067
SG6907	TcCLB.510431.140	0.00634096754376919
SG0584	TcCLB.509921.51	0.00634703533013692
SG2926	TcCLB.511649.100	0.00634703533013692
SG2992	TcCLB.510421.140	0.0063938417347681 7
SG4991	TcCLB.507515.120	0.00648420136778516
SG4038	TcCLB.507853.10	0.00649772120890841
SG2509	TcCLB.509755.50	0.00655171702529622
SG3654	TcCLB.510301.30	0.00663168178543718
SG8145	TcCLB.509003.70	0.00663168178543718
SG6188	TcCLB.509179.130	0.00669956845421798
SG1861	TcCLB.509937.160	0.0067091699536991
SG1671	TcCLB.509943.30	0.00672762645191971
SG8498	TcCLB.511807.190	0.00672762645191971
SG2974	TcCLB.509937.50	0.00674337171717076

Supra gene	Representative ID	FDR
SG3831	TcCLB.506553.10	0.00674337171717076
SG2181	TcCLB.424123.40	0.00678404480241282
SG1410	TcCLB.510101.480	0.00681820323602793
SG4857	TcCLB.507047.140	0.00682349081866941
SG6093	TcCLB.508661.10	0.00682349081866941
SG2085	TcCLB.505071.90	0.00683380904184168
SG6804	TcCLB.506885.60	0.00689397399446478
SG7108	TcCLB.506945.200	0.00689397399446478
SG7752	TcCLB.508173.264	0.00689601260787331
SG8268	TcCLB.511809.10	0.00692376443370777
SG8425	TcCLB.507019.30	0.00692376443370777
SG4656	TcCLB.503891.120	0.00694207842120474
SG4670	TcCLB.511389.110	0.00699008349719516
SG2980	TcCLB.508817.110	0.00699388183018912
SG4209	TcCLB.508741.170	0.00699388183018912
SG0321	TcCLB.508499.39	0.00707727515072815
SG5730	TcCLB.511249.110	0.00712478155119456
SG8149	TcCLB.508823.60	0.00718613034230559
SG3185	TcCLB.509777.60	0.00719138338947696
SG5174	TcCLB.511729.60	0.00723798237022746
SG4106	TcCLB.503747.40	0.00725027054435666
SG3765	TcCLB.508707.160	0.00727190902437799
SG6507	TcCLB.506275.20	0.00736672951400456
SG4146	TcCLB.508153.270	0.00736985451846769
SG4199	TcCLB.510655.130	0.00736985451846769
SG4735	TcCLB.508263.30	0.00736985451846769
SG7028	TcCLB.509703.10	0.00736985451846769
SG7545	TcCLB.506925.14	0.00736985451846769
SG1612	TcCLB.508153.1110	0.00743429607082211
SG2783	TcCLB.507519.150	0.00747225349582734
SG8304	TcCLB.504071.40	0.00748605691723676
SG6939	TcCLB.506925.310	0.00768914923268969
SG0622	TcCLB.506871.20	0.00770003790536571
SG8421	TcCLB.509527.120	0.00770312338000088
SG7340	TcCLB.506625.190	0.00771465231615007
SG1020	TcCLB.508625.160	0.00790723812388328
SG3607	TcCLB.510443.30	0.00790723812388328
SG7008	TcCLB.511215.50	0.00820722844902689
SG8589	TcCLB.510821.60	0.00820722844902689
SG4157	TcCLB.504073.20	0.00827929151799913
SG6336	TcCLB.509799.60	0.00836026897313866
SG5789	TcCLB.467287.10	0.00840347745458332
SG6019	TcCLB.511001.70	0.00842020306773793
SG0763	TcCLB.507715.10	0.00842687407998195
SG8880	TcCLB.511725.250	0.00842687407998195
SG1386	TcCLB.506959.70	0.00842727848178976
SG1690	TcCLB.509023.140	0.00847654147515437
SG0150	TcCLB.506279.140	0.00862194632010625
SG2537	TcCLB.509607.70	0.00866055813142103
SG3589	TcCLB.510143.120	0.00867530258268852
SG8155	TcCLB.510187.480	0.00867530258268852
SG8075	TcCLB.508461.350	0.00876767241447679
SG4236	TcCLB.509267.40	0.00877318450155812

Supra gene	Representative ID	FDR
SG4713	TcCLB.507711.90	0.00880813175356978
SG1501	TcCLB.509429.160	0.00886147651180398
SG2305	TcCLB.509207.80	0.00891619682462909
SG2543	TcCLB.510661.244	0.00894505314575407
SG4049	TcCLB.511909.70	0.00894505314575407
SG4334	TcCLB.507857.10	0.00894505314575407
SG7068	TcCLB.509267.20	0.00894505314575407
SG1566	TcCLB.508443.30	0.00896476130070729
SG2463	TcCLB.508857.140	0.00906529480629163
SG6618	TcCLB.511649.150	0.00906529480629163
SG0920	TcCLB.509063.30	0.00909763535620134
SG7914	TcCLB.508547.50	0.00909763535620134
SG2193	TcCLB.503449.14	0.00920586946093193
SG8628	TcCLB.510799.60	0.00922602174678888
SG5291	TcCLB.508943.20	0.00924086478380251
SG7136	TcCLB.507105.50	0.00930387825562981
SG0222	TcCLB.510065.10	0.00934116888527908
SG6658	TcCLB.507019.83	0.00937087935873574
SG4653	TcCLB.506789.270	0.00944877308135807
SG1327	TcCLB.508851.200	0.00949241040177579
SG3782	TcCLB.511289.50	0.00955217663940607
SG1426	TcCLB.507093.40	0.00958894592122689
SG7866	TcCLB.506175.120	0.00976625682828751
SG3923	TcCLB.510517.60	0.00985509598720211
SG0038	TcCLB.511835.10	0.00991188758704778
SG2902	TcCLB.508153.1100	0.00991188758704778
SG5617	TcCLB.503995.50	0.00996788780475544
SG8275	TcCLB.510901.230	0.00996788780475544
SG3802	TcCLB.507705.10	0.0100537638572497
SG2396	TcCLB.509799.140	0.0100944386398465
SG7616	TcCLB.506933.80	0.0101432641539439
SG7016	TcCLB.506375.80	0.0102639571787231
SG8644	TcCLB.507583.40	0.0103774627383922
SG2404	TcCLB.508989.30	0.0103779408779423
SG7483	TcCLB.507091.160	0.0103995461621624
SG1991	TcCLB.508445.30	0.0104187161460053
SG7837	TcCLB.511283.200	0.0104187161460053
SG3142	TcCLB.510611.10	0.01043019367739
SG2201	TcCLB.504175.40	0.01043566410038
SG3698	TcCLB.510889.231	0.01043566410038
SG1269	TcCLB.509693.70	0.0104718473701957
SG6347	TcCLB.508677.134	0.0105289021513
SG1940	TcCLB.508153.240	0.0105381674012578
SG7827	TcCLB.506679.70	0.0105381674012578
SG8948	TcCLB.506445.110	0.0105381674012578
SG4219	TcCLB.506855.20	0.0105595528659168
SG8557	TcCLB.511389.40	0.0105595528659168
SG5972	TcCLB.510121.40	0.010612291422841
SG5241	TcCLB.510065.30	0.0106521973572098
SG4409	TcCLB.510001.20	0.0106700606464166
SG3928	TcCLB.511725.40	0.0106884166185018
SG3002	TcCLB.505171.60	0.0107538150351749
SG5106	TcCLB.506989.10	0.0109083768989892

Supra gene	Representative ID	FDR
SG7751	TcCLB.508153.140	0.0109874756031175
SG3681	TcCLB.503703.30	0.0109992375101375
SG8232	TcCLB.508879.40	0.0110186877418106
SG6479	TcCLB.508909.140	0.0110829795927671
SG3895	TcCLB.503823.120	0.0111138967660774
SG5406	TcCLB.506297.10	0.0111563936551314
SG6498	TcCLB.506925.390	0.0111755826143644
SG3239	TcCLB.506729.90	0.0111893551786396
SG1635	TcCLB.507003.60	0.0111938150192259
SG1371	TcCLB.510149.40	0.0112646766197253
SG1323	TcCLB.509025.20	0.0113443041186157
SG8580	TcCLB.508799.10	0.0113512226082253
SG3281	TcCLB.507775.40	0.0114314637989198
SG2348	TcCLB.504105.180	0.0114405538823094
SG1354	TcCLB.509747.50	0.0115292476927784
SG5341	TcCLB.508351.20	0.0115361546241239
SG3005	TcCLB.508693.90	0.0115635805966661
SG1033	TcCLB.504051.20	0.0116400751242107
SG1280	TcCLB.509571.40	0.0116400751242107
SG1214	TcCLB.503551.20	0.0117174229373201
SG7131	TcCLB.503467.20	0.0118000805628252
SG3297	TcCLB.506819.30	0.0118177178731577
SG4323	TcCLB.508707.320	0.0118201888296632
SG8637	TcCLB.511277.400	0.0118201888296632
SG4739	TcCLB.510965.10	0.0121494988171864
SG8147	TcCLB.508461.110	0.0121494988171864
SG6869	TcCLB.510599.70	0.01219923593953
SG7031	TcCLB.508647.90	0.0122487733775885
SG1456	TcCLB.510647.60	0.0122509683356977
SG3546	TcCLB.510421.210	0.0122934655522127
SG2766	TcCLB.507889.10	0.0124419978371332
SG3774	TcCLB.509153.60	0.0125700782773959
SG6579	TcCLB.508211.50	0.0125824497148831
SG1766	TcCLB.510105.20	0.0126155838614398
SG5744	TcCLB.511545.170	0.0127132943764059
SG8696	TcCLB.511511.6	0.0127132943764059
SG8042	TcCLB.508479.340	0.0127715007449016
SG1273	TcCLB.508027.70	0.0129270647133462
SG0207	TcCLB.511607.60	0.012934569001313
SG8966	TcCLB.510187.100	0.012934569001313
SG2452	TcCLB.511291.50	0.0129554555348106
SG5436	TcCLB.508357.90	0.0129554555348106
SG1856	TcCLB.503819.20	0.0129924670424914
SG3743	TcCLB.511817.10	0.0129924670424914
SG4373	TcCLB.511871.90	0.0129924670424914
SG5003	TcCLB.503527.40	0.0129924670424914
SG5891	TcCLB.511479.39	0.0130875420493326
SG6970	TcCLB.504153.240	0.0130996718238535
SG7317	TcCLB.506925.440	0.0131046187003517
SG7886	TcCLB.511725.170	0.0131046187003517
SG3479	TcCLB.507897.30	0.0131445247365801
SG4955	TcCLB.503411.10	0.0133264649710885
SG8284	TcCLB.508461.370	0.0134449912108173

Supra gene	Representative ID	FDR
SG5153	TcCLB.511237.110	0.0134790471740958
SG7075	TcCLB.506777.24	0.0134932360587262
SG6547	TcCLB.511285.70	0.0135432540635592
SG5363	TcCLB.508707.227	0.0135756572528024
SG2673	TcCLB.503829.70	0.0136163090021424
SG1495	TcCLB.509065.36	0.0136330021937897
SG8365	TcCLB.509525.20	0.0136383195150938
SG3582	TcCLB.503975.20	0.0137409063798318
SG4478	TcCLB.511621.170	0.0137663257577539
SG1179	TcCLB.508365.100	0.0138983710501869
SG4041	TcCLB.510667.50	0.0139777324513699
SG2017	TcCLB.511661.40	0.0140094633449627
SG6054	TcCLB.511001.80	0.0140094633449627
SG0768	TcCLB.506247.370	0.0140337585491362
SG5261	TcCLB.509795.40	0.0141565616736738
SG6150	TcCLB.509671.189	0.0141772247480775
SG5509	TcCLB.510579.50	0.0143471285465274
SG5545	TcCLB.511903.40	0.0143630186776
SG4973	TcCLB.510533.80	0.0143965376354581
SG7966	TcCLB.508693.190	0.0145004866230932
SG1275	TcCLB.511245.120	0.0145386171458223
SG5766	TcCLB.506869.10	0.0146963992127522
SG1254	TcCLB.506733.80	0.0149938778665969
SG3420	TcCLB.511425.40	0.015103726480572
SG6103	TcCLB.511719.30	0.0151045038117956
SG5467	TcCLB.511837.10	0.0151619895383731
SG2564	TcCLB.509171.47	0.0151827020986033
SG3396	TcCLB.509599.120	0.0152150591459619
SG8440	TcCLB.506821.200	0.0152718618856278
SG2954	TcCLB.511301.44	0.0155841042713501
SG0369	TcCLB.503781.90	0.015641220420164
SG6243	TcCLB.507793.20	0.0157367053092096
SG6264	TcCLB.511717.20	0.0158434065657907
SG2933	TcCLB.508699.80	0.015914471095467
SG6384	TcCLB.509023.150	0.0159874744043494
SG7979	TcCLB.510901.30	0.0160074281273622
SG1369	TcCLB.509875.260	0.0160579488715677
SG1458	TcCLB.511233.90	0.0161240450160249
SG6961	TcCLB.511867.130	0.0161288470461797
SG3536	TcCLB.504157.70	0.0161444506840337
SG2943	TcCLB.508641.100	0.0161445666531068
SG3126	TcCLB.503917.14	0.0161580130728016
SG5667	TcCLB.509607.60	0.0161580130728016
SG7420	TcCLB.511365.80	0.0162711972122875
SG4361	TcCLB.509331.74	0.0162720798767558
SG8822	TcCLB.511727.210	0.0162720798767558
SG8957	TcCLB.511367.220	0.0162720798767558
SG7749	TcCLB.510329.90	0.0162958404399839
SG6276	TcCLB.503885.100	0.0163599603583715
SG0735	TcCLB.511467.16	0.0163747121657976
SG3834	TcCLB.511039.6	0.0164075606155364
SG8614	TcCLB.511003.60	0.0164287407487974
SG5260	TcCLB.511555.20	0.016470248188358

Supra gene	Representative ID	FDR
SG6538	TcCLB.506717.10	0.0165705303259132
SG3292	TcCLB.511239.110	0.0165925909853025
SG3926	TcCLB.427789.20	0.0166420510398754
SG7708	TcCLB.510187.160	0.0166420510398754
SG0196	TcCLB.506243.135	0.0166683962891292
SG0797	TcCLB.507083.10	0.0166683962891292
SG0942	TcCLB.506743.4	0.0167024118397182
SG5191	TcCLB.508713.30	0.0167024118397182
SG7601	TcCLB.507093.250	0.0167024118397182
SG6621	TcCLB.508905.10	0.0167643290776433
SG1046	TcCLB.511583.20	0.0168902380816196
SG4428	TcCLB.507087.80	0.0169564340544408
SG5874	TcCLB.511903.190	0.0170467581449776
SG0109	TcCLB.508789.120	0.0171640175991482
SG6453	TcCLB.405737.14	0.0172092356817284
SG3950	TcCLB.503795.10	0.0174024502681666
SG0303	TcCLB.510899.59	0.0175431385856854
SG1819	TcCLB.509319.90	0.0175431385856854
SG5050	TcCLB.511517.150	0.0176079312441052
SG3491	TcCLB.511445.100	0.0176286559967406
SG2568	TcCLB.506855.180	0.017664205231287
SG5252	TcCLB.506411.30	0.017664205231287
SG5869	TcCLB.507011.180	0.0177263295501741
SG7792	TcCLB.510901.220	0.0178839572353435
SG8594	TcCLB.510945.70	0.0179554132312786
SG5876	TcCLB.508387.150	0.0179955374078364
SG0190	TcCLB.506885.210	0.0179992270471385
SG7652	TcCLB.510901.180	0.0179992270471385
SG5237	TcCLB.509719.40	0.0180557249498397
SG7976	TcCLB.508459.18	0.01807050357331
SG8262	TcCLB.507769.30	0.0180840406217993
SG0496	TcCLB.509007.99	0.0181632726024193
SG0701	TcCLB.509935.20	0.0185652139574262
SG6308	TcCLB.509911.90	0.0187043164681364
SG3738	TcCLB.507787.70	0.0189336236619408
SG7722	TcCLB.506679.60	0.0189336236619408
SG3855	TcCLB.507601.20	0.0189413536575742
SG7957	TcCLB.508533.40	0.0189413536575742
SG2077	TcCLB.511649.94	0.0190067569693766
SG4873	TcCLB.507941.70	0.0190067569693766
SG7845	TcCLB.510661.80	0.0190067569693766
SG5149	TcCLB.508503.70	0.0190826679662529
SG6769	TcCLB.511211.60	0.0190906637844012
SG3385	TcCLB.504797.90	0.0194410579408273
SG2950	TcCLB.510609.100	0.0195357684985414
SG3647	TcCLB.506475.90	0.0195357684985414
SG6377	TcCLB.509599.180	0.0196248306782312
SG1809	TcCLB.504167.14	0.0196279699658826
SG0454	TcCLB.509109.130	0.019798214108985
SG0631	TcCLB.506947.110	0.019798214108985
SG2409	TcCLB.506303.160	0.019798214108985
SG6681	TcCLB.509867.70	0.019798214108985
SG8480	TcCLB.510329.210	0.019798214108985

Supra gene	Representative ID	FDR
SG0111	TcCLB.508229.60	0.0198495711023353
SG0967	TcCLB.509059.20	0.0200535895991371
SG8497	TcCLB.510329.110	0.0200628969550756
SG4487	TcCLB.510339.74	0.0207601096248647
SG6890	TcCLB.506529.595	0.0208042474379214
SG2875	TcCLB.510595.40	0.0208760526557211
SG7944	TcCLB.504155.20	0.021034855474229
SG3795	TcCLB.504839.60	0.0213696072175835
SG6616	TcCLB.506229.60	0.02141107868904З
SG8162	TcCLB.506619.30	0.0215969116302946
SG0290	TcCLB.509943.20	0.0216235123092439
SG2134	TcCLB.503693.20	0.0216235123092439
SG1557	TcCLB.506679.270	0.0216555529729678
SG5668	TcCLB.510101.59	0.0216555529729678
SG6490	TcCLB.511151.100	0.0216555529729678
SG4524	TcCLB.506503.4	0.0217573909806658
SG3661	TcCLB.511657.40	0.0217760075516606
SG3929	TcCLB.510667.100	0.0217760075516606
SG1201	TcCLB.511825.210	0.0218099248739463
SG4917	TcCLB.507775.10	0.0218099248739463
SG5186	TcCLB.509895.50	0.0218099248739463
SG5979	TcCLB.506435.150	0.0218675514840454
SG4376	TcCLB.509999.20	0.0219845804228958
SG8545	TcCLB.510187.400	0.0219845804228958
SG6315	TcCLB.507715.30	0.0219866512314691
SG8829	TcCLB.507007.76	0.0220721159878026
SG1411	TcCLB.509611.20	0.0224331082596754
SG8431	TcCLB.506337.220	0.0224617964828214
SG4481	TcCLB.507001.110	0.0224908179967848
SG6417	TcCLB.506321.340	0.0224941432095165
SG2762	TcCLB.507787.20	0.0225439568346615
SG4392	TcCLB.506573.30	0.0226773504229188
SG5816	TcCLB.503897.70	0.0226773504229188
SG5958	TcCLB.505183.124	0.0226773504229188
SG6945	TcCLB.511421.60	0.0226773504229188
SG2198	TcCLB.503943.20	0.0226833417476424
SG5648	TcCLB.507143.80	0.0226833417476424
SG0001	TcCLB.503739.20	0.0227364589272275
SG7597	TcCLB.506625.230	0.0227595170967593
SG1054	TcCLB.507011.120	0.0227618559392545
SG6842	TcCLB.504147.70	0.0228421591226067
SG3017	TcCLB.506195.160	0.0230355499382782
SG1748	TcCLB.511907.30	0.0230514706242017
SG6399	TcCLB.507765.120	0.0236989641799094
SG2023	TcCLB.507895.140	0.0237744848060026
SG3879	TcCLB.511759.30	0.0238328796638802
SG5961	TcCLB.509461.60	0.0238328796638802
SG6651	TcCLB.510669.40	0.0238328796638802
SG5559	TcCLB.505193.20	0.0238804475042116
SG7920	TcCLB.511755.28	0.0238804475042116
SG3586	TcCLB.508153.590	0.0240517414059443
SG4961	TcCLB.507775.50	0.0241314759547494
SG3355	TcCLB.510303.70	0.0242103687400558

Supra gene	Representative ID	FDR
SG7479	TcCLB.508357.100	0.0244972730311467
SG5625	TcCLB.506681.50	0.0247026024131917
SG8950	TcCLB.508711.10	0.0248034731173192
SG4545	TcCLB.506605.174	0.0250566093517216
SG8173	TcCLB.506337.180	0.0250566093517216
SG6209	TcCLB.511383.30	0.025076673534679
SG1695	TcCLB.506201.150	0.0251221148414412
SG0237	TcCLB.504839.28	0.0254337421361061
SG6149	TcCLB.511153.120	0.0256011496607576
SG5263	TcCLB.511459.60	0.0256812284190132
SG6665	TcCLB.510949.10	0.0256812284190132
SG7808	TcCLB.511491.140	0.0256812284190132
SG2604	TcCLB.508577.160	0.0257236464588096
SG5882	TcCLB.511285.30	0.0257236464588096
SG6539	TcCLB.509623.10	0.0257236464588096
SG6434	TcCLB.508865.30	0.0259745506958255
SG0973	TcCLB.510353.10	0.0261220887365485
SG4815	TcCLB.510903.60	0.0262788116683942
SG4848	TcCLB.503791.20	0.0263125485069913
SG2256	TcCLB.506575.70	0.0263796510059561
SG3166	TcCLB.504153.340	0.0266889611211165
SG0772	TcCLB.506583.60	0.0269171660118465
SG4087	TcCLB.503981.80	0.0269171660118465
SG3503	TcCLB.506479.80	0.0270506980606363
SG4429	TcCLB.504207.10	0.0270506980606363
SG7588	TcCLB.508727.50	0.0270506980606363
SG2490	TcCLB.507467.119	0.0272537876654948
SG5248	TcCLB.508851.180	0.0273606803335067
SG0862	TcCLB.508153.60	0.027364920434326
SG7958	TcCLB.503461.20	0.027364920434326
SG0686	TcCLB.508881.70	0.0274191748623479
SG7460	TcCLB.511313.30	0.0274780858036908
SG4371	TcCLB.503733.10	0.0274941303167605
SG5013	TcCLB.508777.130	0.0274941303167605
SG5787	TcCLB.508175.370	0.0279112791150051
SG0180	TcCLB.510693.270	0.0281427173725544
SG3918	TcCLB.507073.20	0.0281427173725544
SG8378	TcCLB.506247.220	0.0281427173725544
SG7767	TcCLB.507019.20	0.0282920943562924
SG7743	TcCLB.506855.190	0.0283560875642668
SG1450	TcCLB.511649.160	0.0284088822343352
SG1146	TcCLB.510565.20	0.028409460126132
SG1514	TcCLB.511717.80	0.0284430044754466
SG2294	TcCLB.506851.30	0.0284887468641966
SG4722	TcCLB.510659.240	0.0284957534815041
SG5632	TcCLB.506401.100	0.0284957534815041
SG2227	TcCLB.509999.110	0.0287462790988036
SG2602	TcCLB.511761.40	0.0287633323415761
SG3314	TcCLB.510027.20	0.0287633323415761
SG2217	TcCLB.511751.30	0.0289066511663494
SG4421	TcCLB.511825.260	0.0292329685734744
SG1611	TcCLB.510657.90	0.0293160052461423
SG8960	TcCLB.509545.40	0.0293160052461423

Supra gene	Representative ID	FDR
SG1621	TcCLB.511821.40	0.0293743415674341
SG1259	TcCLB.508075.50	0.0295825726351954
SG3331	TcCLB.504021.60	0.0295825726351954
SG5305	TcCLB.508857.130	0.0295877170545233
SG4331	TcCLB.504157.100	0.029644477034502
SG2984	TcCLB.506691.40	0.0296570585421956
SG5061	TcCLB.510725.40	0.0296721963492361
SG4216	TcCLB.510967.20	0.02989616205276
SG3621	TcCLB.511629.30	0.0300684770048006
SG3722	TcCLB.510431.250	0.0302486127502509
SG6364	TcCLB.507031.170	0.0303943390913184
SG8601	TcCLB.506559.410	0.0303943390913184
SG2390	TcCLB.506337.20	0.0304934762826962
SG6077	TcCLB.510729.160	0.0305487828217862
SG4162	TcCLB.505193.84	0.0306377290262278
SG2508	TcCLB.509525.100	0.0306868844483788
SG7831	TcCLB.506247.355	0.0306868844483788
SG4414	TcCLB.511277.20	0.0307666854841237
SG1007	TcCLB.508901.10	0.0310531478605797
SG0213	TcCLB.506961.25	0.0310788324147586
SG3481	TcCLB.507521.130	0.0311046479391891
SG7936	TcCLB.509997.20	0.0311356214617397
SG4841	TcCLB.506257.60	0.0311415888655803
SG6151	TcCLB.511621.60	0.0311415888655803
SG5257	TcCLB.511825.220	0.0311417958486902
SG2323	TcCLB.506681.24	0.0311683545499905
SG0753	TcCLB.507623.60	0.0314591776195082
SG0986	TcCLB.506585.40	0.0315783595847988
SG5926	TcCLB.504001.10	0.0316287261402436
SG1804	TcCLB.510967.10	0.0317281840963935
SG4762	TcCLB.506725.20	0.0317281840963935
SG1974	TcCLB.508347.20	0.0319540506661013
SG3683	TcCLB.511621.190	0.0320406909132676
SG6458	TcCLB.503939.10	0.0321245360900039
SG2784	TcCLB.510665.40	0.0322000536107321
SG3414	TcCLB.506195.120	0.0322000536107321
SG3576	TcCLB.504427.20	0.0322000536107321
SG0755	TcCLB.510003.30	0.0322052607333781
SG4241	TcCLB.506989.50	0.0323632436213926
SG6744	TcCLB.510879.130	0.0323632436213926
SG6432	TcCLB.508865.20	0.0324639555702719
SG7279	TcCLB.503747.30	0.0325356045674126
SG3641	TcCLB.510329.320	0.0325815049315679
SG5745	TcCLB.510897.70	0.0328950679380515
SG7283	TcCLB.506289.80	0.0329152264528271
SG5115	TcCLB.504045.120	0.0330294713572251
SG0632	TcCLB.503571.19	0.0330810836677541
SG5748	TcCLB.508543.60	0.0330810836677541
SG8339	TcCLB.510363.310	0.0335827304982813
SG3756	TcCLB.506295.80	0.0336560886320746
SG4809	TcCLB.508045.30	0.0338411592315464
SG4657	TcCLB.510259.24	0.0342104250926194
SG2960	TcCLB.503939.84	0.0342119999141072

Supra gene	Representative ID	FDR
SG3340	TcCLB.511153.144	0.0342119999141072
SG8542	TcCLB.508479.174	0.034287276933987
SG0568	TcCLB.509353.20	0.0344005770374146
SG4691	TcCLB.508269.20	0.0344005770374146
SG5859	TcCLB.511737.10	0.0344005770374146
SG3380	TcCLB.503823.30	0.0346594449907417
SG1021	TcCLB.509261.30	0.0346806909800175
SG1037	TcCLB.508981.39	0.0346806909800175
SG1172	TcCLB.511545.100	0.0348071216226455
SG5015	TcCLB.510691.10	0.0348071216226455
SG7265	TcCLB.509247.50	0.0348071216226455
SG8382	TcCLB.509995.20	0.0349288934112128
SG6128	TcCLB.508503.60	0.0350695156584551
SG7133	TcCLB.511873.10	0.035093912041084
SG5553	TcCLB.511603.500	0.035112645644461
SG8097	TcCLB.508533.50	0.035112645644461
SG8206	TcCLB.511295.20	0.035112645644461
SG2042	TcCLB.507765.110	0.0352657757796705
SG2372	TcCLB.507035.120	0.0352657757796705
SG6386	TcCLB.506753.110	0.0352657757796705
SG8821	TcCLB.511277.359	0.0352657757796705
SG0861	TcCLB.505071.110	0.0354851050111099
SG1149	TcCLB.505193.10	0.0356336824564014
SG6086	TcCLB.482319.19	0.0356953502088877
SG0896	TcCLB.503835.10	0.0358889718424593
SG0200	TcCLB.510187.420	0.0359277589039433
SG0393	TcCLB.504075.10	0.0359526212406063
SG2072	TcCLB.503945.20	0.036293122731363
SG2860	TcCLB.508231.70	0.0363036310291158
SG4827	TcCLB.508153.810	0.0363379183842643
SG2587	TcCLB.511261.10	0.036367008706249
SG2755	TcCLB.508153.730	0.0365280066751186
SG6488	TcCLB.510729.240	0.0365280066751186
SG5489	TcCLB.508909.214	0.0365662804930409
SG8203	TcCLB.509647.90	0.0365662804930409
SG6470	TcCLB.509775.9	0.0366130199603214
SG2493	TcCLB.511907.60	0.0366507398360815
SG3268	TcCLB.460127.20	0.0366938511093348
SG8759	TcCLB.507063.10	0.0369716434142889
SG5540	TcCLB.506883.50	0.0370012447845173
SG3860	TcCLB.508893.10	0.0372166283719633
SG3334	TcCLB.507023.110	0.037257181914037
SG3835	TcCLB.508265.70	0.0373200493593071
SG3991	TcCLB.511807.40	0.0373647468567356
SG5495	TcCLB.508799.50	0.0373868387630145
SG6411	TcCLB.506177.30	0.0373868387630145
SG3064	TcCLB.509791.90	0.0374865419982864
SG7307	TcCLB.504149.100	0.0375285061444641
SG2655	TcCLB.510257.60	0.03755366493049
SG0872	TcCLB.506855.160	0.0376445816396623
SG1274	TcCLB.504213.90	0.0376983563037049
SG6118	TcCLB.511545.90	0.0378768112978287
SG1509	TcCLB.511361.70	0.0379559717792212

Supra gene	Representative ID	FDR
SG3293	TcCLB.506053.20	0.0379559717792212
SG5533	TcCLB.510899.19	0.0382134936900715
SG8857	TcCLB.510817.60	0.0385180817134615
SG5940	TcCLB.509027.70	0.0386972528039116
SG6655	TcCLB.511185.10	0.0387538069627678
SG0595	TcCLB.504575.40	0.0389701372255616
SG4582	TcCLB.510519.70	0.0389701372255616
SG6037	TcCLB.507951.160	0.0390710806055403
SG2624	TcCLB.510533.90	0.0391624740322804
SG6137	TcCLB.508989.110	0.0394546274166853
SG2468	TcCLB.508859.100	0.0394725712904878
SG3221	TcCLB.508693.80	0.0394725712904878
SG3906	TcCLB.509047.40	0.0394725712904878
SG3979	TcCLB.510339.90	0.0394725712904878
SG4774	TcCLB.506629.30	0.0394782607216958
SG2731	TcCLB.504253.20	0.039724714877575
SG7880	TcCLB.511003.130	0.039724714877575
SG8175	TcCLB.510713.90	0.039764758751356
SG3124	TcCLB.506681.40	0.0400380172181985
SG1970	TcCLB.507081.20	0.0404024486952608
SG3389	TcCLB.506789.160	0.0404024486952608
SG2844	TcCLB.510889.340	0.040433245379459
SG0464	TcCLB.510339.50	0.0404389278039382
SG4568	TcCLB.511281.20	0.0404411507506733
SG7923	TcCLB.510187.120	0.0404649808443724
SG6162	TcCLB.511287.20	0.0405559425148824
SG7221	TcCLB.504557.40	0.0405559425148824
SG3823	TcCLB.510243.70	0.0409162555118501
SG3970	TcCLB.511529.170	0.0409162555118501
SG0343	TcCLB.457101.20	0.0410259338406147
SG4333	TcCLB.511863.20	0.0410422554342971
SG4342	TcCLB.506477.40	0.0410422554342971
SG0211	TcCLB.509197.10	0.041313297888569
SG6013	TcCLB.509965.190	0.0413813014438254
SG3176	TcCLB.511051.10	0.0413965425780877
SG3878	TcCLB.506151.30	0.0415460902821763
SG8513	TcCLB.509217.90	0.0415460902821763
SG1469	TcCLB.506297.190	0.0421234808673212
SG7715	TcCLB.511255.10	0.0421443039126296
SG7269	TcCLB.509583.19	0.0421749731909652
SG3363	TcCLB.504575.15	0.0422383434347604
SG7129	TcCLB.511215.110	0.0422383434347604
SG7246	TcCLB.506625.210	0.0422383434347604
SG6390	TcCLB.508911.30	0.0424829263543739
SG3699	TcCLB.510885.30	0.0424871534783405
SG8711	TcCLB.508817.130	0.0424871534783405
SG8667	TcCLB.511277.390	0.042912764355125
SG0663	TcCLB.503453.20	0.0429838552699992
SG8564	TcCLB.506583.4	0.0433098631034678
SG2266	TcCLB.508637.140	0.0434971326379826
SG2944	TcCLB.511867.80	0.043535152670073
SG4928	TcCLB.508593.40	0.0435436263805516
SG8288	TcCLB.508823.100	0.0436428878943503

Supra gene	Representative ID	FDR
SG1714	TcCLB.511245.20	0.0436913283535816
SG2206	TcCLB.506789.240	0.0437633302794056
SG8170	TcCLB.508827.50	0.0438208734142459
SG1348	TcCLB.508799.160	0.0439159152789787
SG7697	TcCLB.509999.80	0.043920798938238
SG2330	TcCLB.507609.30	0.0439398777368969
SG0993	TcCLB.507395.10	0.043959067254302
SG1194	TcCLB.506821.20	0.043959067254302
SG1927	TcCLB.510309.10	0.043959067254302
SG6254	TcCLB.504021.20	0.0441208569891137
SG2073	TcCLB.504137.40	0.044266808922451
SG3073	TcCLB.511239.70	0.044266808922451
SG0371	TcCLB.511337.10	0.0445109313628994
SG8495	TcCLB.504045.90	0.0445787621818405
SG3695	TcCLB.503939.30	0.0446069153531924
SG6800	TcCLB.510073.24	0.0446069153531924
SG7021	TcCLB.506501.110	0.0449451213503008
SG7700	TcCLB.510329.180	0.0449549334886646
SG3452	TcCLB.510729.80	0.0450042010705674
SG6133	TcCLB.507675.30	0.045035834264983
SG3274	TcCLB.508881.20	0.04517652165631
SG4206	TcCLB.504867.90	0.0453575749859937
SG3357	TcCLB.506363.140	0.0454271837547829
SG3401	TcCLB.506147.120	0.0454271837547829
SG8379	TcCLB.506247.330	0.0454404846218838
SG1034	TcCLB.508707.30	0.0454557515276075
SG2507	TcCLB.509755.10	0.0454557515276075
SG3422	TcCLB.509747.40	0.0457071612810093
SG2884	TcCLB.509047.14	0.0460226210110367
SG3339	TcCLB.506779.60	0.046063120788702
SG4345	TcCLB.506529.590	0.0460843241960591
SG0542	TcCLB.507711.60	0.0462078624954704
SG6117	TcCLB.508411.10	0.0463932420999465
SG1788	TcCLB.510731.20	0.0464047283437793
SG4245	TcCLB.503505.14	0.0464047283437793
SG4211	TcCLB.507317.30	0.0464197975643623
SG0461	TcCLB.511715.100	0.0465562248769442
SG2335	TcCLB.508737.187	0.046590626894939
SG4942	TcCLB.510045.30	0.046590626894939
SG4565	TcCLB.511295.50	0.0467002671926222
SG1613	TcCLB.511583.40	0.0467276501485576
SG5989	TcCLB.508919.70	0.046928024405811
SG4024	TcCLB.507029.10	0.0469858175154036
SG6632	TcCLB.507681.20	0.0471006391618186
SG0528	TcCLB.508411.40	0.0472712500881956
SG6474	TcCLB.506959.40	0.0472712500881956
SG8765	TcCLB.506679.120	0.0474605213762422
SG2062	TcCLB.509455.20	0.0476270761217261
SG0733	TcCLB.508153.370	0.0476876176013922
SG0761	TcCLB.506223.120	0.0476876176013922
SG1818	TcCLB.506779.110	0.0476876176013922
SG3723	TcCLB.507515.10	0.0476876176013922
SG4799	TcCLB.507073.49	0.0476876176013922

Supra gene	Representative ID	FDR
SG6169	TcCLB.510889.290	0.0476876176013922
SG7938	TcCLB.507069.120	0.0476876176013922
SG3284	TcCLB.507031.140	0.0479260512755389
SG4688	TcCLB.510421.200	0.0480523089813491
SG2391	TcCLB.507787.80	0.0480774357365509
SG3112	TcCLB.506459.280	0.0482001284789143
SG1363	TcCLB.508965.60	0.0483397144613407
SG5356	TcCLB.508135.10	0.0484196267872194
SG3808	TcCLB.506303.30	0.0484847218464798
SG4775	TcCLB.510265.10	0.048577447167535
SG6422	TcCLB.511461.60	0.0487677050655319
SG1222	TcCLB.511003.210	0.0490025419219358
SG2274	TcCLB.508441.20	0.0490865508359646
SG5481	TcCLB.504103.100	0.0491884615169351
SG3598	TcCLB.507787.40	0.0493172316541762
SG5612	TcCLB.511479.20	0.0494684347246492
SG2301	TcCLB.506779.90	0.0496356110755553
SG1080	TcCLB.506009.10	0.0497311422103978
SG6549	TcCLB.510889.80	0.0497585720854841
SG1453	TcCLB.510359.220	0.0498077959265358
SG8074	TcCLB.510187.234	0.0498693355141242
SG0450	TcCLB.509123.10	0.0500807453997024
SG8598	TcCLB.508823.130	0.0500807453997024
SG6742	TcCLB.508771.55	0.0501474412535114
SG4472	TcCLB.508821.30	0.0501652864302961
SG7702	TcCLB.506855.240	0.050653072300091
SG5715	TcCLB.510329.220	0.0506878847163705
SG3343	TcCLB.511483.10	0.0508551085015576
SG5657	TcCLB.509499.38	0.0509465839448089
SG4476	TcCLB.506303.80	0.050984846871435
SG3099	TcCLB.511817.210	0.051281273766446
SG0908	TcCLB.510145.10	0.0513499877623172
SG1599	TcCLB.510191.40	0.0515765642705693
SG6951	TcCLB.506925.460	0.0517264704321596
SG6852	TcCLB.511867.110	0.051792232460469
SG6613	TcCLB.510121.20	0.0518375943868954
SG8410	TcCLB.510419.20	0.0518983777927075
SG6599	TcCLB.511817.20	0.0519206035907128
SG7595	TcCLB.509699.110	0.0519522068882168
SG5865	TcCLB.509177.50	0.0520328252721572
SG7169	TcCLB.504153.70	0.0520328252721572
SG4577	TcCLB.510357.20	0.0521497771665042
SG1096	TcCLB.509965.350	0.0521796896021011
SG3318	TcCLB.511277.554	0.0522677801868011
SG6420	TcCLB.506839.34	0.0523439807489839
SG1772	TcCLB.508015.40	0.0524893297519605
SG4114	TcCLB.511245.79	0.0525303068345575
SG4549	TcCLB.511755.50	0.0528064032319547
SG2718	TcCLB.504797.10	0.052809927405007
SG3044	TcCLB.511439.60	0.0528604748829258
SG6535	TcCLB.508569.90	0.0529386734734323
SG0650	TcCLB.506181.70	0.0530163653322485
SG2824	TcCLB.509005.60	0.0530163653322485

Supra gene	Representative ID	FDR
SG1055	TcCLB.506181.20	0.0530675319256549
SG8357	TcCLB.506559.380	0.0533345409820853
SG5206	TcCLB.508153.800	0.0535354715759467
SG7024	TcCLB.504153.190	0.0535902180494879
SG4536	TcCLB.506319.70	0.0540590411081178
SG2563	TcCLB.508277.310	0.0542977772924047
SG1525	TcCLB.509119.30	0.0543762447149829
SG3510	TcCLB.507159.30	0.0544436008521921
SG4431	TcCLB.510031.20	0.0544436008521921
SG0322	TcCLB.511061.20	0.0546691487176959
SG3730	TcCLB.511907.210	0.0546691487176959
SG4627	TcCLB.506559.70	0.0549354170768622
SG1072	TcCLB.511309.20	0.0551977853317594
SG1253	TcCLB.504827.80	0.0552700605187831
SG7167	TcCLB.509769.36	0.0553261235153156
SG0691	TcCLB.508215.9	0.0553910585961348
SG6295	TcCLB.506755.180	0.0553910585961348
SG2913	TcCLB.508349.30	0.0554972098290631
SG3373	TcCLB.510763.60	0.0558553706636914
SG5918	TcCLB.506629.50	0.0561811345154106
SG5508	TcCLB.504111.10	0.0561906263078858
SG8550	TcCLB.504105.40	0.0562428504505753
SG6454	TcCLB.511229.70	0.0563753756092139
SG8380	TcCLB.504109.160	0.0563753756092139
SG4411	TcCLB.506691.70	0.0565057842447591
SG4137	TcCLB.510753.30	0.0565536632442374
SG0024	TcCLB.404711.10	0.0565544209622738
SG0679	TcCLB.510943.50	0.056645168509797
SG7732	TcCLB.511633.70	0.0568116112713591
SG2879	TcCLB.511661.90	0.0568184369015285
SG8818	TcCLB.506559.400	0.0568184369015285
SG4567	TcCLB.508851.154	0.0569903472462506
SG1417	TcCLB.510299.70	0.0570853734368371
SG1673	TcCLB.506201.39	0.0571412158126751
SG2392	TcCLB.507081.110	0.0572711013779238
SG0612	TcCLB.507627.9	0.0573583001477155
SG2433	TcCLB.508707.224	0.0574822256877855
SG2586	TcCLB.508741.330	0.0574822256877855
SG4367	TcCLB.503733.20	0.0575535043293896
SG4601	TcCLB.506979.30	0.0576036242324921
SG0521	TcCLB.455721.9	0.0577166337539089
SG5433	TcCLB.458759.14	0.0578182248853246
SG5524	TcCLB.507825.40	0.0578182248853246
SG4092	TcCLB.511285.100	0.0579197540279227
SG6009	TcCLB.509717.40	0.0580534081383594
SG3789	TcCLB.510887.50	0.0580934506924959
SG7551	TcCLB.507641.170	0.0580934506924959
SG1758	TcCLB.504149.175	0.0582270378463485
SG2733	TcCLB.506435.50	0.0582270378463485
SG4144	TcCLB.503415.40	0.0583184801036434
SG4819	TcCLB.507801.110	0.0583289400656182
SG5253	TcCLB.508613.50	0.0584028869943701
SG5560	TcCLB.510299.20	0.0586700841277552

Supra gene	Representative ID	FDR
SG6047	TcCLB.510123.60	0.0588364627068812
SG1297	TcCLB.506529.260	0.0589083598626172
SG4818	TcCLB.509671.99	0.0589083598626172
SG0501	TcCLB.510203.49	0.0589607498810889
SG3639	TcCLB.511907.50	0.0595780121927051
SG3229	TcCLB.509893.20	0.0597295748373882
SG5577	TcCLB.509067.60	0.0597943284061327
SG5403	TcCLB.508707.10	0.060303818930766
SG5119	TcCLB.503847.30	0.0603665801972592
SG5933	TcCLB.510889.221	0.0607347208943947
SG1834	TcCLB.510691.30	0.0607932848810072
SG3215	TcCLB.510889.251	0.0607932848810072
SG5000	TcCLB.506975.69	0.0607932848810072
SG5827	TcCLB.508545.20	0.0607932848810072
SG6952	TcCLB.506925.260	0.0607932848810072
SG4201	TcCLB.508809.50	0.0609075125878102
SG7997	TcCLB.508153.150	0.0609623912356355
SG1765	TcCLB.511287.110	0.0613543379088923
SG4284	TcCLB.509551.60	0.0619306798546065
SG6481	TcCLB.511385.60	0.062183216863134З
SG6783	TcCLB.511603.70	0.0621977092760956
SG2777	TcCLB.511671.80	0.0622122086679597
SG8303	TcCLB.504045.50	0.0622122086679597
SG2859	TcCLB.508231.220	0.0624323864742793
SG3423	TcCLB.508269.30	0.0629334400657069
SG8643	TcCLB.510087.90	0.0629334400657069
SG4796	TcCLB.508637.114	0.0630667711200911
SG7612	TcCLB.509589.50	0.063359890454194
SG5493	TcCLB.510661.190	0.0640379305905344
SG2088	TcCLB.508241.100	0.0642565461942685
SG0284	TcCLB.511807.100	0.0643198796763957
SG4445	TcCLB.506275.50	0.0645471342834234
SG3426	TcCLB.503809.120	0.0645990094564336
SG2436	TcCLB.511717.110	0.0647573589433367
SG5279	TcCLB.510863.20	0.0647573589433367
SG7913	TcCLB.504109.180	0.0649351866189085
SG6332	TcCLB.506009.30	0.0649518326397335
SG8116	TcCLB.511727.40	0.064961301174451
SG6173	TcCLB.511815.60	0.0650290900937231
SG7477	TcCLB.506925.64	0.0652207912255726
SG0987	TcCLB.507031.20	0.0654891730582357
SG3367	TcCLB.508577.150	0.0654891730582357
SG7046	TcCLB.504153.150	0.0655190173616166
SG4224	TcCLB.509065.120	0.0655247540419203
SG2135	TcCLB.511269.50	0.0656353409965858
SG7818	TcCLB.509527.30	0.0656484491858236
SG3627	TcCLB.506925.360	0.0661231084250799
SG5482	TcCLB.511511.70	0.0663507621157201
SG5652	TcCLB.506435.390	0.0663507621157201
SG7846	TcCLB.508461.270	0.0663507621157201
SG2620	TcCLB.510421.320	0.0664633458579124
SG3244	TcCLB.509105.4	0.0664633458579124
SG1800	TcCLB.505037.50	0.0664823774725415

Supra gene	Representative ID	FDR
SG8734	TcCLB.508897.70	0.0664823774725415
SG4349	TcCLB.503581.30	0.066485866068248
SG8134	TcCLB.506425.90	0.0665369211238544
SG5737	TcCLB.510173.110	0.0668225713786635
SG4269	TcCLB.511017.50	0.0668432289316217
SG8467	TcCLB.506247.340	0.0668432289316217
SG1444	TcCLB.503697.40	0.0668466636826739
SG2599	TcCLB.506247.30	0.0668466636826739
SG8962	TcCLB.506475.130	0.0668466636826739
SG2761	TcCLB.503939.40	0.0672186380338135
SG5231	TcCLB.508543.100	0.0674127509339835
SG4200	TcCLB.507519.30	0.0675053279929353
SG6684	TcCLB.504081.420	0.0677800912658754
SG1442	TcCLB.511499.50	0.0677809399402998
SG3058	TcCLB.510575.120	0.0678977670045203
SG6552	TcCLB.511247.9	0.0678977670045203
SG0685	TcCLB.503899.119	0.0679675130343318
SG0603	TcCLB.509647.190	0.0680806723880972
SG6619	TcCLB.508153.994	0.0681164085005452
SG8065	TcCLB.506883.100	0.0681570906031252
SG8017	TcCLB.508479.230	0.0682391071544555
SG2071	TcCLB.508661.40	0.0682479723225914
SG4625	TcCLB.510227.20	0.0683220354768368
SG6461	TcCLB.511827.64	0.0684014459555014
SG2480	TcCLB.503987.60	0.0687023257876851
SG1125	TcCLB.506789.290	0.0688122660327232
SG0132	TcCLB.507633.80	0.0690173162391881
SG4454	TcCLB.506573.70	0.0690173162391881
SG4477	TcCLB.506979.20	0.0690173162391881
SG8473	TcCLB.507053.90	0.069074400717208
SG8652	TcCLB.511809.20	0.069074400717208
SG6820	TcCLB.503905.30	0.0692315553555577
SG6845	TcCLB.511421.50	0.0696363126742015
SG1615	TcCLB.511761.10	0.0697009149053877
SG0099	TcCLB.510583.74	0.0697011860267773
SG0447	TcCLB.510441.41	0.0697011860267773
SG2868	TcCLB.510575.20	0.0697011860267773
SG5774	TcCLB.510053.50	0.0697011860267773
SG2849	TcCLB.511759.40	0.0699790758582585
SG4626	TcCLB.509049.20	0.0699790758582585
SG5574	TcCLB.509569.50	0.0699790758582585
SG4871	TcCLB.509875.170	0.0702498866647948
SG4651	TcCLB.507011.110	0.0704213295654311
SG2155	TcCLB.511511.50	0.0704390752424477
SG6857	TcCLB.503425.20	0.0704730820547731
SG1711	TcCLB.509991.9	0.070474292625287
SG0139	TcCLB.508437.20	0.0708951743631492
SG4537	TcCLB.507017.40	0.07095397257445
SG5417	TcCLB.507081.50	0.0711468115987829
SG1431	TcCLB.507771.30	0.0713087117823586
SG7344	TcCLB.507747.130	0.0713969045423485
SG5702	TcCLB.506287.140	0.071433744785076
SG6394	TcCLB.507041.120	0.0715190187149118

Supra gene	Representative ID	FDR
SG0068	TcCLB.508221.350	0.0716374570958416
SG2811	TcCLB.506303.90	0.0716374570958416
SG5422	TcCLB.509211.30	0.0716374570958416
SG5480	TcCLB.509033.50	0.0718713931026021
SG6429	TcCLB.503679.10	0.0719016568336223
SG4097	TcCLB.511685.60	0.0720725662571293
SG1184	TcCLB.503525.20	0.0721906300465092
SG2883	TcCLB.511131.89	0.0721906300465092
SG8808	TcCLB.508689.30	0.0721906300465092
SG4417	TcCLB.503939.110	0.0722263206474852
SG2831	TcCLB.506841.8	0.0722633967296315
SG6586	TcCLB.508593.140	0.0722633967296315
SG1627	TcCLB.509331.80	0.072384807148986
SG5449	TcCLB.507669.190	0.072384807148986
SG3674	TcCLB.510599.30	0.0725940473610002
SG3180	TcCLB.506529.250	0.0726113330581769
SG5396	TcCLB.507539.40	0.0726417998869345
SG0874	TcCLB.511301.100	0.0727960630320004
SG4616	TcCLB.507317.34	0.0730177384772314
SG3763	TcCLB.511717.180	0.0731937152344542
SG5399	TcCLB.511385.40	0.0731937152344542
SG8026	TcCLB.506279.10	0.07329852342971
SG8405	TcCLB.504105.100	0.0735224371165888
SG8911	TcCLB.509061.10	0.0737743882952996
SG2961	TcCLB.511233.100	0.0738787228328136
SG3519	TcCLB.509055.44	0.0738787228328136
SG5063	TcCLB.511165.80	0.0741889777736724
SG7877	TcCLB.504123.10	0.0742098964419448
SG0096	TcCLB.453767.20	0.0742203006277466
SG4975	TcCLB.508257.104	0.074278724125403
SG1513	TcCLB.503913.40	0.0743306318202195
SG1532	TcCLB.508741.290	0.0744624442686605
SG6582	TcCLB.505999.90	0.0744624442686605
SG8395	TcCLB.508257.120	0.0744624442686605
SG8412	TcCLB.511277.410	0.0744624442686605
SG5929	TcCLB.507831.70	0.0744882183084542
SG8274	TcCLB.510901.200	0.0749343523983445
SG2797	TcCLB.506959.94	0.0749380332199075
SG6638	TcCLB.508461.310	0.0749380332199075
SG7610	TcCLB.507039.41	0.0749380332199075
SG2529	TcCLB.507663.80	0.0750057950255213
SG5101	TcCLB.508355.20	0.0755935356390494
SG1487	TcCLB.509157.120	0.07579274448177
SG5045	TcCLB.503541.10	0.0771167364332646
SG8238	TcCLB.511755.80	0.0779607816441421
SG4899	TcCLB.506753.230	0.0781828108416116
SG4480	TcCLB.507001.50	0.0784666962989707
SG8650	TcCLB.510363.250	0.0784666962989707
SG2291	TcCLB.511127.50	0.0784870062674475
SG7930	TcCLB.511491.20	0.0788298362585787
SG7686	TcCLB.511807.256	0.078873025222089
SG1592	TcCLB.510525.130	0.078887864738084
SG6192	TcCLB.503749.30	0.0788937275262295

Supra gene	Representative ID	FDR
SG3191	TcCLB.509007.90	0.0789855457165849
SG5342	TcCLB.508501.300	0.0792992300018244
SG4862	TcCLB.505071.30	0.0796409538424907
SG2260	TcCLB.509027.50	0.0797195204040909
SG7100	TcCLB.508163.250	0.0798097585945882
SG1307	TcCLB.504741.180	0.0804088861113591
SG7987	TcCLB.508461.140	0.0805090685074584
SG2078	TcCLB.511741.10	0.0805639614689151
SG7070	TcCLB.508647.140	0.0805639614689151
SG8181	TcCLB.511725.100	0.0805934670423466
SG1565	TcCLB.506821.100	0.0807086395010236
SG7859	TcCLB.504071.20	0.0808133935534235
SG8689	TcCLB.511277.310	0.0808425846511021
SG1061	TcCLB.503865.70	0.0809141499149659
SG4448	TcCLB.508891.60	0.0809141499149659
SG1752	TcCLB.507519.154	0.0810300237720687
SG4941	TcCLB.510359.20	0.0810337104306546
SG0636	TcCLB.503643.20	0.0812322134217005
SG4435	TcCLB.508153.840	0.0812322134217005
SG4093	TcCLB.503837.20	0.0812603954712271
SG4995	TcCLB.508153.340	0.0813052077329376
SG7071	TcCLB.509233.140	0.0820273802743799
SG4155	TcCLB.508637.63	0.0820800648910175
SG8225	TcCLB.508897.40	0.0821806174354821
SG3279	TcCLB.510943.23	0.0822168092443454
SG4232	TcCLB.507849.70	0.0822168092443454
SG4440	TcCLB.510969.40	0.0822168092443454
SG6457	TcCLB.511857.70	0.0822168092443454
SG7584	TcCLB.506625.60	0.0822168092443454
SG5394	TcCLB.506559.59	0.0822997600365899
SG7684	TcCLB.511295.30	0.0822997600365899
SG3905	TcCLB.504069.34	0.0824497437017642
SG8653	TcCLB.509733.100	0.0824497437017642
SG4134	TcCLB.503703.10	0.0824502024625818
SG2523	TcCLB.510611.70	0.0824551945588785
SG3472	TcCLB.509637.27	0.0824551945588785
SG5397	TcCLB.509105.80	0.0824551945588785
SG5964	TcCLB.510155.90	0.0824551945588785
SG8216	TcCLB.508409.140	0.0825263706940327
SG8701	TcCLB.510901.100	0.0827459963365546
SG4259	TcCLB.504079.10	0.0828678005596385
SG6653	TcCLB.508871.40	0.0828712278676422
SG2175	TcCLB.511735.70	0.0828844834851452
SG5234	TcCLB.503557.40	0.0829040219061219
SG2222	TcCLB.507519.160	0.0832341344649712
SG3852	TcCLB.511867.20	0.0832341344649712
SG5001	TcCLB.508501.230	0.0832341344649712
SG6132	TcCLB.510355.230	0.0832341344649712
SG4099	TcCLB.510889.50	0.0832597580664928
SG5408	TcCLB.508507.50	0.0833069345081504
SG8921	TcCLB.506627.20	0.0833069345081504
SG3052	TcCLB.510661.270	0.0833449598365014
SG2126	TcCLB.509611.50	0.0834703765458583

Supra gene	Representative ID	FDR
SG6696	TcCLB.508693.10	0.0834703765458583
SG8167	TcCLB.504109.70	0.0834703765458583
SG0783	TcCLB.506973.120	0.0834803287948011
SG3646	TcCLB.511277.560	0.0834803287948011
SG6503	TcCLB.509287.60	0.0835406820987464
SG2880	TcCLB.504069.80	0.0838215304438707
SG4415	TcCLB.504045.114	0.0841138489399462
SG8770	TcCLB.506337.160	0.0841227055027862
SG8687	TcCLB.506559.454	0.0841850032137526
SG1433	TcCLB.511577.188	0.0842591874717663
SG1474	TcCLB.504741.20	0.0843484399967441
SG2475	TcCLB.509767.44	0.084448732962577
SG7239	TcCLB.508813.70	0.0844751658614029
SG0123	TcCLB.506011.10	0.0851146303549555
SG0518	TcCLB.511833.10	0.0851315809084655
SG2959	TcCLB.511287.100	0.085261773055103
SG7421	TcCLB.511215.30	0.085261773055103
SG3429	TcCLB.503925.60	0.0857214721365524
SG8265	TcCLB.510693.120	0.0857366147889045
SG4407	TcCLB.510911.30	0.0859126144423664
SG8519	TcCLB.511725.280	0.0859253914616514
SG2512	TcCLB.511249.10	0.0863648392743642
SG8038	TcCLB.506279.60	0.0863648392743642
SG7961	TcCLB.504123.40	0.086373264779343
SG7666	TcCLB.508409.40	0.0865526181146178
SG7754	TcCLB.508479.310	0.0866520588512349
SG3559	TcCLB.506581.20	0.0868098915006203
SG3133	TcCLB.506915.10	0.0868812861541381
SG1928	TcCLB.507795.100	0.0870906919201407
SG2611	TcCLB.511625.74	0.0870906919201407
SG5141	TcCLB.506303.50	0.0870906919201407
SG6250	TcCLB.507049.170	0.0870906919201407
SG8322	TcCLB.511367.280	0.0870906919201407
SG8491	TcCLB.507063.290	0.0870906919201407
SG0580	TcCLB.504215.30	0.087101059721794
SG0151	TcCLB.506267.39	0.087181445093298
SG4020	TcCLB.503513.10	0.087181445093298
SG4166	TcCLB.511277.70	0.087181445093298
SG5097	TcCLB.506815.40	0.087181445093298
SG7141	TcCLB.508307.134	0.087181445093298
SG8885	TcCLB.506335.69	0.0872462833663134
SG6624	TcCLB.506955.240	0.0872745377490636
SG3690	TcCLB.508405.110	0.0874555979107065
SG5847	TcCLB.506575.20	0.0874576746769488
SG1153	TcCLB.507737.30	0.0876580360806523
SG1660	TcCLB.509607.40	0.0876580360806523
SG8392	TcCLB.508461.400	0.0876580360806523
SG8420	TcCLB.506155.70	0.0876580360806523
SG1507	TcCLB.511143.12	0.0877843146616868
SG8428	TcCLB.507053.40	0.0877843146616868
SG4346	TcCLB.511817.120	0.0884477947505305
SG2001	TcCLB.509671.130	0.0896396954821668
SG7861	TcCLB.508233.30	0.08973432851182

Supra gene	Representative ID	FDR
SG7177	TcCLB.504149.70	0.0898625696519495
SG1218	TcCLB.509007.20	0.0900706903123165
SG2544	TcCLB.510431.70	0.0902092406263655
SG8125	TcCLB.507681.60	0.0902406922316488
SG4647	TcCLB.508409.280	0.0902486086766382
SG4959	TcCLB.509233.230	0.0902486086766382
SG2228	TcCLB.511589.50	0.0902899414562521
SG3785	TcCLB.511269.40	0.0902899414562521
SG7275	TcCLB.509433.30	0.0903129640995301
SG5833	TcCLB.506697.10	0.0903322867929645
SG2031	TcCLB.506399.70	0.0905168214998266
SG3006	TcCLB.509105.50	0.09072222526849
SG3408	TcCLB.511389.150	0.09072222526849
SG6473	TcCLB.508871.150	0.09072222526849
SG0057	TcCLB.507069.160	0.0908595156897482
SG0652	TcCLB.508865.4	0.0911426082717763
SG8775	TcCLB.511837.60	0.0911676504315324
SG0474	TcCLB.508939.60	0.0911693297663588
SG1069	TcCLB.507165.10	0.0912898529900757
SG0328	TcCLB.507533.21	0.0914908283159619
SG1300	TcCLB.503635.89	0.0914908283159619
SG6122	TcCLB.511277.10	0.0916083985809293
SG1170	TcCLB.504033.180	0.0917886571551925
SG2939	TcCLB.508175.133	0.0917886571551925
SG7437	TcCLB.508647.130	0.0922903831769584
SG8350	TcCLB.510483.250	0.09238045865418
SG2786	TcCLB.503975.70	0.0925419925017157
SG6659	TcCLB.511003.200	0.0926051961324844
SG3276	TcCLB.510945.60	0.0926967246880749
SG6607	TcCLB.506885.200	0.0928027255393379
SG1756	TcCLB.510895.20	0.0928613595891275
SG2578	TcCLB.506883.40	0.0928613595891275
SG7409	TcCLB.504149.30	0.0928613595891275
SG4820	TcCLB.504153.310	0.0931748816743076
SG1394	TcCLB.507793.30	0.0931986849529638
SG1979	TcCLB.503979.10	0.0931986849529638
SG3412	TcCLB.506303.130	0.0931986849529638
SG8114	TcCLB.506489.20	0.0932039693898118
SG6912	TcCLB.507093.160	0.0932162044916094
SG5230	TcCLB.510665.60	0.0932283983234268
SG6480	TcCLB.508919.140	0.0932283983234268
SG8892	TcCLB.508815.160	0.0935228189491639
SG2263	TcCLB.507029.60	0.0936361306570026
SG3107	TcCLB.511515.20	0.0936361306570026
SG4192	TcCLB.503723.70	0.0936361306570026
SG5517	TcCLB.504161.30	0.09436417475739
SG6789	TcCLB.506767.110	0.09436417475739
SG2872	TcCLB.511287.80	0.0944777152629834
SG2874	TcCLB.508387.50	0.0945300998734151
SG0340	TcCLB.511825.200	0.0945861148835774
SG8120	TcCLB.508461.470	0.0948451782202051
SG5060	TcCLB.508443.20	0.0948835698749852
SG5707	TcCLB.504741.80	0.0950424399709621

Supra gene	Representative ID	FDR
SG3704	TcCLB.506327.40	0.0956922817534839
SG4369	TcCLB.511167.40	0.0956922817534839
SG1155	TcCLB.506201.110	0.0959233367952753
SG4031	TcCLB.508857.160	0.0959233367952753
SG5051	TcCLB.506459.250	0.0964152598894408
SG7839	TcCLB.507623.125	0.0967964732507089
SG8027	TcCLB.508737.10	0.096900949774961
SG4359	TcCLB.511389.114	0.0972888032866426
SG6433	TcCLB.507063.130	0.0977937967673051
SG2140	TcCLB.509805.70	0.097813409205511
SG3873	TcCLB.507609.20	0.097813409205511
SG5339	TcCLB.511431.90	0.097813409205511
SG2341	TcCLB.503999.20	0.0979034219602578
SG3386	TcCLB.506973.10	0.0979034219602578
SG3702	TcCLB.509695.140	0.0979034219602578
SG0264	TcCLB.508805.30	0.0979165123032869
SG4280	TcCLB.509611.89	0.0982231111391965
SG8753	TcCLB.482319.10	0.0987521315591127
SG4062	TcCLB.503929.30	0.0988174037328077
SG7059	TcCLB.508103.14	0.0990596059097071
SG4996	TcCLB.507257.90	0.0992444489377231
SG5911	TcCLB.508507.59	0.0992444489377231
SG3552	TcCLB.511301.120	0.0994236792693351

*: complete statistical data and reads per kilobase million (RPKM) values can be obtained directly from the corresponding author.

TABLE IV

List of differentially expressed genes (DEGs) with their respective false discovery rates (FDR) for polysomal/granular (P/G) RNA fraction

Supra gene	Representative ID	FDR
	FDR < 0.001	
	FDR < 0.01	
	FDR < 0.1	
SG0346	TcCLB.504223.20	1.10750041328772e-05
SG2855	TcCLB.511277.60	1.10750041328772e-05
SG4050	TcCLB.506193.60	1.10750041328772e-05
SG5814	TcCLB.511511.20	1.10750041328772e-05
SG6583	TcCLB.503925.80	1.13651105152301e-05
SG7998	TcCLB.511511.3	1.20678177166239e-05
SG6137	TcCLB.508989.110	1.24179671629177e-05
SG6103	TcCLB.511719.30	1.35544261626548e-12
SG4776	TcCLB.508153.620	1.52532207600721e-06
SG6597	TcCLB.509999.140	1.52532207600721e-06
SG8425	TcCLB.507019.30	1.52883980001481e-05
SG8683	TcCLB.506247.190	1.55340772823491e-07
SG5160	TcCLB.508625.130	1.72804203740115e-08
SG1448	TcCLB.503555.40	2.39055664232696e-05
SG2389	TcCLB.506211.250	2.67871942029032e-05
SG2142	TcCLB.509429.280	2.79468380993562e-07
SG7053	TcCLB.508813.60	2.93996734588475e-14

Supra gene	Representative ID	FDR
SG5858	TcCLB.504229.110	2.97240874183142e-05
SG7767	TcCLB.507019.20	2.97240874183142e-05
SG3037	TcCLB.503525.4	3.01142180988915e-05
SG1958	TcCLB.508399.10	3.07027719914146e-07
SG6879	TcCLB.506635.139	3.12602484975416e-07
SG3604	TcCLB.507023.50	3.51936767559477e-06
SG3861	TcCLB.510423.50	3.51936767559477e-06
SG5170	TcCLB.504069.90	3.51936767559477e-06
SG2870	TcCLB.510581.20	3.55310876829993e-05
SG4032	TcCLB.510877.170	3.58893691394966e-06
SG3019	TcCLB.506241.160	3.93697378998175e-05
SG3329	TcCLB.508307.180	3.93697378998175e-05
SG3769	TcCLB.509713.30	3.93697378998175e-05
SG6638	TcCLB.508461.310	3.93697378998175e-05
SG1355	TcCLB.503565.29	4.05673913424211e-05
SG6601	TcCLB.511633.79	4.28626032451508e-06
SG0661	TcCLB.458241.10	4.83879935766689e-05
SG3295	TcCLB.509161.10	4.83879935766689e-05
SG3246	TcCLB.511295.60	5.29083279844227e-05
SG3970	TcCLB.511529.170	5.29083279844227e-05
SG1080	TcCLB.506009.10	5.50480297210861e-06
SG7577	TcCLB.506553.20	5.55520529452781e-05
SG2828	TcCLB.504199.20	5.56396567478124e-06
SG4532	TcCLB.511825.240	5.56396567478124e-06
SG8807	TcCLB.508173.180	5.90885386624956e-05
SG4917	TcCLB.507775.10	6.00940273242952e-06
SG8050	TcCLB.508017.30	6.07533300141629e-05
SG4075	TcCLB.508547.140	6.11974268624481e-05
SG5493	TcCLB.510661.190	6.62784798587688e-06
SG1473	TcCLB.507801.114	6.7165588577419e-05
SG0088	TcCLB.510217.10	6.97261583645192e-11
SG0148	TcCLB.511325.40	7.10019577571621e-05
SG0908	TcCLB.510145.10	7.36790129553302e-05
SG8318	TcCLB.508799.240	7.42673323043136e-07
SG2019	TcCLB.509109.160	8.23118769999791e-05
SG7779	TcCLB.508173.40	8.38697874693883e-05
SG1276	TcCLB.509601.140	8.40120944611799e-07
SG5338	TcCLB.503653.60	9.32438418013464e-05
SG1642	TcCLB.505183.30	9.53035178497522e-07
SG4441	TcCLB.506263.40	0.000101672000861757
SG6890	TcCLB.506529.595	0.000101927818954658
SG2312	TcCLB.507851.70	0.000110579098358016
SG5555	TcCLB.511277.140	0.000110579098358016
SG8968	TcCLB.509105.90	0.000113590758905792
SG4884	TcCLB.511283.124	0.00011506364505726
SG7652	TcCLB.510901.180	0.000134291183881425
SG8088	TcCLB.509437.170	0.000135953833172997
SG2646	TcCLB.507951.70	0.000138058783603592
SG4422	TcCLB.507305.40	0.000161022151420083
SG6791	TcCLB.506945.240	0.000161022151420083
SG3684	TcCLB.511151.54	0.000161471728872203
SG4526	TcCLB.504087.10	0.000180120535158395
SG5235	TcCLB.508799.150	0.000213112956137256

Supra gene	Representative ID	FDR
SG5890	TcCLB.510045.20	0.000223469574679328
SG3321	TcCLB.509003.30	0.000223481280173487
SG3859	TcCLB.510397.10	0.000228363777285146
SG4263	TcCLB.509937.130	0.000228363777285146
SG6027	TcCLB.508543.120	0.000228363777285146
SG6912	TcCLB.507093.160	0.00024528555117438
SG0632	TcCLB.503571.19	0.000304621873706574
SG1043	TcCLB.509617.40	0.000307841251849156
SG1919	TcCLB.507143.60	0.000310332967547997
SG2986	TcCLB.506195.300	0.000310332967547997
SG6398	TcCLB.507003.50	0.000310332967547997
SG7958	TcCLB.503461.20	0.000310332967547997
SG1370	TcCLB.506195.90	0.00031672208174263
SG2316	TcCLB.506727.150	0.000323234481443093
SG8398	TcCLB.504071.110	0.000368052916249017
SG3244	TcCLB.509105.4	0.000380975987805373
SG6982	TcCLB.506925.190	0.000380975987805373
SG8162	TcCLB.506619.30	0.000380975987805373
SG7886	TcCLB.511725.170	0.000382543758950203
SG1113	TcCLB.503975.50	0.000392997421506699
SG0351	TcCLB.504797.140	0.000407220080139268
SG0081	TcCLB.504015.30	0.00040984211504992
SG5648	TcCLB.507143.80	0.00042375723332197
SG1407	TcCLB.510359.290	0.000430633428812675
SG6749	TcCLB.504117.50	0.000470844516899454
SG2198	TcCLB.503943.20	0.000472381413463858
SG5232	TcCLB.403875.10	0.000473276043662071
SG6869	TcCLB.510599.70	0.000489266256340875
SG7597	TcCLB.506625.230	0.000492481824290065
SG8730	TcCLB.509647.180	0.000499248690897103
SG3633	TcCLB.503559.70	0.000506790914315077
SG0712	TcCLB.506559.129	0.000508445172386109
SG1615	TcCLB.511761.10	0.000508445172386109
SG6907	TcCLB.510431.140	0.000535157305974853
SG6867	TcCLB.504153.260	0.000556799841894931
SG5744	TcCLB.511545.170	0.000576781608464952
SG2025	TcCLB.507515.90	0.000581491330789466
SG6659	TcCLB.511003.200	0.000581491330789466
SG4789	TcCLB.511289.90	0.00059472899877852
SG5192	TcCLB.508577.80	0.000598190593001404
SG0846	TcCLB.508065.70	0.000629515367148499
SG2623	TcCLB.506181.90	0.000629515367148499
SG3001	TcCLB.508257.70	0.000629515367148499
SG1007	TcCLB.508901.10	0.00065816728462526
SG2985	TcCLB.510149.140	0.00065816728462526
SG5658	TcCLB.509331.150	0.00065816728462526
SG0251	TcCLB.510581.9	0.000660461464549539
SG2489	TcCLB.509099.130	0.000676884600363304
SG2849	TcCLB.511759.40	0.000679449841650376
SG6049	TcCLB.511825.20	0.000679449841650376
SG6498	TcCLB.506925.390	0.000679449841650376
SG3838	TcCLB.507609.40	0.000683340767898757
SG4690	TcCLB.510421.310	0.000683340767898757

Supra gene	Representative ID	FDR
SG3895	TcCLB.503823.120	0.000700823319095703
SG3950	TcCLB.503795.10	0.000700823319095703
SG4955	TcCLB.503411.10	0.00071898785900524
SG1074	TcCLB.511899.40	0.000764899004694868
SG2363	TcCLB.504137.50	0.000764899004694868
SG6931	TcCLB.510761.30	0.000794888322882786
SG0763	TcCLB.507715.10	0.000835094461867382
SG6433	TcCLB.507063.130	0.000852723929513126
SG5721	TcCLB.507031.90	0.000864101302188373
SG5855	TcCLB.507927.80	0.00088290969126587
SG2347	TcCLB.506195.290	0.000886244727163664
SG4926	TcCLB.503617.31	0.000912220045496489
SG8686	TcCLB.508863.10	0.000912220045496489
SG1601	TcCLB.504003.70	0.000995985388506219
SG5432	TcCLB.509859.30	0.00100597760956817
SG8418	TcCLB.507053.209	0.00101159822894001
SG8657	TcCLB.508479.300	0.00102969638219555
SG2752	TcCLB.506399.80	0.00104877946238568
SG2422	TcCLB.508593.130	0.00105139739036954
SG3108	TcCLB.507017.130	0.00105139739036954
SG7448	TcCLB.506411.25	0.001061666588424
SG2257	TcCLB.507049.100	0.00107342268365207
SG7028	TcCLB.509703.10	0.00107342268365207
SG8691	TcCLB.510087.100	0.00107342268365207
SG8959	TcCLB.510329.70	0.00107342268365207
SG3061	TcCLB.511807.64	0.00111225388396956
SG4228	TcCLB.510829.10	0.00111981880288002
SG3396	TcCLB.509599.120	0.00113884451801221
SG2810	TcCLB.508669.10	0.00114013476296358
SG0380	TcCLB.511757.70	0.0011951053927782
SG3805	TcCLB.509179.80	0.00121443536964682
SG4548	TcCLB.509237.50	0.00121443536964682
SG5863	TcCLB.506229.10	0.00121882645003125
SG3542	TcCLB.510001.10	0.00128499470362046
SG3990	TcCLB.511827.100	0.00128499470362046
SG0145	TcCLB.511911.90	0.00131027484604458
SG4064	TcCLB.510691.70	0.00131027484604458
SG4079	TcCLB.508175.39	0.00131027484604458
SG4199	TcCLB.510655.130	0.00131027484604458
SG6440	TcCLB.507089.30	0.00131027484604458
SG3211	TcCLB.505999.70	0.00131427489132381
SG3583	TcCLB.508461.40	0.00132614337651378
SG2117	TcCLB.509563.10	0.00134676359161995
SG5864	TcCLB.503571.10	0.00134676359161995
SG3179	TcCLB.506491.10	0.00135110762489021
SG5583	TcCLB.507007.45	0.00135142948092339
SG2135	TcCLB.511269.50	0.00136526416404062
SG6698	TcCLB.507251.10	0.00136526416404062
SG0690	TcCLB.506375.100	0.00138589254824425
SG5486	TcCLB.511411.16	0.00140552050618104
SG5963	TcCLB.503939.20	0.00140552050618104
SG8580	TcCLB.508799.10	0.00140552050618104
SG3302	TcCLB.511151.20	0.00143522400729334

Supra gene	Representative ID	FDR
SG7979	TcCLB.510901.30	0.00145861624050855
SG3449	TcCLB.510861.70	0.00149620077214839
SG7307	TcCLB.504149.100	0.00149620077214839
SG1791	TcCLB.508177.100	0.00150752641829854
SG7726	TcCLB.510977.9	0.00154399355009667
SG7329	TcCLB.504147.110	0.00167566040883814
SG8191	TcCLB.511003.90	0.00168814279711866
SG8224	TcCLB.511367.210	0.00168814279711866
SG1533	TcCLB.509001.20	0.00178497171995446
SG7098	TcCLB.506925.10	0.00189236242001312
SG6906	TcCLB.505073.10	0.00195402573417577
SG5557	TcCLB.510421.270	0.00203892891281979
SG3292	TcCLB.511239.110	0.00205551629178188
SG5111	TcCLB.402863.9	0.00207182922289675
SG0364	TcCLB.511253.31	0.0020866507245293
SG2622	TcCLB.506257.70	0.00209097074047476
SG6303	TcCLB.510357.90	0.00209097074047476
SG0747	TcCLB.511303.40	0.00210842058465221
SG1858	TcCLB.509799.50	0.00213181406093882
SG4757	TcCLB.511761.60	0.00229987368477298
SG6274	TcCLB.511153.70	0.00229987368477298
SG6862	TcCLB.482097.20	0.00229987368477298
SG7589	TcCLB.503975.40	0.00243626162940998
SG8052	TcCLB.506679.80	0.00243626162940998
SG5882	TcCLB.511285.30	0.00244313507344728
SG5696	TcCLB.506265.110	0.00257436016978057
SG2077	TcCLB.511649.94	0.00259909866825824
SG1748	TcCLB.511907.30	0.00262212927890131
SG6217	TcCLB.503955.100	0.00262783537509038
SG4179	TcCLB.509791.60	0.00262968613915595
SG5075	TcCLB.504161.50	0.00263774790630674
SG1758	TcCLB.504149.175	0.00263938948462161
SG2525	TcCLB.506573.50	0.00270668301659663
SG4321	TcCLB.511529.160	0.00290257101879438
SG4038	TcCLB.507853.10	0.00299039787980622
SG8275	TcCLB.510901.230	0.00299039787980622
SG0306	TcCLB.506201.70	0.00300285189152815
SG8813	TcCLB.511019.80	0.00300285189152815
SG8020	TcCLB.510129.20	0.00303118861763846
SG8724	TcCLB.506679.20	0.00303118861763846
SG4987	TcCLB.511209.60	0.00305218332927078
SG2247	TcCLB.503735.40	0.00310809849304033
SG1386	TcCLB.506959.70	0.00313293605407417
SG5193	TcCLB.511017.25	0.00313293605407417
SG6470	TcCLB.509775.9	0.00313944940309094
SG4144	TcCLB.503415.40	0.00317485084792937
SG3489	TcCLB.506303.60	0.00317696406068103
SG1397	TcCLB.511509.30	0.00330577536392677
SG2176	TcCLB.509053.70	0.00330577536392677
SG3523	TcCLB.509109.30	0.00330577536392677
SG5403	TcCLB.508707.10	0.00339307863026164
SG5578	TcCLB.506025.40	0.00340906796932007
SG2862	TcCLB.508231.190	0.00341395413333401

Supra gene	Representative ID	FDR
SG3084	TcCLB.511529.50	0.00341395413333401
SG3002	TcCLB.505171.60	0.00342442890868981
SG0796	TcCLB.510609.30	0.00344626647607006
SG3681	TcCLB.503703.30	0.00344626647607006
SG5496	TcCLB.509453.20	0.00344626647607006
SG6564	TcCLB.511323.60	0.00344626647607006
SG7393	TcCLB.504423.15	0.00344626647607006
SG8474	TcCLB.504071.60	0.00344626647607006
SG6948	TcCLB.509007.50	0.00345761091321588
SG3333	TcCLB.507057.30	0.00348844360286932
SG8007	TcCLB.507681.200	0.00348844360286932
SG1165	TcCLB.506825.70	0.00350829383886074
SG5441	TcCLB.506305.29	0.0035184647317871 8
SG1082	TcCLB.509835.10	0.00355016199622606
SG0708	TcCLB.507009.30	0.00356090393417438
SG0452	TcCLB.503731.9	0.00361760725380546
SG2023	TcCLB.507895.140	0.00361760725380546
SG2917	TcCLB.508593.30	0.00361760725380546
SG5024	TcCLB.508577.130	0.00361760725380546
SG5655	TcCLB.508231.60	0.00361760725380546
SG6190	TcCLB.511211.210	0.00361760725380546
SG3564	TcCLB.509029.80	0.00370755800507088
SG6294	TcCLB.503599.10	0.00370755800507088
SG8391	TcCLB.506419.10	0.00371243627104671
SG2991	TcCLB.508269.40	0.00372924166743087
SG4394	TcCLB.511181.80	0.00372924166743087
SG0942	TcCLB.506743.4	0.00375449342500165
SG2868	TcCLB.510575.20	0.00375449342500165
SG3285	TcCLB.509003.40	0.00375449342500165
SG4059	TcCLB.504021.74	0.00375449342500165
SG4537	TcCLB.507017.40	0.00376078714623584
SG5520	TcCLB.507491.70	0.00378359319334705
SG5589	TcCLB.508409.199	0.00378359319334705
SG4035	TcCLB.504167.40	0.00380011482675013
SG4938	TcCLB.503431.90	0.00392026441719965
SG0392	TcCLB.509237.120	0.00402670431966592
SG1057	TcCLB.510007.50	0.00404631553185593
SG0281	TcCLB.509639.10	0.0041274648723236
SG7293	TcCLB.507641.140	0.00421310824213705
SG1634	TcCLB.506727.140	0.00429973446848572
SG2527	TcCLB.508661.70	0.00429973446848572
SG6243	TcCLB.507793.20	0.00429973446848572
SG6406	TcCLB.508153.520	0.00429973446848572
SG7738	TcCLB.511807.150	0.00429973446848572
SG8545	TcCLB.510187.400	0.00429973446848572
SG5016	TcCLB.509769.30	0.00436128248894577
SG3320	TcCLB.511355.30	0.00436920431227058
SG6160	TcCLB.510155.50	0.00440396326393541
SG1708	TcCLB.506477.60	0.00443723595829411
SG0620	TcCLB.509999.120	0.00447911210317561
SG6090	TcCLB.509229.10	0.00447911210317561
SG8739	TcCLB.506559.550	0.00449938180613716
SG0879	TcCLB.503531.40	0.004543544764048

Supra gene	Representative ID	FDR
SG5345	TcCLB.507317.20	0.00454726079444184
SG2519	TcCLB.508905.30	0.00457678322383681
SG2856	TcCLB.503521.60	0.00458827340610748
SG2704	TcCLB.509499.4	0.00464372664273476
SG2649	TcCLB.511353.69	0.0046856549590999
SG7368	TcCLB.503651.49	0.0046856549590999
SG6381	TcCLB.506841.20	0.00472764776012277
SG8822	TcCLB.511727.210	0.00477623418713767
SG4070	TcCLB.510823.30	0.0048180303059662
SG7049	TcCLB.511421.170	0.0048180303059662
SG2367	TcCLB.503595.10	0.00482035920650713
SG3667	TcCLB.504253.40	0.00486699997309501
SG4165	TcCLB.510335.10	0.00486699997309501
SG3464	TcCLB.506963.70	0.00487451970521112
SG3615	TcCLB.510219.30	0.00491038578278744
SG7739	TcCLB.511807.130	0.00491038578278744
SG3486	TcCLB.509671.160	0.00498452426253193
SG2451	TcCLB.503409.10	0.00511352273572552
SG6307	TcCLB.506229.64	0.00515869136385917
SG2767	TcCLB.507251.20	0.00516471315591967
SG2373	TcCLB.508355.114	0.00523261891585617
SG1789	TcCLB.509551.70	0.00527207689958938
SG8015	TcCLB.508461.240	0.00527225355080294
SG6070	TcCLB.506821.90	0.00533805947436663
SG8335	TcCLB.511317.70	0.00536886559398458
SG3647	TcCLB.506475.90	0.00536983874027681
SG4906	TcCLB.509463.10	0.00536983874027681
SG8934	TcCLB.506317.10	0.00536983874027681
SG7928	TcCLB.506627.100	0.00546025811794429
SG1197	TcCLB.507485.100	0.00546340300263475
SG1942	TcCLB.508153.260	0.00546340300263475
SG4651	TcCLB.507011.110	0.00546340300263475
SG7457	TcCLB.511439.70	0.00546340300263475
SG2642	TcCLB.506773.90	0.00548741789367537
SG8613	TcCLB.510303.200	0.00552092914656812
SG1008	TcCLB.509237.90	0.00564652382472502
SG5155	TcCLB.509683.80	0.00564652382472502
SG1575	TcCLB.508999.260	0.00569691390223995
SG8942	TcCLB.511511.5	0.00570628737701781
SG6530	TcCLB.506297.140	0.00574497811031863
SG3410	TcCLB.511025.80	0.00582334129907528
SG3284	TcCLB.507031.140	0.0058564307130863
SG5943	TcCLB.511653.20	0.0058564307130863
SG7223	TcCLB.441241.10	0.00587379433919448
SG0847	TcCLB.508823.10	0.00589440635745039
SG8847	TcCLB.511885.50	0.00594906865993843
SG3326	TcCLB.509717.34	0.00598437964706945
SG6961	TcCLB.511867.130	0.00610263070350341
SG3519	TcCLB.509055.44	0.00619690735378892
SG2229	TcCLB.510039.64	0.00655217339275243
SG2418	TcCLB.503999.100	0.00655217339275243
SG3689	TcCLB.509879.40	0.00655217339275243
SG3816	TcCLB.508741.340	0.00655217339275243

Supra gene	Representative ID	FDR
SG2923	TcCLB.508257.30	0.00660771272760154
SG3742	TcCLB.506529.30	0.00660771272760154
SG4452	TcCLB.506009.40	0.00660771272760154
SG5407	TcCLB.510657.20	0.00660771272760154
SG8516	TcCLB.510687.70	0.00660771272760154
SG2714	TcCLB.508409.160	0.00663993813554334
SG8306	TcCLB.508707.200	0.0066974009714628
SG0493	TcCLB.510663.19	0.0067228717758863
SG5213	TcCLB.509715.40	0.0067228717758863
SG6029	TcCLB.503715.40	0.00677829204627201
SG3882	TcCLB.506821.110	0.00678183670546501
SG7302	TcCLB.504147.240	0.00686303137773293
SG6492	TcCLB.511165.40	0.00695205941199676
SG6404	TcCLB.511903.90	0.00711866369099257
SG4150	TcCLB.510311.30	0.00720213138605702
SG0244	TcCLB.506989.300	0.00740393674166389
SG4168	TcCLB.504243.40	0.00740393674166389
SG2627	TcCLB.511127.180	0.00741941915162528
SG5284	TcCLB.506195.100	0.0075227803954942
SG6579	TcCLB.508211.50	0.00761922602863903
SG7815	TcCLB.508971.30	0.00763718781099131
SG4638	TcCLB.510659.270	0.00766333640761551
SG1099	TcCLB.506405.140	0.0076812936898448
SG1983	TcCLB.510655.80	0.0076812936898448
SG2873	TcCLB.509109.140	0.0076812936898448
SG3141	TcCLB.510039.80	0.0076812936898448
SG4556	TcCLB.506777.20	0.0076812936898448
SG5730	TcCLB.511249.110	0.0076812936898448
SG7195	TcCLB.504147.190	0.0076812936898448
SG8943	TcCLB.509907.60	0.0076812936898448
SG2786	TcCLB.503975.70	0.00775486849239432
SG2926	TcCLB.511649.100	0.00775486849239432
SG5604	TcCLB.511459.50	0.00775486849239432
SG4182	TcCLB.509499.10	0.00785027052785386
SG7173	TcCLB.503955.80	0.00785485797506339
SG5852	TcCLB.507677.150	0.00786675396086237
SG1334	TcCLB.509065.100	0.00787988997493156
SG1141	TcCLB.506811.50	0.00788706910678386
SG2181	TcCLB.424123.40	0.00788706910678386
SG2930	TcCLB.511481.70	0.00788706910678386
SG3272	TcCLB.510797.20	0.00788706910678386
SG3979	TcCLB.510339.90	0.00788706910678386
SG7827	TcCLB.506679.70	0.00788706910678386
SG8480	TcCLB.510329.210	0.00788706910678386
SG2699	TcCLB.506355.130	0.00789532396830965
SG0393	TcCLB.504075.10	0.00793215870001828
SG2277	TcCLB.511907.240	0.00797445127293252
SG5274	TcCLB.506775.180	0.0080088175459311
SG8323	TcCLB.503413.4	0.00801198908401736
SG4172	TcCLB.506327.80	0.00804329230253054
SG6911	TcCLB.508307.170	0.00805238002521131
SG1755	TcCLB.511903.45	0.0081019921318863
SG2453	TcCLB.504001.20	0.0081019921318863

Supra gene	Representative ID	FDR
SG3124	TcCLB.506681.40	0.00810199213188633
SG5015	TcCLB.510691.10	0.00810199213188633
SG6900	TcCLB.511545.80	0.00810199213188633
SG8206	TcCLB.511295.20	0.00824112055168625
SG0357	TcCLB.510603.100	0.00833578384634853
SG1155	TcCLB.506201.110	0.00833578384634853
SG2349	TcCLB.508405.90	0.00833578384634853
SG0823	TcCLB.506629.220	0.00835542271103017
SG5915	TcCLB.503975.100	0.00835542271103017
SG7593	TcCLB.508687.25	0.00835542271103017
SG7642	TcCLB.506977.60	0.00835542271103017
SG5508	TcCLB.504111.10	0.00842424498746108
SG7710	TcCLB.511389.80	0.00846133022324831
SG7760	TcCLB.506791.20	0.00850082120050536
SG3879	TcCLB.511759.30	0.00850573577271567
SG5714	TcCLB.506123.24	0.00850573577271567
SG6242	TcCLB.506503.60	0.00850573577271567
SG5154	TcCLB.511847.50	0.00857347711246439
SG6743	TcCLB.509137.10	0.00863448732778556
SG2325	TcCLB.509539.44	0.00864042054893699
SG5063	TcCLB.511165.80	0.00864042054893699
SG7626	TcCLB.503419.50	0.00864042054893699
SG8124	TcCLB.511003.160	0.00864042054893699
SG2392	TcCLB.507081.110	0.00865451883510225
SG4814	TcCLB.506821.80	0.00867177106749544
SG7394	TcCLB.507099.30	0.00867177106749544
SG6230	TcCLB.510729.140	0.00881981434523794
SG3926	TcCLB.427789.20	0.00889792347581794
SG2097	TcCLB.503939.50	0.00899491825924898
SG3947	TcCLB.507031.29	0.00899491825924898
SG6439	TcCLB.508273.70	0.00899491825924898
SG6759	TcCLB.503955.89	0.0091541484749842
SG2226	TcCLB.504125.100	0.00917776227726235
SG3064	TcCLB.509791.90	0.00917776227726235
SG8890	TcCLB.508871.140	0.00918562865425542
SG3934	TcCLB.506825.10	0.00924608094552624
SG3827	TcCLB.507049.20	0.00925775677486853
SG5789	TcCLB.467287.10	0.00927169020334931
SG6254	TcCLB.504021.20	0.00931726458400247
SG0441	TcCLB.505999.10	0.00932115937923609
SG5717	TcCLB.505997.200	0.00938443433138375
SG5211	TcCLB.510257.24	0.00939628381242698
SG0084	TcCLB.506551.10	0.00940376110785879
SG0304	TcCLB.506469.80	0.00940376110785879
SG5064	TcCLB.508207.250	0.00940376110785879
SG6920	TcCLB.509013.10	0.00940376110785879
SG8458	TcCLB.508613.10	0.00940376110785879
SG8326	TcCLB.511727.230	0.00940452794773178
SG3475	TcCLB.509023.90	0.00944821628048972
SG4659	TcCLB.509537.50	0.00944821628048972
SG2977	TcCLB.506221.120	0.0095578551916933 9
SG7754	TcCLB.508479.310	0.00955785519169339
SG4524	TcCLB.506503.4	0.00958840647473622

Supra gene	Representative ID	FDR
SG0560	TcCLB.504255.30	0.00959541842777066
SG7901	TcCLB.511909.30	0.00959541842777066
SG8143	TcCLB.511807.125	0.00959541842777066
SG1480	TcCLB.508501.190	0.00964511849140425
SG7153	TcCLB.510859.17	0.0097836721664474
SG1238	TcCLB.511727.14	0.00980177685880037
SG3440	TcCLB.510445.50	0.00980177685880037
SG5374	TcCLB.506735.40	0.00980177685880037
SG0533	TcCLB.506107.10	0.00992593808996145
SG2224	TcCLB.510943.30	0.00992593808996145
SG2313	TcCLB.504003.30	0.00992593808996145
SG4260	TcCLB.505983.20	0.00992593808996145
SG4574	TcCLB.509599.60	0.00992593808996145
SG4928	TcCLB.508593.40	0.00992593808996145
SG5106	TcCLB.506989.10	0.00992593808996145
SG6557	TcCLB.508277.340	0.00992593808996145
SG7270	TcCLB.506227.150	0.00992593808996145
SG7283	TcCLB.506289.80	0.00992593808996145
SG6057	TcCLB.509695.50	0.00994909847645916
SG7853	TcCLB.510901.170	0.0101142958225081
SG1116	TcCLB.510611.50	0.010128008393029
SG7914	TcCLB.508547.50	0.0101312032801908
SG3584	TcCLB.509605.30	0.0102587022436057
SG6408	TcCLB.507081.130	0.010274169157903
SG3587	TcCLB.510941.6	0.0102829308351337
SG8951	TcCLB.511727.280	0.0102896688087133
SG2169	TcCLB.507049.60	0.010292051130007
SG2564	TcCLB.509171.47	0.0103091564192627
SG0325	TcCLB.508997.20	0.0103712677535444
SG7702	TcCLB.506855.240	0.010458768709311
SG4241	TcCLB.506989.50	0.0105073443334353
SG3420	TcCLB.511425.40	0.0105507663581782
SG0327	TcCLB.511385.110	0.0106382396735277
SG1705	TcCLB.510689.80	0.0106382396735277
SG1835	TcCLB.507583.20	0.0106382396735277
SG3011	TcCLB.504085.20	0.0106382396735277
SG7411	TcCLB.430895.16	0.0106382396735277
SG8097	TcCLB.508533.50	0.0106382396735277
SG0027	TcCLB.510361.120	0.0106522072206791
SG2217	TcCLB.511751.30	0.0107098772678315
SG2038	TcCLB.506425.80	0.0108377072992836
SG8596	TcCLB.511809.90	0.0108510850320435
SG6024	TcCLB.507515.60	0.0108812571094058
SG5397	TcCLB.509105.80	0.0109765768080001
SG1804	TcCLB.510967.10	0.0111552091423069
SG4481	TcCLB.507001.110	0.0112811517820275
SG4454	TcCLB.506573.70	0.0116072118976117
SG6364	TcCLB.507031.170	0.0116072118976117
SG0200	TcCLB.510187.420	0.0116366245032766
SG2306	TcCLB.509901.100	0.0116366245032766
SG7846	TcCLB.508461.270	0.0116366245032766
SG8765	TcCLB.506679.120	0.0116366245032766
SG4950	TcCLB.508445.90	0.0116480791096686

Supra gene	Representative ID	FDR
SG7297	TcCLB.506625.200	0.0118188351429669
SG0466	TcCLB.509065.60	0.0120827401781295
SG3606	TcCLB.509733.90	0.0120949851042789
SG2072	TcCLB.503945.20	0.0122825768128692
SG6937	TcCLB.445635.10	0.0123683758818686
SG0175	TcCLB.511301.80	0.0123694790994672
SG1673	TcCLB.506201.39	0.0123694790994672
SG1111	TcCLB.509213.10	0.0124671717929523
SG3766	TcCLB.508879.30	0.0124832235492626
SG4602	TcCLB.511165.100	0.0124832235492626
SG8157	TcCLB.511667.40	0.0124832235492626
SG5329	TcCLB.510729.210	0.0125220237651984
SG6926	TcCLB.508347.90	0.0125220237651984
SG1385	TcCLB.509693.110	0.0125261779575383
SG4292	TcCLB.508737.70	0.012583853669876
SG5748	TcCLB.508543.60	0.012583853669876
SG1697	TcCLB.506839.10	0.0126715972889974
SG0311	TcCLB.509599.130	0.0127135074979863
SG3518	TcCLB.511511.150	0.012782412587204
SG2015	TcCLB.507241.30	0.0128518018130285
SG7990	TcCLB.507949.250	0.0128518018130285
SG2267	TcCLB.506835.40	0.0128759258457191
SG3266	TcCLB.510665.30	0.0128759258457191
SG0912	TcCLB.510661.250	0.0129710038793499
SG4688	TcCLB.510421.200	0.0129720068555007
SG7516	TcCLB.506625.70	0.0130465682852214
SG2239	TcCLB.511805.20	0.0131622317042056
SG4385	TcCLB.508891.30	0.0132245275413189
SG5182	TcCLB.509179.200	0.0132245275413189
SG2002	TcCLB.503879.110	0.0132799261450252
SG0995	TcCLB.503893.150	0.0132859677671699
SG5972	TcCLB.510121.40	0.0132859677671699
SG8808	TcCLB.508689.30	0.0132859677671699
SG8829	TcCLB.507007.76	0.0132859677671699
SG1118	TcCLB.508815.179	0.0133443647215021
SG3324	TcCLB.506743.180	0.013432736856035
SG4647	TcCLB.508409.280	0.0134416517335945
SG3618	TcCLB.507911.50	0.0136094419279842
SG0431	TcCLB.508719.9	0.0137631121762468
SG1913	TcCLB.508889.19	0.0138142091579154
SG6665	TcCLB.510949.10	0.0138574361924092
SG0997	TcCLB.509803.40	0.0140766184530029
SG4871	TcCLB.509875.170	0.0142214577211596
SG5448	TcCLB.507951.150	0.0142214577211596
SG8644	TcCLB.507583.40	0.0142214577211596
SG6603	TcCLB.507991.123	0.0142305394389563
SG0196	TcCLB.506243.135	0.0142751881020953
SG4841	TcCLB.506257.60	0.0142751881020953
SG7216	TcCLB.510565.130	0.0142751881020953
SG0245	TcCLB.511657.20	0.0143434447662546
SG1780	TcCLB.506401.280	0.0143434447662546
SG6970	TcCLB.504153.240	0.0143899905381315
SG3943	TcCLB.510155.94	0.0144394683674219

Supra gene	Representative ID	FDR
SG6151	TcCLB.511621.60	0.0144394683674219
SG1107	TcCLB.506605.170	0.0144706425027424
SG1711	TcCLB.509991.9	0.0145153098767824
SG3070	TcCLB.509937.170	0.0145153098767824
SG3931	TcCLB.510729.60	0.0145153098767824
SG6152	TcCLB.510305.70	0.0145153098767824
SG2491	TcCLB.508175.320	0.0146322219991245
SG3759	TcCLB.511755.100	0.0146322219991245
SG6779	TcCLB.433273.10	0.0146695805774787
SG5560	TcCLB.510299.20	0.0146777372141647
SG3928	TcCLB.511725.40	0.0146831631803098
SG8239	TcCLB.508479.274	0.0146831631803098
SG1444	TcCLB.503697.40	0.0147659964563634
SG7179	TcCLB.504147.250	0.0147659964563634
SG0993	TcCLB.507395.10	0.0147843296827369
SG1330	TcCLB.511583.10	0.0147843296827369
SG5322	TcCLB.510661.120	0.0147843296827369
SG3944	TcCLB.511417.70	0.0148352558537491
SG1606	TcCLB.503529.30	0.014852008921546
SG0471	TcCLB.507673.20	0.0148824490982396
SG5096	TcCLB.508823.20	0.0148824490982396
SG5588	TcCLB.503879.50	0.01490787676356
SG8154	TcCLB.505183.120	0.0149548582821253
SG4695	TcCLB.511431.54	0.0150708308142307
SG6236	TcCLB.510337.20	0.0152574198814541
SG0268	TcCLB.509239.10	0.0153056232983115
SG4848	TcCLB.503791.20	0.0153393692869085
SG6852	TcCLB.511867.110	0.0153393692869085
SG2515	TcCLB.505977.13	0.0153833275677075
SG0777	TcCLB.509399.90	0.0153846256052756
SG2924	TcCLB.510409.20	0.0153846256052756
SG5834	TcCLB.511903.270	0.0153846256052756
SG4359	TcCLB.511389.114	0.0154430373994113
SG8401	TcCLB.511725.134	0.0154725957645028
SG2764	TcCLB.506743.190	0.0155749312590401
SG5305	TcCLB.508857.130	0.0158541780022845
SG4472	TcCLB.508821.30	0.0158758745692938
SG7102	TcCLB.503865.60	0.0158758745692938
SG4962	TcCLB.507951.170	0.015877786411932
SG1557	TcCLB.506679.270	0.0158874363926506
SG3393	TcCLB.511635.30	0.0158874363926506
SG3834	TcCLB.511039.6	0.0158874363926506
SG4138	TcCLB.509611.110	0.0159269330975473
SG0217	TcCLB.511873.5	0.0159636264597496
SG5843	TcCLB.504557.10	0.0160850541878601
SG6290	TcCLB.506621.30	0.0160850541878601
SG2452	TcCLB.511291.50	0.016103861020125
SG7291	TcCLB.510101.34	0.0161583538223625
SG8723	TcCLB.508667.40	0.0161583538223625
SG5927	TcCLB.510575.210	0.0166054820381234
SG8966	TcCLB.510187.100	0.0166484119166549
SG7854	TcCLB.510329.160	0.0166951487422914
SG7865	TcCLB.506301.30	0.0166951487422914

Supra gene	Representative ID	FDR
SG8386	TcCLB.510689.30	0.0166951487422914
SG0092	TcCLB.506721.30	0.0167622414592041
SG5973	TcCLB.510125.10	0.016828293525966
SG3621	TcCLB.511629.30	0.0168467476154844
SG2083	TcCLB.508707.80	0.0168530788037488
SG8515	TcCLB.508355.190	0.0168530788037488
SG1766	TcCLB.510105.20	0.0168743323668557
SG2055	TcCLB.507649.40	0.016944722798992
SG7980	TcCLB.507889.20	0.0170212462287199
SG7357	TcCLB.506529.480	0.0170585042490614
SG0488	TcCLB.508153.970	0.0170599889897517
SG2844	TcCLB.510889.340	0.0170879327701814
SG4308	TcCLB.507913.20	0.0171022976523051
SG4515	TcCLB.510729.110	0.0171022976523051
SG5629	TcCLB.510755.19	0.0171022976523051
SG2477	TcCLB.511529.68	0.0172234443884698
SG4455	TcCLB.507737.20	0.0172363344701324
SG5619	TcCLB.508965.70	0.0172363344701324
SG7413	TcCLB.511215.14	0.0172363344701324
SG8309	TcCLB.510943.20	0.0172363344701324
SG6118	TcCLB.511545.90	0.017260373884543
SG4216	TcCLB.510967.20	0.0173909662598554
SG6586	TcCLB.508593.140	0.0174802560938552
SG5368	TcCLB.508507.40	0.0174821388597194
SG0051	TcCLB.511681.20	0.01748931038662
SG1257	TcCLB.504949.30	0.01748931038662
SG2526	TcCLB.506573.34	0.01748931038662
SG5196	TcCLB.510667.10	0.01748931038662
SG1265	TcCLB.510889.330	0.0174893798169804
SG5841	TcCLB.509683.10	0.0174901710759168
SG4907	TcCLB.507897.10	0.017520559731011
SG7460	TcCLB.511313.30	0.0176311943090086
SG7640	TcCLB.507681.100	0.0176311943090086
SG0603	TcCLB.509647.190	0.0176744120075375
SG7093	TcCLB.509979.30	0.0177567706673881
SG3468	TcCLB.511293.90	0.0178222545839119
SG7825	TcCLB.506263.30	0.0178222545839119
SG8759	TcCLB.507063.10	0.0178222545839119
SG0418	TcCLB.511557.50	0.0178622520598737
SG6124	TcCLB.509747.80	0.0178985018271047
SG2305	TcCLB.509207.80	0.0179703137645746
SG2910	TcCLB.507963.10	0.0180305505010604
SG4713	TcCLB.507711.90	0.0180305505010604
SG6322	TcCLB.507993.230	0.0180305505010604
SG6989	TcCLB.506885.90	0.0180873313280849
SG6612	TcCLB.508543.90	0.0181934397181789
SG1037	TcCLB.508981.39	0.0182267373689482
SG2922	TcCLB.506705.60	0.0184548626122932
SG6075	TcCLB.510663.40	0.0184599510428511
SG8395	TcCLB.508257.120	0.0185198038755787
SG1682	TcCLB.506465.40	0.0187468604477543
SG8911	TcCLB.509061.10	0.0190445398129576
SG8081	TcCLB.508153.184	0.0191395703748414

Supra gene	Representative ID	FDR
SG6481	TcCLB.511385.60	0.0191560520315481
SG1167	TcCLB.511895.40	0.0193078986754515
SG7966	TcCLB.508693.190	0.0193078986754515
SG7667	TcCLB.511725.80	0.0195076788997102
SG4847	TcCLB.506009.50	0.0195111274654478
SG1583	TcCLB.509569.90	0.0196251587966259
SG6185	TcCLB.509859.50	0.0196251587966259
SG2178	TcCLB.511633.40	0.0196619345289599
SG2213	TcCLB.511657.50	0.0197913584350692
SG7903	TcCLB.508247.70	0.0199111740908318
SG5505	TcCLB.510425.28	0.0199901081770199
SG2483	TcCLB.508211.70	0.0200990627603289
SG8753	TcCLB.482319.10	0.0201312723729188
SG6411	TcCLB.506177.30	0.0201446796937139
SG7105	TcCLB.511527.70	0.0201446796937139
SG1761	TcCLB.506581.10	0.0202962751698627
SG6454	TcCLB.511229.70	0.0205628731081948
SG0483	TcCLB.483623.10	0.0205636132679325
SG1457	TcCLB.503789.70	0.0205881989462773
SG8466	TcCLB.511391.120	0.0205881989462773
SG3370	TcCLB.503899.100	0.0205895201285443
SG7866	TcCLB.506175.120	0.0206709540227159
SG5795	TcCLB.504827.130	0.0207734196799185
SG3389	TcCLB.506789.160	0.0208013024596473
SG8262	TcCLB.507769.30	0.0209514098391131
SG5540	TcCLB.506883.50	0.0211504922511971
SG0766	TcCLB.509049.30	0.0212061782862149
SG3376	TcCLB.506701.29	0.0213374763217716
SG1036	TcCLB.510749.50	0.0213901185491357
SG2139	TcCLB.508741.190	0.0214393514131505
SG4206	TcCLB.504867.90	0.0216536751306489
SG5742	TcCLB.510007.20	0.0217160832777883
SG6206	TcCLB.507787.100	0.0217160832777883
SG8465	TcCLB.410199.7	0.0217160832777883
SG0735	TcCLB.511467.16	0.0217207204568648
SG3317	TcCLB.508999.200	0.0217207204568648
SG5471	TcCLB.510073.30	0.0217207204568648
SG5659	TcCLB.506419.30	0.0217207204568648
SG8729	TcCLB.506247.40	0.0217207204568648
SG5191	TcCLB.508713.30	0.0220237571425985
SG8302	TcCLB.506335.130	0.0220237571425985
SG1918	TcCLB.509795.30	0.022127965044136
SG4409	TcCLB.510001.20	0.022127965044136
SG7091	TcCLB.506925.450	0.0222462157863537
SG0717	TcCLB.506287.80	0.0224156287246756
SG7218	TcCLB.506925.170	0.0225052174765771
SG6893	TcCLB.511575.90	0.0225141408281806
SG5237	TcCLB.509719.40	0.022728156941367
SG6358	TcCLB.507771.99	0.0227698192586372
SG5340	TcCLB.508999.220	0.0227969022699176
SG5668	TcCLB.510101.59	0.0227969022699176
SG0381	TcCLB.507615.19	0.0229171753064637
SG4111	TcCLB.511655.60	0.0229171753064637

Supra gene	Representative ID	FDR
SG8947	TcCLB.507019.10	0.0229708818320326
SG6363	TcCLB.508119.110	0.0229999961644189
SG3344	TcCLB.511217.70	0.023029063840862
SG1782	TcCLB.510745.49	0.0230981733829224
SG2997	TcCLB.506357.160	0.0234391103140453
SG0595	TcCLB.504575.40	0.02349158562777
SG3105	TcCLB.509053.160	0.0235856541641229
SG3743	TcCLB.511817.10	0.0235856541641229
SG4904	TcCLB.509045.20	0.0235856541641229
SG7599	TcCLB.511211.70	0.0235856541641229
SG8131	TcCLB.504045.30	0.0235856541641229
SG0514	TcCLB.508015.10	0.0237447543017074
SG3378	TcCLB.509243.20	0.0242058915141501
SG1254	TcCLB.506733.80	0.0243736656608012
SG6680	TcCLB.511295.10	0.0243736656608012
SG7365	TcCLB.503479.70	0.0243736656608012
SG6164	TcCLB.511277.500	0.0244090093990376
SG6496	TcCLB.506297.350	0.0245046051367006
SG4190	TcCLB.509835.30	0.0245322657192031
SG6606	TcCLB.511263.40	0.0245322657192031
SG4903	TcCLB.511253.20	0.0248698590944527
SG5072	TcCLB.507831.40	0.0248698590944527
SG1742	TcCLB.509605.20	0.0248701391595242
SG3661	TcCLB.511657.40	0.0248701391595242
SG8258	TcCLB.506247.50	0.0248701391595242
SG5464	TcCLB.508737.150	0.0249175057892785
SG8498	TcCLB.511807.190	0.025064397385875
SG5252	TcCLB.506411.30	0.0254308950479314
SG1211	TcCLB.508811.10	0.0254531834389226
SG8405	TcCLB.504105.100	0.0254531834389226
SG8433	TcCLB.507681.30	0.0254531834389226
SG7109	TcCLB.511181.110	0.0255751359490014
SG6394	TcCLB.507041.120	0.0255967824572165
SG7585	TcCLB.507099.90	0.0257208354778338
SG0985	TcCLB.508129.9	0.0258777331429715
SG0601	TcCLB.509805.210	0.0258960959625617
SG6658	TcCLB.507019.83	0.0260114347272649
SG4094	TcCLB.506505.10	0.0261332926865498
SG7798	TcCLB.511491.130	0.0262047661031381
SG7111	TcCLB.509769.40	0.0263462104840861
SG5277	TcCLB.511165.30	0.0265132591225033
SG8066	TcCLB.506839.30	0.0265132591225033
SG7034	TcCLB.504057.40	0.0269596621614689
SG7441	TcCLB.506945.310	0.0269596621614689
SG7700	TcCLB.510329.180	0.0269596621614689
SG5045	TcCLB.503541.10	0.0273455413780259
SG5603	TcCLB.509033.60	0.0273498761049355
SG4082	TcCLB.509505.20	0.0275728413475845
SG2602	TcCLB.511761.40	0.0275983957759491
SG1938	TcCLB.503559.90	0.0277057267427942
SG0669	TcCLB.504023.10	0.0278480279407557
SG0037	TcCLB.506435.370	0.0278639599092353
SG4477	TcCLB.506979.20	0.0278640908082381

Supra gene	Representative ID	FDR
SG8664	TcCLB.509647.26	0.0279532639421914
SG4440	TcCLB.510969.40	0.028214337292649
SG5339	TcCLB.511431.90	0.0282423025198797
SG2847	TcCLB.509791.80	0.0282655679044123
SG6362	TcCLB.510311.100	0.0283040076488786
SG0816	TcCLB.506829.110	0.028541234525014
SG4657	TcCLB.510259.24	0.0286467941926518
SG3920	TcCLB.510825.20	0.0286880212394743
SG6184	TcCLB.508231.170	0.0286880212394743
SG7066	TcCLB.510513.70	0.0287021212066985
SG7362	TcCLB.510431.190	0.0293122217588983
SG0194	TcCLB.509505.50	0.0295229477281763
SG0640	TcCLB.507951.299	0.0295741018785718
SG0859	TcCLB.511385.90	0.0296105360161305
SG5824	TcCLB.511151.80	0.0297103284088614
SG5509	TcCLB.510579.50	0.0299821352768329
SG5802	TcCLB.511821.90	0.0299821352768329
SG8086	TcCLB.506721.50	0.0299821352768329
SG4245	TcCLB.503505.14	0.0301793433389779
SG7016	TcCLB.506375.80	0.0301793433389779
SG1829	TcCLB.507057.40	0.0304575845333712
SG3582	TcCLB.503975.20	0.0304575845333712
SG4320	TcCLB.508699.40	0.0304575845333712
SG5304	TcCLB.510357.10	0.0304575845333712
SG5627	TcCLB.510663.80	0.0304575845333712
SG8505	TcCLB.508257.180	0.0304575845333712
SG4989	TcCLB.510225.50	0.0305075892513768
SG8076	TcCLB.506247.110	0.0306906112085054
SG2731	TcCLB.504253.20	0.0308443347344194
SG2818	TcCLB.511289.40	0.0308443347344194
SG5090	TcCLB.506559.4	0.0308443347344194
SG5773	TcCLB.511389.100	0.0308443347344194
SG5710	TcCLB.509319.20	0.0309468368535741
SG2150	TcCLB.509027.60	0.0311390693705143
SG4334	TcCLB.507857.10	0.0311390693705143
SG4367	TcCLB.503733.20	0.0311390693705143
SG0593	TcCLB.504053.10	0.0311636861665522
SG5238	TcCLB.507943.60	0.0311995641779034
SG1309	TcCLB.509029.60	0.0313204333416644
SG3933	TcCLB.508543.20	0.0313204333416644
SG6587	TcCLB.509715.70	0.0313204333416644
SG6621	TcCLB.508905.10	0.0313204333416644
SG0506	TcCLB.433677.10	0.031660112298406
SG4942	TcCLB.510045.30	0.0316950376592645
SG6915	TcCLB.506925.110	0.0316950376592645
SG3503	TcCLB.506479.80	0.0317118079190839
SG5050	TcCLB.511517.150	0.0317802894624169
SG1861	TcCLB.509937.160	0.0317982453040963
SG1284	TcCLB.506585.100	0.0318118326267312
SG0490	TcCLB.511287.30	0.0318571436696998
SG2151	TcCLB.506855.60	0.0318571436696998
SG3607	TcCLB.510443.30	0.0318571436696998
SG3698	TcCLB.510889.231	0.0318571436696998

Supra gene	Representative ID	FDR
SG0121	TcCLB.511401.90	0.0322483672085141
SG0996	TcCLB.508153.20	0.0322975242021433
SG2300	TcCLB.509911.40	0.0323854175995403
SG2601	TcCLB.504013.100	0.0323854175995403
SG6956	TcCLB.510535.20	0.032447841543767
SG3594	TcCLB.504125.64	0.0324515709377783
SG3332	TcCLB.508257.170	0.0325033210362346
SG4384	TcCLB.510333.10	0.0325033210362346
SG5436	TcCLB.508357.90	0.0325033210362346
SG6063	TcCLB.509911.110	0.0325033210362346
SG6082	TcCLB.511261.80	0.0325033210362346
SG7601	TcCLB.507093.250	0.0325033210362346
SG1516	TcCLB.508731.40	0.032601302082327
SG5234	TcCLB.503557.40	0.032601302082327
SG2430	TcCLB.510825.30	0.0327428644569249
SG2263	TcCLB.507029.60	0.0327958390169205
SG8857	TcCLB.510817.60	0.0328431912388684
SG0635	TcCLB.506775.30	0.0331966472809854
SG4191	TcCLB.509103.20	0.0333428523164522
SG6019	TcCLB.511001.70	0.0333428523164522
SG6599	TcCLB.511817.20	0.0334159710449389
SG8016	TcCLB.507073.30	0.0335981069694722
SG0945	TcCLB.511555.110	0.0336602277495831
SG0868	TcCLB.504625.70	0.033839964336738
SG1142	TcCLB.510649.24	0.033839964336738
SG4106	TcCLB.503747.40	0.0338481841154549
SG7954	TcCLB.510187.470	0.0338711167563309
SG7287	TcCLB.448567.9	0.0338888569496616
SG5459	TcCLB.506807.20	0.0339883902972654
SG8106	TcCLB.506977.110	0.0340083882192818
SG0459	TcCLB.510605.19	0.0341637746876202
SG2114	TcCLB.504027.10	0.0341637746876202
SG0290	TcCLB.509943.20	0.03416667948868
SG4381	TcCLB.508199.30	0.0344652863521806
SG5538	TcCLB.504741.124	0.0344652863521806
SG8210	TcCLB.506041.60	0.0345675190293357
SG8736	TcCLB.511725.174	0.0345675190293357
SG0338	TcCLB.507105.40	0.0349124531099039
SG1879	TcCLB.504797.130	0.0349124531099039
SG2092	TcCLB.503687.20	0.0349274259614672
SG7334	TcCLB.504147.224	0.0350099537048003
SG1781	TcCLB.511167.20	0.0351487555121114
SG3283	TcCLB.508153.640	0.0351487555121114
SG2286	TcCLB.508193.60	0.0351744859677329
SG8971	TcCLB.511249.44	0.0352279487399138
SG4670	TcCLB.511389.110	0.0353702323975136
SG7707	TcCLB.510187.280	0.0356509428176841
SG0476	TcCLB.504191.10	0.0357953773309678
SG4572	TcCLB.508625.60	0.0359183163274713
SG8948	TcCLB.506445.110	0.0359490658897322
SG0024	TcCLB.404711.10	0.0360508639890638
SG0042	TcCLB.510787.10	0.0360508639890638
SG5137	TcCLB.506937.40	0.0360508639890638

Supra gene	Representative ID	FDR
SG0101	TcCLB.506499.105	0.0360816593607279
SG0190	TcCLB.506885.210	0.0360816593607279
SG2609	TcCLB.504149.20	0.0361802372977172
SG2281	TcCLB.506581.30	0.0365293746912955
SG0384	TcCLB.505171.10	0.0365717727452759
SG0255	TcCLB.507023.220	0.0366350531411375
SG1301	TcCLB.506959.80	0.0366350531411375
SG3549	TcCLB.509979.40	0.0366350531411375
SG3702	TcCLB.509695.140	0.0366350531411375
SG6833	TcCLB.511215.20	0.0366350531411375
SG7047	TcCLB.503651.4	0.0366350531411375
SG7938	TcCLB.507069.120	0.0366356767643574
SG2558	TcCLB.505999.60	0.0367594156487892
SG0328	TcCLB.507533.21	0.0368054195309378
SG4131	TcCLB.507007.74	0.0368350765820373
SG0557	TcCLB.510771.9	0.0368425263893942
SG3504	TcCLB.504231.10	0.0368425263893942
SG2793	TcCLB.509229.60	0.0368970637072248
SG0020	TcCLB.510583.80	0.0371110144011556
SG1017	TcCLB.504199.10	0.0371735971593123
SG3867	TcCLB.510355.250	0.0377808136513286
SG5639	TcCLB.506181.10	0.0381436413560502
SG0658	TcCLB.504013.110	0.0383285077384928
SG6417	TcCLB.506321.340	0.0384061142135206
SG8864	TcCLB.508257.90	0.0384061142135206
SG8213	TcCLB.508547.190	0.0384420491309382
SG0732	TcCLB.511329.10	0.038480321926001
SG2494	TcCLB.508411.60	0.038486551944908
SG1991	TcCLB.508445.30	0.0384964396850506
SG2376	TcCLB.503703.60	0.0385969955432223
SG2952	TcCLB.506145.70	0.0386683655265791
SG3403	TcCLB.508111.30	0.0386683655265791
SG4506	TcCLB.509731.10	0.0387158482648549
SG7867	TcCLB.508409.110	0.0387158482648549
SG3414	TcCLB.506195.120	0.0388721531992608
SG7051	TcCLB.504057.80	0.0390916796790294
SG8023	TcCLB.508173.90	0.0393344108129321
SG3580	TcCLB.510667.30	0.0402899644922253
SG2897	TcCLB.508213.40	0.0405832836853079
SG3860	TcCLB.508893.10	0.040700198973831
SG6966	TcCLB.510295.15	0.0407617049002505
SG0827	TcCLB.505193.50	0.040844778963048
SG0575	TcCLB.510755.40	0.0409245736122337
SG1486	TcCLB.503567.9	0.0409245736122337
SG5489	TcCLB.508909.214	0.0409245736122337
SG8855	TcCLB.507681.210	0.0409245736122337
SG0274	TcCLB.507875.170	0.0410531675908255
SG0150	TcCLB.506279.140	0.041329999707117
SG1927	TcCLB.510309.10	0.041329999707117
SG6128	TcCLB.508503.60	0.041329999707117
SG4044	TcCLB.503727.10	0.0415190650335727
SG4891	TcCLB.503891.50	0.041526753420152 8
SG0882	TcCLB.509029.40	0.0415973077393345

Supra gene	Representative ID	FDR
SG4201	TcCLB.508809.50	0.0415973077393345
SG6576	TcCLB.510859.10	0.0415973077393345
SG1433	TcCLB.511577.188	0.0420328808427815
SG0800	TcCLB.508717.50	0.0420464837675808
SG6979	TcCLB.507875.140	0.0420845429445904
SG0102	TcCLB.508221.539	0.0421847097078823
SG0990	TcCLB.510657.70	0.0421847097078823
SG2674	TcCLB.506297.230	0.0421847097078823
SG4596	TcCLB.425969.5	0.0422905052860667
SG3758	TcCLB.507047.134	0.0422993848440914
SG5827	TcCLB.508545.20	0.0422993848440914
SG7395	TcCLB.504147.40	0.0422993848440914
SG3138	TcCLB.510635.40	0.042454960622619
SG5230	TcCLB.510665.60	0.042454960622619
SG6782	TcCLB.506945.300	0.0425514377681104
SG0589	TcCLB.511837.110	0.0425557129286181
SG3220	TcCLB.507915.10	0.0425557129286181
SG4224	TcCLB.509065.120	0.0425557129286181
SG6539	TcCLB.509623.10	0.0425557129286181
SG7663	TcCLB.507053.140	0.0425557129286181
SG8107	TcCLB.510979.60	0.0425557129286181
SG0159	TcCLB.507547.18	0.0429696861244894
SG7911	TcCLB.506425.60	0.0430314428331553
SG1959	TcCLB.508397.10	0.0432768993265261
SG3945	TcCLB.504797.100	0.0433590574496301
SG3125	TcCLB.509153.10	0.0434911909280492
SG0340	TcCLB.511825.200	0.0435819366385118
SG4711	TcCLB.506577.140	0.043791178026514
SG5097	TcCLB.506815.40	0.043791178026514
SG3134	TcCLB.511585.60	0.0438481385027002
SG5456	TcCLB.508351.40	0.0438481385027002
SG6154	TcCLB.508641.180	0.0438481385027002
SG7244	TcCLB.511869.9	0.0438481385027002
SG1589	TcCLB.508999.250	0.0439969704869299
SG0430	TcCLB.503989.50	0.0440743176284642
SG8134	TcCLB.506425.90	0.0441875167460395
SG5061	TcCLB.510725.40	0.0443151957842966
SG0434	TcCLB.509891.70	0.0445145320055697
SG3802	TcCLB.507705.10	0.0445145320055697
SG4114	TcCLB.511245.79	0.0445145320055697
SG5364	TcCLB.506351.35	0.0446458617383582
SG5153	TcCLB.511237.110	0.0447587642056845
SG5291	TcCLB.508943.20	0.0447587642056845
SG1449	TcCLB.503977.30	0.0448161002050657
SG3711	TcCLB.511021.90	0.0448338940864881
SG4773	TcCLB.503415.30	0.0448338940864881
SG0197	TcCLB.507883.10	0.0452316400330936
SG4093	TcCLB.503837.20	0.0453788389803751
SG0521	TcCLB.455721.9	0.0453809680589408
SG1703	TcCLB.506957.120	0.0455039280900281
SG7638	TcCLB.508257.220	0.0455039280900281
SG5265	TcCLB.511389.130	0.0456095001387433
SG5443	TcCLB.509151.90	0.0456608416906094

Supra gene	Representative ID	FDR
SG6497	TcCLB.510735.60	0.0459083099546905
SG2835	TcCLB.504069.10	0.046038952891882
SG2391	TcCLB.507787.80	0.0464909084260118
SG8470	TcCLB.507053.10	0.0467027711481378
SG0496	TcCLB.509007.99	0.0467921595884495
SG0728	TcCLB.507991.10	0.0467921595884495
SG0980	TcCLB.504069.56	0.0467921595884495
SG4759	TcCLB.509331.30	0.0467921595884495
SG6595	TcCLB.407335.9	0.0467921595884495
SG8315	TcCLB.510187.210	0.0467921595884495
SG8867	TcCLB.511277.180	0.0467921595884495
SG1513	TcCLB.503913.40	0.0468251068415262
SG4694	TcCLB.510421.290	0.0468251068415262
SG3770	TcCLB.509317.90	0.0470867310411962
SG5833	TcCLB.506697.10	0.0473621680408309
SG4760	TcCLB.506685.40	0.047788821060163
SG4265	TcCLB.511407.40	0.0482538889463553
SG2703	TcCLB.509601.100	0.0484475539710918
SG3608	TcCLB.511753.90	0.0486883513689751
SG3918	TcCLB.507073.20	0.0486883513689751
SG8762	TcCLB.511367.240	0.0486883513689751
SG8194	TcCLB.511807.160	0.0486914328711686
SG4153	TcCLB.508199.20	0.0488174603771544
SG4501	TcCLB.503945.40	0.0488174603771544
SG1364	TcCLB.511575.30	0.0489502044479704
SG4732	TcCLB.506413.70	0.0489502044479704
SG4751	TcCLB.508777.40	0.0490404989527962
SG2929	TcCLB.424123.20	0.0492157025856115
SG1977	TcCLB.509541.10	0.0492673842539305
SG8850	TcCLB.506247.80	0.0493817349571399
SG8196	TcCLB.511895.50	0.0495866141109
SG3857	TcCLB.506735.70	0.0496272056135567
SG3380	TcCLB.503823.30	0.049938944224417
SG7392	TcCLB.504125.60	0.049938944224417
SG8781	TcCLB.510329.20	0.049938944224417
SG3820	TcCLB.506227.160	0.0500056571722497
SG0727	TcCLB.508411.50	0.0503430996030815
SG5408	TcCLB.508507.50	0.050356828497984
SG3734	TcCLB.507317.40	0.0504239933467584
SG2655	TcCLB.510257.60	0.050561631332042
SG3029	TcCLB.511837.90	0.050561631332042
SG7751	TcCLB.508153.140	0.050561631332042
SG3469	TcCLB.510531.40	0.0506377747951142
SG5838	TcCLB.508501.250	0.0507180251670306
SG3274	TcCLB.508881.20	0.0507236028380874
SG3102	TcCLB.509023.120	0.0508617002615517
SG3446	TcCLB.511535.30	0.0508617002615517
SG5816	TcCLB.503897.70	0.0508617002615517
SG6134	TcCLB.509791.30	0.0508617002615517
SG0768	TcCLB.506247.370	0.0509518873255387
SG0733	TcCLB.508153.370	0.0509578462915099
SG1354	TcCLB.509747.50	0.0509578462915099
SG2738	TcCLB.509023.50	0.0509578462915099

Supra gene	Representative ID	FDR
SG4148	TcCLB.504131.130	0.0514002749000654
SG4798	TcCLB.511245.190	0.0514882502440141
SG1392	TcCLB.506989.30	0.0519615663088109
SG6162	TcCLB.511287.20	0.0519615663088109
SG8501	TcCLB.511389.70	0.0519615663088109
SG0047	TcCLB.508291.50	0.0521360428185652
SG2912	TcCLB.510953.30	0.0521399241271439
SG4619	TcCLB.508901.20	0.0523915611548842
SG2501	TcCLB.508851.110	0.0525832787487145
SG4370	TcCLB.508667.49	0.0525963133622681
SG3814	TcCLB.508153.680	0.0527412943412306
SG7234	TcCLB.507429.60	0.0527990389231757
SG6968	TcCLB.507099.80	0.0534377562358688
SG5295	TcCLB.511825.80	0.0534957476957757
SG5351	TcCLB.511817.134	0.0535411690234516
SG7269	TcCLB.509583.19	0.053703303678705
SG2824	TcCLB.509005.60	0.0537399503403863
SG1940	TcCLB.508153.240	0.0537966840911445
SG1970	TcCLB.507081.20	0.0538080782012855
SG5939	TcCLB.503847.80	0.0538080782012855
SG8797	TcCLB.506559.470	0.0538080782012855
SG0425	TcCLB.503781.10	0.0539416383767004
SG6276	TcCLB.503885.100	0.0543841594134217
SG8640	TcCLB.422955.10	0.0544176407570344
SG1947	TcCLB.509063.4	0.0545089643401786
SG0875	TcCLB.508837.80	0.0548054331098046
SG6992	TcCLB.507521.115	0.0549701179897533
SG3115	TcCLB.511321.60	0.0550075630655266
SG3822	TcCLB.506469.90	0.0552581307945901
SG0120	TcCLB.506355.100	0.055617073897996
SG0953	TcCLB.508153.610	0.055617073897996
SG1388	TcCLB.509611.150	0.055617073897996
SG1850	TcCLB.510155.10	0.055617073897996
SG3017	TcCLB.506195.160	0.055617073897996
SG6434	TcCLB.508865.30	0.055617073897996
SG6762	TcCLB.511189.70	0.055617073897996
SG7534	TcCLB.506737.100	0.055617073897996
SG5433	TcCLB.458759.14	0.0557015011475991
SG7141	TcCLB.508307.134	0.0557015011475991
SG0044	TcCLB.511593.40	0.0557148347222366
SG4095	TcCLB.506479.120	0.0558291152328582
SG6889	TcCLB.508147.140	0.0560773257507475
SG3058	TcCLB.510575.120	0.0561859534055737
SG8382	TcCLB.509995.20	0.0561859534055737
SG7591	TcCLB.503479.40	0.0562112591750172
SG4581	TcCLB.509051.40	0.0562476968416002
SG3915	TcCLB.506851.20	0.056282393638208
SG8619	TcCLB.508257.150	0.056282393638208
SG4536	TcCLB.506319.70	0.0565660060176457
SG0634	TcCLB.511067.20	0.0566373832101047
SG2619	TcCLB.509229.30	0.0566373832101047
SG7155	TcCLB.507099.100	0.0567034116108563
SG0464	TcCLB.510339.50	0.0568430528308697

Supra gene	Representative ID	FDR
SG8453	TcCLB.508479.213	0.0568430528308697
SG5650	TcCLB.503929.20	0.0568497353267074
SG3048	TcCLB.509151.140	0.0570465601809308
SG5541	TcCLB.506999.120	0.0570465601809308
SG7462	TcCLB.509455.114	0.0570465601809308
SG0100	TcCLB.508629.100	0.0572859248623029
SG0749	TcCLB.504051.40	0.0572859248623029
SG0848	TcCLB.508461.80	0.0573806582415284
SG0814	TcCLB.508909.330	0.0574689254515559
SG3893	TcCLB.507951.60	0.057532894769663
SG4040	TcCLB.508601.90	0.057532894769663
SG5271	TcCLB.506777.30	0.0575859814209165
SG1529	TcCLB.503487.60	0.0578299664671324
SG6471	TcCLB.509051.20	0.057925386595239
SG7573	TcCLB.504153.90	0.057925386595239
SG0439	TcCLB.508821.50	0.0580163961389541
SG2031	TcCLB.506399.70	0.0580163961389541
SG2568	TcCLB.506855.180	0.0580163961389541
SG3219	TcCLB.511817.40	0.0581172374470769
SG0765	TcCLB.509575.30	0.0584513071772309
SG3168	TcCLB.508413.60	0.0584513071772309
SG5294	TcCLB.503891.40	0.0584513071772309
SG6765	TcCLB.509635.9	0.0584513071772309
SG1304	TcCLB.508707.130	0.0585579134225869
SG0664	TcCLB.508909.170	0.0586060905395898
SG0152	TcCLB.507965.10	0.0586420613389104
SG3176	TcCLB.511051.10	0.0586463214790693
SG6798	TcCLB.510269.10	0.0586463214790693
SG3733	TcCLB.510421.29	0.0587644258036511
SG1153	TcCLB.507737.30	0.0587750422124261
SG8155	TcCLB.510187.480	0.0587855066361286
SG8089	TcCLB.506445.100	0.0587916830003865
SG4464	TcCLB.509567.50	0.0588081461532528
SG4971	TcCLB.509229.130	0.0588081461532528
SG7547	TcCLB.506625.150	0.0588081461532528
SG5819	TcCLB.511439.90	0.0588976687224272
SG5597	TcCLB.503999.60	0.0590019218989674
SG0116	TcCLB.508293.60	0.0590046655016879
SG0158	TcCLB.507747.10	0.059256377998674
SG2789	TcCLB.506691.60	0.059256377998674
SG1015	TcCLB.504147.120	0.0593314688814002
SG5356	TcCLB.508135.10	0.0593314688814002
SG4349	TcCLB.503581.30	0.0594617439053677
SG2017	TcCLB.511661.40	0.0594639398412584
SG2248	TcCLB.508153.560	0.0594639398412584
SG4626	TcCLB.509049.20	0.059655428402366
SG4996	TcCLB.507257.90	0.059655428402366
SG5008	TcCLB.511245.160	0.059655428402366
SG1652	TcCLB.509799.90	0.0597782052228401
SG5148	TcCLB.511267.24	0.0598976761928715
SG1988	TcCLB.507049.10	0.0600144953060368
SG1315	TcCLB.509207.120	0.0602697539030073
SG5698	TcCLB.511529.250	0.0602697539030073

Supra gene	Representative ID	FDR
SG7197	TcCLB.511211.49	0.0603172652811238
SG2343	TcCLB.506619.90	0.0603791605887808
SG6173	TcCLB.511815.60	0.0607259668372798
SG5181	TcCLB.507895.100	0.0608222774485896
SG5797	TcCLB.503943.14	0.0608568272007583
SG5831	TcCLB.508153.634	0.0614006544706451
SG1345	TcCLB.506825.200	0.0614940312271365
SG0284	TcCLB.511807.100	0.0615207683793502
SG7013	TcCLB.511421.185	0.0618369991365174
SG0948	TcCLB.508625.100	0.0620737481298151
SG3400	TcCLB.510823.80	0.0622081100828708
SG3427	TcCLB.507945.20	0.0622252635424944
SG5363	TcCLB.508707.227	0.0625229168844727
SG7245	TcCLB.511867.120	0.0626506113001617
SG8696	TcCLB.511511.6	0.0627564939689412
SG0052	TcCLB.506289.140	0.0629822139659322
SG3935	TcCLB.509507.40	0.063018688977466
SG4444	TcCLB.505193.100	0.0631165811143532
SG4628	TcCLB.507017.70	0.0635588251787945
SG6675	TcCLB.510339.100	0.0635588251787945
SG8573	TcCLB.507053.20	0.0636976976221871
SG6972	TcCLB.510431.230	0.0637593617605927
SG4941	TcCLB.510359.20	0.0642544736073884
SG6256	TcCLB.511507.70	0.0642544736073884
SG5506	TcCLB.508387.100	0.0643915515635975
SG2201	TcCLB.504175.40	0.0652127389466104
SG3374	TcCLB.507801.160	0.0652127389466104
SG3646	TcCLB.511277.560	0.0652127389466104
SG7169	TcCLB.504153.70	0.0652127389466104
SG1297	TcCLB.506529.260	0.0653051523722173
SG4100	TcCLB.504243.20	0.0653471663436577
SG2260	TcCLB.509027.50	0.0657903582157372
SG4980	TcCLB.511511.60	0.0658771713906583
SG2538	TcCLB.510659.230	0.0665262373501167
SG7639	TcCLB.509527.130	0.06662381391616
SG2596	TcCLB.506985.40	0.0666895097059033
SG5548	TcCLB.510351.90	0.0666895097059033
SG0296	TcCLB.508939.50	0.0671467855931207
SG0750	TcCLB.508153.460	0.0671467855931207
SG0188	TcCLB.511173.80	0.0673310340897128
SG1259	TcCLB.508075.50	0.0673310340897128
SG2034	TcCLB.507963.60	0.0674258934552342
SG3130	TcCLB.509539.50	0.0674258934552342
SG5952	TcCLB.508547.80	0.0674258934552342
SG1903	TcCLB.510835.10	0.06790043905668
SG6387	TcCLB.504427.50	0.0681532529608935
SG1054	TcCLB.507011.120	0.0681653005095744
SG4614	TcCLB.504185.40	0.0681653005095744
SG6338	TcCLB.511653.60	0.0682512662443841
SG5805	TcCLB.503635.10	0.0685035116233497
SG2115	TcCLB.509563.40	0.0685097324661339
SG2879	TcCLB.511661.90	0.0686420516598029
SG5261	TcCLB.509795.40	0.0686420516598029

Supra gene	Representative ID	FDR
SG6946	TcCLB.510877.100	0.0689288277229423
SG0961	TcCLB.509939.19	0.0689423103042054
SG4446	TcCLB.511859.30	0.0689423103042054
SG5656	TcCLB.504013.30	0.0691863484651608
SG6337	TcCLB.506247.20	0.0691934504681385
SG2022	TcCLB.504003.80	0.0693447565129414
SG4767	TcCLB.503625.10	0.06939589268365
SG5728	TcCLB.511139.10	0.06939589268365
SG0377	TcCLB.508621.10	0.0695918973666453
SG7341	TcCLB.506625.180	0.0696037071383382
SG3971	TcCLB.506241.170	0.0699273436390914
SG4162	TcCLB.505193.84	0.0699273436390914
SG5933	TcCLB.510889.221	0.0699273436390914
SG5452	TcCLB.440099.10	0.0702096122693611
SG5470	TcCLB.508109.14	0.0702096122693611
SG6828	TcCLB.506925.400	0.0702096122693611
SG7246	TcCLB.506625.210	0.0702923238434033
SG0617	TcCLB.504575.20	0.0703731564065354
SG4083	TcCLB.509151.60	0.070525046150614
SG0072	TcCLB.505699.10	0.0707171513789418
SG1502	TcCLB.509643.50	0.0707171513789418
SG2294	TcCLB.506851.30	0.0707171513789418
SG2393	TcCLB.511281.40	0.0707171513789418
SG3052	TcCLB.510661.270	0.0707171513789418
SG3426	TcCLB.503809.120	0.0707171513789418
SG3785	TcCLB.511269.40	0.0707171513789418
SG4009	TcCLB.506287.120	0.0707171513789418
SG4157	TcCLB.504073.20	0.0707171513789418
SG5241	TcCLB.510065.30	0.0707171513789418
SG5260	TcCLB.511555.20	0.0707171513789418
SG5418	TcCLB.510339.54	0.0707171513789418
SG8656	TcCLB.511283.280	0.0707171513789418
SG1042	TcCLB.509767.200	0.0708760670523942
SG7922	TcCLB.510873.10	0.0710691162311879
SG1585	TcCLB.511671.90	0.0711564489257653
SG3526	TcCLB.508909.20	0.0711564489257653
SG6340	TcCLB.511741.50	0.0711564489257653
SG5818	TcCLB.509911.100	0.0713509855793492
SG1495	TcCLB.509065.36	0.0714365944718919
SG6188	TcCLB.509179.130	0.0717696254131197
SG2571	TcCLB.509167.70	0.0718439356252158
SG2014	TcCLB.506575.54	0.071928624390026
SG2664	TcCLB.506501.240	0.071928624390026
SG7811	TcCLB.508737.60	0.071928624390026
SG4065	TcCLB.506435.170	0.0720260077860675
SG2032	TcCLB.508351.10	0.0721233864172174
SG2846	TcCLB.509399.180	0.0724059482162094
SG1902	TcCLB.510663.50	0.0727559501753315
SG3677	TcCLB.508817.10	0.0727559501753315
SG5033	TcCLB.508153.180	0.0727559501753315
SG5732	TcCLB.509789.40	0.0727559501753315
SG0495	TcCLB.507809.110	0.0729381159662476
SG4587	TcCLB.511245.150	0.0729381159662476

Supra gene	Representative ID	FDR
SG0610	TcCLB.504167.30	0.0729539341782703
SG3818	TcCLB.503823.140	0.0729539341782703
SG1442	TcCLB.511499.50	0.073107646605068
SG3349	TcCLB.511543.60	0.073107646605068
SG4858	TcCLB.509571.10	0.073107646605068
SG6489	TcCLB.503917.7	0.073107646605068
SG2980	TcCLB.508817.110	0.0734696526939942
SG6551	TcCLB.507603.110	0.0735838628647011
SG3072	TcCLB.509033.80	0.0736430000357049
SG3755	TcCLB.511517.30	0.0736430000357049
SG4665	TcCLB.503583.80	0.0738488983006901
SG4218	TcCLB.509715.140	0.0740322655591747
SG7426	TcCLB.506543.100	0.074217744929861
SG0691	TcCLB.508215.9	0.0743344192504723
SG7354	TcCLB.509585.10	0.0743344192504723
SG7775	TcCLB.506559.430	0.0743346516926458
SG8357	TcCLB.506559.380	0.0744398178257276
SG3660	TcCLB.506445.40	0.0744524949313577
SG8170	TcCLB.508827.50	0.0745377777584233
SG1960	TcCLB.507735.20	0.0747534154562697
SG5640	TcCLB.510421.180	0.0747534154562697
SG5832	TcCLB.503769.40	0.0748539542757372
SG1739	TcCLB.509567.10	0.0749010271449666
SG5740	TcCLB.436521.9	0.0751705288363305
SG4911	TcCLB.509099.30	0.0751759603782904
SG7103	TcCLB.511175.20	0.0751759603782904
SG1447	TcCLB.509717.60	0.0752325294978034
SG1607	TcCLB.511647.10	0.0754482315677118
SG4373	TcCLB.511871.90	0.0754482315677118
SG8082	TcCLB.511491.30	0.0754482315677118
SG3334	TcCLB.507023.110	0.0754707420939776
SG3467	TcCLB.503617.10	0.0757633169673115
SG0455	TcCLB.509823.4	0.0758285045427125
SG1548	TcCLB.511865.40	0.0758285045427125
SG3991	TcCLB.511807.40	0.0759236735775179
SG6096	TcCLB.509643.70	0.0759236735775179
SG6487	TcCLB.509471.10	0.0759236735775179
SG2747	TcCLB.509611.130	0.0759542408366919
SG5738	TcCLB.509965.394	0.0759542408366919
SG2390	TcCLB.506337.20	0.0763942160634593
SG6280	TcCLB.511635.40	0.076830652607678
SG0887	TcCLB.506851.10	0.0768515045787677
SG1347	TcCLB.504113.30	0.0768515045787677
SG1605	TcCLB.509937.220	0.0768515045787677
SG8668	TcCLB.507209.10	0.0768515045787677
SG2030	TcCLB.506989.150	0.0768871899208187
SG4434	TcCLB.511153.64	0.0768871899208187
SG8000	TcCLB.511277.330	0.0769276224491312
SG1691	TcCLB.503419.20	0.0769480826582592
SG2665	TcCLB.511517.70	0.0769480826582592
SG3402	TcCLB.506529.50	0.0769480826582592
SG7520	TcCLB.506625.84	0.0769480826582592
SG5362	TcCLB.509471.30	0.0769835994163448

Supra gene	Representative ID	FDR
SG4726	TcCLB.510065.40	0.0771702674621784
SG1534	TcCLB.508851.79	0.0771728323158276
SG2711	TcCLB.504867.10	0.0772890426714998
SG7210	TcCLB.504153.120	0.0777314085386046
SG4586	TcCLB.503797.10	0.0778591768688618
SG8447	TcCLB.511277.290	0.0779117190103046
SG0271	TcCLB.508739.40	0.0779482256718922
SG1020	TcCLB.508625.160	0.0779482256718922
SG1178	TcCLB.510347.20	0.0779482256718922
SG6214	TcCLB.506855.350	0.0779482256718922
SG7986	TcCLB.511907.310	0.07798187796167
SG5755	TcCLB.508893.20	0.0785143335495312
SG4259	TcCLB.504079.10	0.0785741809486499
SG0650	TcCLB.506181.70	0.0786512559727343
SG0792	TcCLB.508065.60	0.0786512559727343
SG2973	TcCLB.511517.160	0.0786512559727343
SG2513	TcCLB.509059.50	0.0788501915869284
SG1066	TcCLB.510433.10	0.0792423554134897
SG8806	TcCLB.508461.410	0.0792423554134897
SG1720	TcCLB.503891.54	0.0792953610595557
SG7964	TcCLB.508827.20	0.0793871550544685
SG0235	TcCLB.504703.20	0.0794056409907276
SG8077	TcCLB.508251.40	0.0794056409907276
SG8350	TcCLB.510483.250	0.0794402595246576
SG1948	TcCLB.511747.30	0.0795742821507744
SG8881	TcCLB.510183.10	0.0797511824038872
SG7097	TcCLB.506763.260	0.0798275952320111
SG2137	TcCLB.510155.110	0.0798345903056067
SG7385	TcCLB.508227.20	0.0798345903056067
SG5685	TcCLB.511823.70	0.0798460484007876
SG4352	TcCLB.506957.46	0.0802109428597266
SG1023	TcCLB.503919.60	0.0805393357802744
SG6790	TcCLB.511599.80	0.0805393357802744
SG7981	TcCLB.503519.30	0.0806740544363258
SG8944	TcCLB.507649.90	0.0811896361212344
SG6330	TcCLB.509399.170	0.081263464830252
SG7802	TcCLB.506419.69	0.0815361692877804
SG3137	TcCLB.511293.30	0.0815666288183038
SG8461	TcCLB.508479.370	0.0817541023155862
SG0776	TcCLB.510003.20	0.0820513376143436
SG1657	TcCLB.510435.10	0.0820513376143436
SG2103	TcCLB.506525.70	0.0820513376143436
SG3869	TcCLB.506577.20	0.0820513376143436
SG4618	TcCLB.511283.130	0.0820513376143436
SG4899	TcCLB.506753.230	0.0820513376143436
SG6783	TcCLB.511603.70	0.0820513376143436
SG3188	TcCLB.509777.130	0.0820718904730557
SG2054	TcCLB.507801.130	0.0821061058318357
SG3182	TcCLB.510879.80	0.0825807841711642
SG1214	TcCLB.503551.20	0.0826630454774646
SG2302	TcCLB.509207.90	0.0826630454774646
SG2983	TcCLB.506297.120	0.0826630454774646
SG5112	TcCLB.506363.110	0.0826630454774646

Supra gene	Representative ID	FDR
SG7519	TcCLB.504073.10	0.0826630454774646
SG0964	TcCLB.510655.120	0.082875784202173
SG3887	TcCLB.510667.80	0.0829370754586043
SG3985	TcCLB.503515.20	0.0829370754586043
SG4641	TcCLB.507765.20	0.0829370754586043
SG7409	TcCLB.504149.30	0.0829370754586043
SG2209	TcCLB.508657.10	0.0830236843559242
SG4845	TcCLB.510121.170	0.0830236843559242
SG4139	TcCLB.506297.320	0.0835044812896504
SG3603	TcCLB.510359.120	0.0835583784010318
SG3046	TcCLB.508831.20	0.0837374434153261
SG7557	TcCLB.506117.9	0.0837374434153261
SG1845	TcCLB.503939.120	0.0839067309096333
SG3983	TcCLB.506893.25	0.0839067309096333
SG6399	TcCLB.507765.120	0.0843541060563906
SG2096	TcCLB.510155.160	0.0843574194617031
SG6478	TcCLB.509937.20	0.0845026029221735
SG7508	TcCLB.508829.9	0.0849323838046342
SG1775	TcCLB.507601.80	0.0851532797456201
SG3397	TcCLB.510357.80	0.0852046294417074
SG1635	TcCLB.507003.60	0.0852461180488869
SG6117	TcCLB.508411.10	0.0852461180488869
SG7763	TcCLB.508461.84	0.0852461180488869
SG4411	TcCLB.506691.70	0.0854870520568208
SG5720	TcCLB.503823.110	0.0857366708190291
SG2327	TcCLB.506625.120	0.0859580220615919
SG5667	TcCLB.509607.60	0.0861175872332859
SG4577	TcCLB.510357.20	0.0861814141285745
SG7617	TcCLB.507041.55	0.0864308945177945
SG8111	TcCLB.508739.21	0.0864308945177945
SG8771	TcCLB.506977.40	0.0864308945177945
SG5059	TcCLB.505945.60	0.0869653433662406
SG6409	TcCLB.506337.30	0.0872564327619117
SG1376	TcCLB.507663.30	0.0874736534923135
SG2318	TcCLB.511031.20	0.0874736534923135
SG4254	TcCLB.504243.30	0.0874736534923135
SG4799	TcCLB.507073.49	0.0874736534923135
SG6472	TcCLB.507807.30	0.0874736534923135
SG3986	TcCLB.510719.80	0.0875991761007902
SG4568	TcCLB.511281.20	0.0875991761007902
SG2134	TcCLB.503693.20	0.0876907297646672
SG7238	TcCLB.509269.4	0.087965615293661
SG6892	TcCLB.509705.10	0.088200568409168
SG1122	TcCLB.504019.3	0.0882186570326387
SG1526	TcCLB.506885.300	0.0882186570326387
SG3754	TcCLB.505171.90	0.0882186570326387
SG6881	TcCLB.509247.60	0.0882700388880824
SG7690	TcCLB.511283.184	0.0883191278454308
SG4924	TcCLB.508851.160	0.0887890042320295
SG6278	TcCLB.509985.50	0.0887890042320295
SG7348	TcCLB.507099.110	0.0887890042320295
SG2146	TcCLB.506679.210	0.088817230692544
SG2461	TcCLB.511383.80	0.088817230692544

Supra gene	Representative ID	FDR
SG3479	TcCLB.507897.30	0.0890788218688853
SG4880	TcCLB.511277.486	0.0891986781514472
SG5708	TcCLB.511153.60	0.0893510423788064
SG6784	TcCLB.510621.49	0.0894214262224999
SG2631	TcCLB.503847.70	0.0894800244160935
SG1274	TcCLB.504213.90	0.0899745498224773
SG2132	TcCLB.508645.50	0.0902780441375908
SG4735	TcCLB.508263.30	0.0904869588558517
SG2404	TcCLB.508989.30	0.0910265190271366
SG6238	TcCLB.503809.75	0.0910326742028647
SG4003	TcCLB.506925.120	0.0912144534094899
SG2898	TcCLB.503843.20	0.0913128065183706
SG8914	TcCLB.508461.440	0.0913655089675605
SG6081	TcCLB.506175.100	0.0917665017078802
SG0731	TcCLB.506559.559	0.0919702185434925
SG1266	TcCLB.510889.10	0.0919702185434925
SG3148	TcCLB.510105.40	0.0919702185434925
SG6503	TcCLB.509287.60	0.0920762152284638
SG3221	TcCLB.508693.80	0.0922757766388034
SG6112	TcCLB.509103.39	0.0922757766388034
SG6886	TcCLB.504147.130	0.0926293464243335
SG6640	TcCLB.511491.150	0.0926396248460404
SG6292	TcCLB.507925.50	0.0936457902602019
SG7837	TcCLB.511283.200	0.0936457902602019
SG8709	TcCLB.511019.84	0.0936457902602019
SG3187	TcCLB.506855.110	0.0939287571535913
SG0343	TcCLB.457101.20	0.0944619371444065
SG4620	TcCLB.505999.180	0.0944619371444065
SG5530	TcCLB.509913.20	0.0944619371444065
SG6079	TcCLB.504741.250	0.0944619371444065
SG7435	TcCLB.510877.80	0.0944619371444065
SG0467	TcCLB.508141.70	0.0946533218414005
SG5998	TcCLB.503687.10	0.0952433093682981
SG7992	TcCLB.508461.200	0.0952433093682981
SG5887	TcCLB.510121.180	0.0953168018220343
SG3356	TcCLB.510303.60	0.0955626395053956
SG1239	TcCLB.508637.150	0.0957738556367985
SG1088	TcCLB.508995.69	0.0959157448812255
SG6584	TcCLB.510821.10	0.0959268851956784
SG0337	TcCLB.511825.40	0.096413735997904
SG5573	TcCLB.511389.140	0.0965041097527616
SG8805	TcCLB.507703.10	0.0968412421816028
SG7744	TcCLB.511827.20	0.0969373114421162
SG0397	TcCLB.509111.19	0.0971476938769457
SG3229	TcCLB.509893.20	0.0975058335964553
SG2301	TcCLB.506779.90	0.0976205716524699
SG6020	TcCLB.508965.39	0.0976205716524699
SG0472	TcCLB.506297.150	0.0977055207182715
SG3200	TcCLB.507007.40	0.0977055207182715
SG3916	TcCLB.508785.10	0.0977435208146532
SG3739	TcCLB.506975.63	0.0979654267935607
SG4857	TcCLB.507047.140	0.0979654267935607
SG8463	TcCLB.511809.30	0.0979654267935607

Supra gene	Representative ID	FDR
SG8360	TcCLB.507019.50	0.0980570925214038
SG4717	TcCLB.511239.49	0.098129696566785
SG6386	TcCLB.506753.110	0.098129696566785
SG6415	TcCLB.507049.50	0.098162733916502
SG5784	TcCLB.510835.24	0.0983006400639524
SG8096	TcCLB.508919.150	0.0983784829834126
SG0558	TcCLB.508525.60	0.0987137674212526
SG2372	TcCLB.507035.120	0.0987137674212526
SG4603	TcCLB.511257.30	0.0987137674212526
SG5021	TcCLB.510681.20	0.0987137674212526
SG5087	TcCLB.508277.230	0.0987908743226033
SG1996	TcCLB.508909.10	0.0990109723669413
SG2852	TcCLB.511277.115	0.0990262624725283
SG1784	TcCLB.504021.100	0.0991761486026337
SG2225	TcCLB.509999.50	0.0994545510740336
SG7355	TcCLB.511421.224	0.0994545510740336
SG3556	TcCLB.506529.230	0.0997247814368647
SG6616	TcCLB.506229.60	0.0997247814368647

*: complete statistical data and reads per kilobase million (RPKM) values can be obtained directly from the corresponding author.

TABLE V

List of differentially expressed genes (DEGs) (Representative ID from supra gene) with their respective expression cluster

Cluster	ID	Cluster	ID	Cluster	ID
1	TcCLB.510583.80	4	TcCLB.509201.15	9	TcCLB.510609.30
1	TcCLB.511835.10	4	TcCLB.510763.30	9	TcCLB.507801.114
1	TcCLB.507753.10	4	TcCLB.510603.100	9	TcCLB.506885.300
1	TcCLB.511643.110	4	TcCLB.504075.10	9	TcCLB.509023.140
1	TcCLB.510787.10	4	TcCLB.509063.30	9	TcCLB.506727.150
1	TcCLB.511593.40	4	TcCLB.509215.40	9	TcCLB.508355.114
1	TcCLB.506289.140	4	TcCLB.508823.50	9	TcCLB.506257.70
1	TcCLB.506551.10	4	TcCLB.508637.150	9	TcCLB.507017.130
1	TcCLB.511401.90	4	TcCLB.509065.100	9	TcCLB.510219.30
1	TcCLB.511325.40	4	TcCLB.508799.70	9	TcCLB.504167.40
1	TcCLB.506279.140	4	TcCLB.506195.90	9	TcCLB.506931.70
1	TcCLB.506989.300	4	TcCLB.508405.50	9	TcCLB.507011.110
1	TcCLB.511657.20	4	TcCLB.503555.40	9	TcCLB.508593.40
1	TcCLB.510581.9	4	TcCLB.509599.100	9	TcCLB.509453.20
1	TcCLB.511575.130	4	TcCLB.508501.190	9	TcCLB.506265.110
1	TcCLB.506153.10	4	TcCLB.511277.539	9	TcCLB.511511.20
1	TcCLB.509599.130	4	TcCLB.506581.10	9	TcCLB.503651.49
1	TcCLB.508499.39	4	TcCLB.508177.100	9	TcCLB.511003.90
1	TcCLB.511385.110	4	TcCLB.506443.70	9	TcCLB.508479.300
1	TcCLB.511253.31	4	TcCLB.509795.30	10	TcCLB.508625.160
1	TcCLB.508621.10	4	TcCLB.510667.60	10	TcCLB.506811.50
1	TcCLB.509237.120	4	TcCLB.510655.80	10	TcCLB.511727.14
1	TcCLB.504255.30	4	TcCLB.510659.44	10	TcCLB.511509.30
1	TcCLB.504053.10	4	TcCLB.424123.40	10	TcCLB.511269.50
1	TcCLB.506775.30	4	TcCLB.503449.14	10	TcCLB.511517.160
1	TcCLB.507659.20	4	TcCLB.510615.10	10	TcCLB.503525.4

Cluster	ID	Cluster	ID	Cluster	ID
1	TcCLB.504625.70	4	TcCLB.510943.30	10	TcCLB.509105.4
1	TcCLB.509029.40	4	TcCLB.510039.64	10	TcCLB.509023.90
1	TcCLB.511583.10	4	TcCLB.503735.40	10	TcCLB.506475.90
1	TcCLB.506989.30	4	TcCLB.507049.100	10	TcCLB.511151.54
1	TcCLB.510359.290	4	TcCLB.506835.40	10	TcCLB.508741.340
1	TcCLB.510729.230	4	TcCLB.509539.44	10	TcCLB.503415.40
1	TcCLB.509429.160	4	TcCLB.509055.50	10	TcCLB.509937.130
1	TcCLB.504105.160	4	TcCLB.508405.90	10	TcCLB.506905.30
1	TcCLB.511907.30	4	TcCLB.508859.80	10	TcCLB.506009.40
1	TcCLB.510155.10	4	TcCLB.511281.40	10	TcCLB.511289.90
1	TcCLB.507817.80	4	TcCLB.504001.20	10	TcCLB.510691.10
1	TcCLB.503465.40	4	TcCLB.509561.70	10	TcCLB.509769.30
1	TcCLB.508909.10	4	TcCLB.510661.244	10	TcCLB.509683.80
1	TcCLB.503879.110	4	TcCLB.503923.30	10	TcCLB.510657.20
1	TcCLB.510735.40	4	TcCLB.506181.90	10	TcCLB.510425.28
1	TcCLB.510155.160	4	TcCLB.506583.30	10	TcCLB.511277.140
1	TcCLB.509429.280	4	TcCLB.508153.820	10	TcCLB.508231.60
1	TcCLB.507049.60	4	TcCLB.506355.130	10	TcCLB.509331.150
1	TcCLB.509207.80	4	TcCLB.506443.40	10	TcCLB.510045.20
1	TcCLB.504003.30	4	TcCLB.510993.10	10	TcCLB.503917.7
1	TcCLB.506825.100	4	TcCLB.511653.40	10	TcCLB.511003.200
1	TcCLB.421619.10	4	TcCLB.503521.60	10	TcCLB.511867.130
1	TcCLB.503595.10	4	TcCLB.504129.40	10	TcCLB.504147.190
1	TcCLB.506175.50	4	TcCLB.510395.20	10	TcCLB.504149.100
1	TcCLB.508973.60	4	TcCLB.508593.30	10	TcCLB.511807.130
1	TcCLB.510409.20	4	TcCLB.506221.120	10	TcCLB.511725.170
1	TcCLB.508269.40	4	TcCLB.506195.300	10	TcCLB.508613.10
1	TcCLB.508917.20	4	TcCLB.504085.20	11	TcCLB.511911.90
1	TcCLB.507275.40	4	TcCLB.511821.110	11	TcCLB.509239.10
1	TcCLB.511761.50	4	TcCLB.511807.64	11	TcCLB.509639.10
1	TcCLB.511127.340	4	TcCLB.508981.20	11	TcCLB.506201.70
1	TcCLB.511903.170	4	TcCLB.511499.59	11	TcCLB.506107.10
1	TcCLB.508919.94	4	TcCLB.506289.60	11	TcCLB.509999.120
1	TcCLB.509879.40	4	TcCLB.510349.20	11	TcCLB.503571.19
1	TcCLB.509713.30	4	TcCLB.507159.14	11	TcCLB.458241.10
1	TcCLB.506999.180	4	TcCLB.510665.30	11	TcCLB.507009.30
1	TcCLB.506301.20	4	TcCLB.509749.30	11	TcCLB.506559.129
1	TcCLB.506825.10	4	TcCLB.509717.34	11	TcCLB.511303.40
1	TcCLB.510155.94	4	TcCLB.510303.60	11	TcCLB.507715.10
1	TcCLB.510339.90	4	TcCLB.511635.30	11	TcCLB.509049.30
1	TcCLB.510877.170	4	TcCLB.509599.120	11	TcCLB.511821.170
1	TcCLB.504021.74	4	TcCLB.506529.50	11	TcCLB.508065.70
1	TcCLB.511285.40	4	TcCLB.511181.20	11	TcCLB.506851.10
1	TcCLB.503747.40	4	TcCLB.508153.454	11	TcCLB.510145.10
1	TcCLB.508153.270	4	TcCLB.511903.134	11	TcCLB.509237.90
1	TcCLB.511277.100	4	TcCLB.507023.50	11	TcCLB.504199.10
1	TcCLB.504243.40	4	TcCLB.508817.10	11	TcCLB.507811.30
1	TcCLB.509791.60	4	TcCLB.509179.80	11	TcCLB.508645.40
1	TcCLB.510829.10	4	TcCLB.510397.10	11	TcCLB.510007.50
1	TcCLB.508891.30	4	TcCLB.507711.50	11	TcCLB.506009.10
1	TcCLB.511181.80	4	TcCLB.511031.40	11	TcCLB.509835.10
1	TcCLB.506263.40	4	TcCLB.509509.50	11	TcCLB.503975.50
1	TcCLB.503945.40	4	TcCLB.508547.140	11	TcCLB.511857.10

Cluster	ID	Cluster	ID	Cluster	ID
1	TcCLB.511165.100	4	TcCLB.507007.74	11	TcCLB.509573.10
1	TcCLB.510659.270	4	TcCLB.508577.140	11	TcCLB.506959.70
1	TcCLB.507765.20	4	TcCLB.507857.10	11	TcCLB.509717.60
1	TcCLB.509537.50	4	TcCLB.507951.140	11	TcCLB.509001.20
1	TcCLB.511431.54	4	TcCLB.506627.110	11	TcCLB.509053.30
1	TcCLB.505071.140	4	TcCLB.510329.330	11	TcCLB.511865.40
1	TcCLB.508823.20	4	TcCLB.503925.90	11	TcCLB.508443.30
1	TcCLB.511017.25	4	TcCLB.509237.50	11	TcCLB.508999.260
1	TcCLB.507943.60	4	TcCLB.506777.20	11	TcCLB.508999.250
1	TcCLB.506775.180	4	TcCLB.508231.200	11	TcCLB.509937.220
1	TcCLB.508943.20	4	TcCLB.507017.70	11	TcCLB.511761.10
1	TcCLB.509569.40	4	TcCLB.506975.20	11	TcCLB.506477.60
1	TcCLB.506735.40	4	TcCLB.510065.40	11	TcCLB.509937.160
1	TcCLB.509151.90	4	TcCLB.508153.620	11	TcCLB.507143.60
1	TcCLB.507491.70	4	TcCLB.509045.20	11	TcCLB.508153.260
1	TcCLB.506181.10	4	TcCLB.511209.60	11	TcCLB.507241.30
1	TcCLB.510007.20	4	TcCLB.506435.110	11	TcCLB.509109.160
1	TcCLB.511181.60	4	TcCLB.503829.10	11	TcCLB.507515.90
1	TcCLB.467287.10	4	TcCLB.504069.90	11	TcCLB.503939.50
1	TcCLB.503943.14	4	TcCLB.508577.80	11	TcCLB.509563.10
1	TcCLB.503975.100	4	TcCLB.510667.10	11	TcCLB.504125.100
1	TcCLB.510575.210	4	TcCLB.509715.40	11	TcCLB.504137.50
1	TcCLB.508641.180	4	TcCLB.511389.130	11	TcCLB.503999.100
1	TcCLB.510719.120	4	TcCLB.509065.140	11	TcCLB.508593.130
1	TcCLB.503955.100	4	TcCLB.509161.20	11	TcCLB.503409.10
1	TcCLB.511153.70	4	TcCLB.510339.54	11	TcCLB.511383.80
1	TcCLB.506229.64	4	TcCLB.507519.164	11	TcCLB.503465.10
1	TcCLB.509733.70	4	TcCLB.508737.150	11	TcCLB.509099.130
1	TcCLB.508153.520	4	TcCLB.511819.30	11	TcCLB.506797.110
1	TcCLB.507063.130	4	TcCLB.510421.270	11	TcCLB.506573.50
1	TcCLB.506871.180	4	TcCLB.508153.920	11	TcCLB.508661.70
1	TcCLB.509997.60	4	TcCLB.503995.50	11	TcCLB.509607.70
1	TcCLB.508277.340	4	TcCLB.507061.34	11	TcCLB.511127.180
1	TcCLB.511633.79	4	TcCLB.503929.20	11	TcCLB.506773.90
1	TcCLB.508983.20	4	TcCLB.508175.90	11	TcCLB.509695.100
1	TcCLB.504117.50	4	TcCLB.509789.40	11	TcCLB.508409.160
1	TcCLB.504213.100	4	TcCLB.507017.64	11	TcCLB.509023.50
1	TcCLB.511189.70	4	TcCLB.508153.164	11	TcCLB.506399.80
1	TcCLB.506285.30	4	TcCLB.511353.50	11	TcCLB.503975.70
1	TcCLB.510599.70	4	TcCLB.508817.140	11	TcCLB.506691.60
1	TcCLB.445635.10	4	TcCLB.508661.10	11	TcCLB.509229.60
1	TcCLB.506925.310	4	TcCLB.511719.30	11	TcCLB.508669.10
1	TcCLB.509703.10	4	TcCLB.509859.50	11	TcCLB.511857.50
1	TcCLB.503865.60	4	TcCLB.508153.1140	11	TcCLB.504199.20
1	TcCLB.507629.39	4	TcCLB.511635.40	11	TcCLB.511759.40
1	TcCLB.427247.10	4	TcCLB.509337.19	11	TcCLB.508231.190
1	TcCLB.510901.170	4	TcCLB.507787.140	11	TcCLB.510575.20
1	TcCLB.511909.30	4	TcCLB.510359.170	11	TcCLB.510581.20
1	TcCLB.503461.20	4	TcCLB.506925.390	11	TcCLB.507963.10
1	TcCLB.507949.250	4	TcCLB.407335.9	11	TcCLB.508257.30
1	TcCLB.508461.240	4	TcCLB.507019.83	11	TcCLB.511649.100
1	TcCLB.506985.50	4	TcCLB.507251.10	11	TcCLB.511481.70
1	TcCLB.510901.230	4	TcCLB.506925.510	11	TcCLB.509937.50

Cluster	ID	Cluster	ID	Cluster	ID
1	TcCLB.506335.130	4	TcCLB.508307.170	11	TcCLB.508817.110
1	TcCLB.503413.4	4	TcCLB.510761.30	11	TcCLB.511287.130
1	TcCLB.511367.199	4	TcCLB.510431.230	11	TcCLB.508257.70
1	TcCLB.511367.170	4	TcCLB.508307.150	11	TcCLB.506681.40
1	TcCLB.506247.190	4	TcCLB.509895.20	11	TcCLB.506491.10
1	TcCLB.508667.40	4	TcCLB.507641.140	11	TcCLB.511295.60
1	TcCLB.506679.20	4	TcCLB.504147.260	11	TcCLB.507031.140
1	TcCLB.511367.240	4	TcCLB.508837.160	11	TcCLB.509003.40
1	TcCLB.511277.180	4	TcCLB.506625.70	11	TcCLB.506791.10
1	TcCLB.509907.60	4	TcCLB.506925.540	11	TcCLB.509003.30
2	TcCLB.509065.60	4	TcCLB.508813.80	11	TcCLB.511025.80
2	TcCLB.510663.19	4	TcCLB.510187.250	11	TcCLB.511167.60
2	TcCLB.510719.20	4	TcCLB.510187.280	11	TcCLB.506963.70
2	TcCLB.510003.20	4	TcCLB.506977.80	11	TcCLB.511511.150
2	TcCLB.506629.220	4	TcCLB.508173.70	11	TcCLB.509055.44
2	TcCLB.503531.40	4	TcCLB.508173.100	11	TcCLB.509109.30
2	TcCLB.506743.4	4	TcCLB.508547.50	11	TcCLB.509029.80
2	TcCLB.504069.56	4	TcCLB.510873.10	11	TcCLB.508461.40
2	TcCLB.508901.10	4	TcCLB.506627.100	11	TcCLB.510143.120
2	TcCLB.511899.40	4	TcCLB.510901.30	11	TcCLB.503559.70
2	TcCLB.506201.110	4	TcCLB.508153.184	11	TcCLB.510301.30
2	TcCLB.510099.120	4	TcCLB.508479.350	11	TcCLB.504253.40
2	TcCLB.504949.30	4	TcCLB.510187.480	11	TcCLB.503703.30
2	TcCLB.510647.30	4	TcCLB.510687.120	11	TcCLB.511517.30
2	TcCLB.504003.70	4	TcCLB.508547.190	11	TcCLB.508273.30
2	TcCLB.506727.140	4	TcCLB.508479.274	11	TcCLB.506709.10
2	TcCLB.505183.30	4	TcCLB.510689.30	11	TcCLB.506009.20
2	TcCLB.509943.30	4	TcCLB.507019.30	11	TcCLB.507609.40
2	TcCLB.509693.80	4	TcCLB.511907.270	11	TcCLB.510423.50
2	TcCLB.511903.45	4	TcCLB.508257.180	11	TcCLB.503823.120
2	TcCLB.504021.100	4	TcCLB.510687.70	11	TcCLB.427789.20
2	TcCLB.509551.70	4	TcCLB.511727.140	11	TcCLB.503795.10
2	TcCLB.510285.20	4	TcCLB.504109.140	11	TcCLB.507853.10
2	TcCLB.507057.40	4	TcCLB.510087.100	11	TcCLB.508601.90
2	TcCLB.507583.20	4	TcCLB.511725.174	11	TcCLB.510823.30
2	TcCLB.507895.140	4	TcCLB.510187.380	11	TcCLB.506479.120
2	TcCLB.509505.40	4	TcCLB.508173.180	11	TcCLB.506871.70
2	TcCLB.511025.70	4	TcCLB.507019.60	11	TcCLB.504073.20
2	TcCLB.507801.130	4	TcCLB.511249.44	11	TcCLB.511407.40
2	TcCLB.504431.30	5	TcCLB.510571.30	11	TcCLB.510507.50
2	TcCLB.509053.70	5	TcCLB.509921.51	11	TcCLB.511517.164
2	TcCLB.506581.30	5	TcCLB.508823.10	11	TcCLB.507305.40
2	TcCLB.507659.10	5	TcCLB.508851.200	11	TcCLB.507737.20
2	TcCLB.509207.90	5	TcCLB.506579.10	11	TcCLB.506477.20
2	TcCLB.506211.250	5	TcCLB.509643.100	11	TcCLB.506503.4
2	TcCLB.508269.60	5	TcCLB.503773.24	11	TcCLB.504087.10
2	TcCLB.511529.68	5	TcCLB.511229.50	11	TcCLB.511825.240
2	TcCLB.508905.30	5	TcCLB.509059.50	11	TcCLB.511257.30
2	TcCLB.506985.40	5	TcCLB.508153.1100	11	TcCLB.506789.270
2	TcCLB.509499.4	5	TcCLB.508689.20	11	TcCLB.511389.110
2	TcCLB.507251.20	5	TcCLB.506295.130	11	TcCLB.511167.110
2	TcCLB.510149.140	5	TcCLB.503687.30	11	TcCLB.507711.90
2	TcCLB.510421.140	5	TcCLB.507831.60	11	TcCLB.508263.30

Cluster	ID	Cluster	ID	Cluster	ID
2	TcCLB.505171.60	5	TcCLB.508707.160	11	TcCLB.508777.40
2	TcCLB.509033.80	5	TcCLB.506629.20	11	TcCLB.506727.110
2	TcCLB.509859.40	5	TcCLB.509267.40	11	TcCLB.506821.80
2	TcCLB.511321.60	5	TcCLB.503625.10	11	TcCLB.509463.10
2	TcCLB.505999.70	5	TcCLB.508577.130	11	TcCLB.508851.160
2	TcCLB.511815.40	5	TcCLB.511847.50	11	TcCLB.503617.31
2	TcCLB.508153.640	5	TcCLB.508625.130	11	TcCLB.508445.90
2	TcCLB.511239.110	5	TcCLB.511289.70	11	TcCLB.503411.10
2	TcCLB.511151.20	5	TcCLB.511383.50	11	TcCLB.505945.60
2	TcCLB.507057.30	5	TcCLB.505183.84	11	TcCLB.511165.80
2	TcCLB.510357.80	5	TcCLB.508999.40	11	TcCLB.506991.10
2	TcCLB.510445.50	5	TcCLB.511257.10	11	TcCLB.403875.10
2	TcCLB.508909.20	5	TcCLB.511353.10	11	TcCLB.508799.150
2	TcCLB.510001.10	5	TcCLB.506925.190	11	TcCLB.508507.40
2	TcCLB.509717.100	5	TcCLB.509831.33	11	TcCLB.508707.10
2	TcCLB.510899.50	5	TcCLB.507105.50	11	TcCLB.509859.30
2	TcCLB.506529.30	5	TcCLB.506925.14	11	TcCLB.506305.29
2	TcCLB.506821.110	5	TcCLB.508173.264	11	TcCLB.508387.100
2	TcCLB.510667.80	5	TcCLB.508173.40	11	TcCLB.506025.40
2	TcCLB.507031.29	5	TcCLB.504131.60	11	TcCLB.510421.180
2	TcCLB.511529.170	5	TcCLB.506629.40	11	TcCLB.506543.10
2	TcCLB.511287.90	5	TcCLB.510821.60	11	TcCLB.510735.90
2	TcCLB.508175.39	5	TcCLB.506321.280	11	TcCLB.507677.150
2	TcCLB.509499.10	5	TcCLB.511725.250	11	TcCLB.504229.110
2	TcCLB.510655.130	6	TcCLB.506435.370	11	TcCLB.503571.10
2	TcCLB.508741.170	6	TcCLB.505699.10	11	TcCLB.511285.30
2	TcCLB.505983.20	6	TcCLB.504015.30	11	TcCLB.511653.20
2	TcCLB.508699.40	6	TcCLB.506499.105	11	TcCLB.508965.39
2	TcCLB.511529.160	6	TcCLB.510795.10	11	TcCLB.509695.50
2	TcCLB.510031.70	6	TcCLB.506355.100	11	TcCLB.510663.40
2	TcCLB.503465.30	6	TcCLB.510065.10	11	TcCLB.504741.250
2	TcCLB.507017.40	6	TcCLB.506789.334	11	TcCLB.509229.10
2	TcCLB.509051.40	6	TcCLB.508671.20	11	TcCLB.509791.30
2	TcCLB.506413.70	6	TcCLB.506469.80	11	TcCLB.510305.70
2	TcCLB.511761.60	6	TcCLB.504223.20	11	TcCLB.509179.130
2	TcCLB.503681.20	6	TcCLB.511757.70	11	TcCLB.510729.140
2	TcCLB.507951.170	6	TcCLB.505999.10	11	TcCLB.504021.20
2	TcCLB.510155.20	6	TcCLB.504191.10	11	TcCLB.511903.90
2	TcCLB.510257.24	6	TcCLB.508015.10	11	TcCLB.507089.30
2	TcCLB.506195.100	6	TcCLB.507951.299	11	TcCLB.509775.9
2	TcCLB.503891.40	6	TcCLB.508909.330	11	TcCLB.511545.140
2	TcCLB.509471.30	6	TcCLB.508461.80	11	TcCLB.506241.80
2	TcCLB.504013.55	6	TcCLB.485683.10	11	TcCLB.506297.350
2	TcCLB.508351.40	6	TcCLB.506405.140	11	TcCLB.511323.60
2	TcCLB.510091.100	6	TcCLB.504019.3	11	TcCLB.508211.50
2	TcCLB.508409.199	6	TcCLB.506825.70	11	TcCLB.509999.140
2	TcCLB.511459.50	6	TcCLB.509601.140	11	TcCLB.506559.524
2	TcCLB.511823.70	6	TcCLB.504173.30	11	TcCLB.506529.400
2	TcCLB.511545.170	6	TcCLB.511217.120	11	TcCLB.506529.595
2	TcCLB.508501.250	6	TcCLB.506825.200	11	TcCLB.511545.80
2	TcCLB.508543.120	6	TcCLB.503565.29	11	TcCLB.506925.10
2	TcCLB.511825.20	6	TcCLB.503515.14	11	TcCLB.503955.80
2	TcCLB.506821.90	6	TcCLB.511647.10	11	TcCLB.506289.80

Cluster	ID	Cluster	ID	Cluster	ID
2	TcCLB.509643.70	6	TcCLB.504149.175	11	TcCLB.504147.240
2	TcCLB.511211.210	6	TcCLB.510149.120	11	TcCLB.504147.110
2	TcCLB.506503.60	6	TcCLB.508399.10	11	TcCLB.504125.60
2	TcCLB.510357.90	6	TcCLB.504153.30	11	TcCLB.504423.15
2	TcCLB.511033.20	6	TcCLB.505071.90	11	TcCLB.510297.119
2	TcCLB.507003.50	6	TcCLB.503943.20	11	TcCLB.503975.40
2	TcCLB.507081.130	6	TcCLB.511805.20	11	TcCLB.506625.230
2	TcCLB.509937.20	6	TcCLB.508193.60	11	TcCLB.508257.220
2	TcCLB.509179.70	6	TcCLB.509901.100	11	TcCLB.511389.80
2	TcCLB.510859.10	6	TcCLB.507851.70	11	TcCLB.507019.20
2	TcCLB.503925.80	6	TcCLB.506625.120	11	TcCLB.506577.5
2	TcCLB.506945.240	6	TcCLB.506195.290	11	TcCLB.506175.120
2	TcCLB.482097.20	6	TcCLB.509683.20	11	TcCLB.509647.50
2	TcCLB.507641.50	6	TcCLB.507081.110	11	TcCLB.510129.20
2	TcCLB.511215.50	6	TcCLB.511593.20	11	TcCLB.506679.80
2	TcCLB.506795.70	6	TcCLB.509755.50	11	TcCLB.506619.30
2	TcCLB.508813.60	6	TcCLB.511517.130	11	TcCLB.511295.20
2	TcCLB.506945.200	6	TcCLB.506755.50	11	TcCLB.510889.100
2	TcCLB.510859.17	6	TcCLB.507519.150	11	TcCLB.511391.120
2	TcCLB.507099.30	6	TcCLB.511277.60	11	TcCLB.510329.210
2	TcCLB.506553.20	6	TcCLB.509109.140	11	TcCLB.510303.200
2	TcCLB.511421.210	6	TcCLB.506475.113	11	TcCLB.511019.80
2	TcCLB.503419.50	6	TcCLB.506241.160	11	TcCLB.511727.210
2	TcCLB.511807.150	6	TcCLB.509937.170	11	TcCLB.508879.160
2	TcCLB.511367.60	6	TcCLB.509161.10	11	TcCLB.506559.434
2	TcCLB.508971.30	6	TcCLB.508307.180	11	TcCLB.510329.70
2	TcCLB.508409.110	6	TcCLB.506315.80	12	TcCLB.510275.255
2	TcCLB.511367.138	6	TcCLB.508111.30	12	TcCLB.507059.60
2	TcCLB.506247.50	6	TcCLB.503617.10	12	TcCLB.504797.140
2	TcCLB.508799.240	6	TcCLB.506303.60	12	TcCLB.508153.1050
2	TcCLB.504071.60	6	TcCLB.511759.30	12	TcCLB.508821.50
2	TcCLB.508815.120	6	TcCLB.511827.100	12	TcCLB.506357.130
2	TcCLB.511491.70	6	TcCLB.511909.70	12	TcCLB.508961.20
2	TcCLB.508799.10	6	TcCLB.506193.60	12	TcCLB.504113.30
2	TcCLB.507209.10	6	TcCLB.510335.10	12	TcCLB.508153.1110
2	TcCLB.508461.410	6	TcCLB.508737.70	12	TcCLB.509601.20
2	TcCLB.509105.90	6	TcCLB.510333.10	12	TcCLB.511649.94
3	TcCLB.503731.9	6	TcCLB.503697.70	12	TcCLB.506739.200
3	TcCLB.506871.20	6	TcCLB.509599.60	12	TcCLB.509945.24
3	TcCLB.504023.10	6	TcCLB.510667.120	12	TcCLB.507951.70
3	TcCLB.506287.80	6	TcCLB.510303.50	12	TcCLB.511353.69
3	TcCLB.508411.50	6	TcCLB.503459.10	12	TcCLB.507031.64
3	TcCLB.504051.40	6	TcCLB.507775.10	12	TcCLB.509167.20
3	TcCLB.509617.40	6	TcCLB.503431.90	12	TcCLB.508637.120
3	TcCLB.507669.110	6	TcCLB.506989.10	12	TcCLB.509631.160
3	TcCLB.507715.20	6	TcCLB.402863.9	12	TcCLB.509717.30
3	TcCLB.508707.130	6	TcCLB.510357.10	12	TcCLB.511529.50
3	TcCLB.509207.120	6	TcCLB.503653.60	12	TcCLB.510797.20
3	TcCLB.507021.50	6	TcCLB.511411.16	12	TcCLB.511355.30
3	TcCLB.510101.480	6	TcCLB.506999.120	12	TcCLB.503987.39
3	TcCLB.509213.110	6	TcCLB.508965.70	12	TcCLB.510443.30
3	TcCLB.507093.40	6	TcCLB.506123.30	12	TcCLB.511021.90
3	TcCLB.511307.20	6	TcCLB.507031.90	12	TcCLB.506975.63

Cluster	ID	Cluster	ID	Cluster	ID
3	TcCLB.509595.30	6	TcCLB.511249.110	12	TcCLB.507049.20
3	TcCLB.510689.80	6	TcCLB.506229.10	12	TcCLB.506553.10
3	TcCLB.506401.280	6	TcCLB.503939.20	12	TcCLB.508641.184
3	TcCLB.511167.20	6	TcCLB.508989.110	12	TcCLB.506175.10
3	TcCLB.506425.80	6	TcCLB.510155.50	12	TcCLB.508613.30
3	TcCLB.508741.380	6	TcCLB.511559.50	12	TcCLB.510691.70
3	TcCLB.510155.115	6	TcCLB.507793.20	12	TcCLB.508717.36
3	TcCLB.506855.130	6	TcCLB.503599.10	12	TcCLB.507913.20
3	TcCLB.511907.240	6	TcCLB.503781.30	12	TcCLB.507047.140
3	TcCLB.510225.40	6	TcCLB.510877.140	12	TcCLB.511283.124
3	TcCLB.506825.80	6	TcCLB.504427.50	12	TcCLB.511361.10
3	TcCLB.510887.30	6	TcCLB.511165.40	12	TcCLB.503579.40
3	TcCLB.508857.140	6	TcCLB.508461.310	12	TcCLB.509229.130
3	TcCLB.508831.66	6	TcCLB.510339.100	12	TcCLB.509109.114
3	TcCLB.509429.130	6	TcCLB.506635.139	12	TcCLB.504161.50
3	TcCLB.509791.90	6	TcCLB.505073.10	12	TcCLB.506363.110
3	TcCLB.503923.10	6	TcCLB.509007.50	12	TcCLB.511729.60
3	TcCLB.510861.70	6	TcCLB.441241.10	12	TcCLB.507007.45
3	TcCLB.504125.64	6	TcCLB.504153.140	12	TcCLB.509429.120
3	TcCLB.511289.50	6	TcCLB.506227.150	12	TcCLB.511745.60
3	TcCLB.508153.680	6	TcCLB.506625.190	12	TcCLB.509319.20
3	TcCLB.510517.60	6	TcCLB.511439.70	12	TcCLB.506713.14
3	TcCLB.504255.20	6	TcCLB.505683.5	12	TcCLB.506123.24
3	TcCLB.510099.30	6	TcCLB.511211.70	12	TcCLB.505997.200
3	TcCLB.510311.30	6	TcCLB.510977.9	12	TcCLB.436521.9
3	TcCLB.507491.119	6	TcCLB.511391.110	12	TcCLB.510323.60
3	TcCLB.506327.80	6	TcCLB.506993.160	12	TcCLB.511903.20
3	TcCLB.509835.30	6	TcCLB.506679.70	12	TcCLB.506855.350
3	TcCLB.509799.20	6	TcCLB.507681.200	12	TcCLB.503809.75
3	TcCLB.510421.310	6	TcCLB.511491.40	12	TcCLB.509799.60
3	TcCLB.511253.20	6	TcCLB.508017.30	12	TcCLB.504797.80
3	TcCLB.507515.120	6	TcCLB.511003.160	12	TcCLB.508273.70
3	TcCLB.508207.250	6	TcCLB.511807.125	12	TcCLB.509253.30
3	TcCLB.508917.30	6	TcCLB.509003.70	12	TcCLB.506275.20
3	TcCLB.506593.40	6	TcCLB.511367.210	12	TcCLB.506297.140
3	TcCLB.510221.20	6	TcCLB.511809.10	12	TcCLB.511649.150
3	TcCLB.510661.190	6	TcCLB.504071.110	12	TcCLB.510879.180
3	TcCLB.504111.10	6	TcCLB.509527.120	12	TcCLB.511421.170
3	TcCLB.508741.370	6	TcCLB.511807.190	12	TcCLB.510091.110
3	TcCLB.507143.80	6	TcCLB.508863.10	12	TcCLB.510743.10
3	TcCLB.503733.30	6	TcCLB.511259.50	12	TcCLB.508647.174
3	TcCLB.507023.180	6	TcCLB.511367.159	12	TcCLB.506411.25
3	TcCLB.511001.70	6	TcCLB.509647.180	12	TcCLB.508687.25
3	TcCLB.503715.40	6	TcCLB.511317.50	12	TcCLB.506977.60
3	TcCLB.511465.20	6	TcCLB.511885.50	12	TcCLB.511283.184
3	TcCLB.511159.14	6	TcCLB.506317.10	12	TcCLB.508479.310
3	TcCLB.510311.100	6	TcCLB.511511.5	12	TcCLB.509105.130
3	TcCLB.506841.20	7	TcCLB.504125.80	12	TcCLB.510187.290
3	TcCLB.509791.100	7	TcCLB.508909.300	12	TcCLB.507053.170
3	TcCLB.508637.160	7	TcCLB.508707.50	12	TcCLB.506445.100
3	TcCLB.504153.260	7	TcCLB.508593.100	12	TcCLB.508823.60
3	TcCLB.509705.10	7	TcCLB.509099.150	12	TcCLB.504071.40
3	TcCLB.510431.140	7	TcCLB.506195.150	12	TcCLB.508707.200

Cluster	ID	Cluster	ID	Cluster	ID
3	TcCLB.507093.160	7	TcCLB.509777.60	12	TcCLB.511725.134
3	TcCLB.509013.10	7	TcCLB.509671.160	12	TcCLB.508479.370
3	TcCLB.506885.90	7	TcCLB.510305.40	12	TcCLB.511655.20
3	TcCLB.510513.70	7	TcCLB.503679.30	12	TcCLB.507053.20
3	TcCLB.509267.20	7	TcCLB.507317.20	12	TcCLB.510799.60
3	TcCLB.503479.70	7	TcCLB.507927.80	12	TcCLB.503603.10
3	TcCLB.504057.130	7	TcCLB.506885.60	12	TcCLB.506247.40
3	TcCLB.510901.180	7	TcCLB.510733.50		
3	TcCLB.508827.110	7	TcCLB.511317.70		
3	TcCLB.506791.20	7	TcCLB.506419.10		
3	TcCLB.509857.40	8	TcCLB.510217.10		
3	TcCLB.511511.3	8	TcCLB.510783.20		
3	TcCLB.507073.30	8	TcCLB.506375.100		
3	TcCLB.508461.350	8	TcCLB.507485.100		
3	TcCLB.511727.230	8	TcCLB.509799.50		
3	TcCLB.511283.240	8	TcCLB.510039.80		
3	TcCLB.511725.230	8	TcCLB.503891.120		
3	TcCLB.510187.400	8	TcCLB.509137.10		
3	TcCLB.508871.140	8	TcCLB.503955.89		
		8	TcCLB.508727.24		
		8	TcCLB.511003.30		
		8	TcCLB.507053.209		
		8	TcCLB.506559.550		

TABLE VI

Summary of analysis of expression of RNA-binding proteins (RBPs) partners in *Trypanosoma cruzi* growth curve

							False discovery rates (FDR) 5%				
RBP	Supra gene	Representative ID	DEG (present data)	Parasite stage	TcCLB partners (from literature)	Non-redundant patners (present work supergenes)	Non DEGs (%)	DEGs in both (%)	DEGs in total RNA only (%)	DEGs in P/G RNA only (%)	Total (%)
Dhh1	SG2469	TcCLB.510997.50	No	Epi	204	128	90	4,69	1,56	3,13	9,38
EF-1α	SG0113	TcCLB.511367.360	No	Epi	85	77	80	2,60	6,49	11,69	20,78
EF-1α	SG0113	TcCLB.511367.360	No	Epi under nutritional stress	133	125	60	16,80	15,20	8,00	40,00
NRBD1	SG6635	TcCLB.511727.270	No	Epi	142	139	75	12,23	7,19	5,04	24,46
NRBD1	SG6635	TcCLB.511727.270	No	Epi under nutritional stress	54	54	75	5,56	9,26	9,26	24,07
Puf6	SG5973	TcCLB.510125.10	Yes (pol)	Epi	8	8	50	0,00	12,50	37,50	50,00
RBP40	SG2592	TcCLB.511837.129	No	Epi	148	145	74	4,14	13,10	8,28	25,52
ZC3H39	SG1319	TcCLB.508895.50	No	Epi	49	46	84	2,17	6,52	6,52	15,22
ZC3H39	SG1319	TcCLB.508895.50	No	Epi under nutritional stress	199	188	70	10,64	14,36	4,79	29,79
ZFP2	SG4272	TcCLB.503989.10	No	Epi	228	222	73	2,70	16,22	7,66	26,58

DEGs: differentially expressed genes; P/G: polysomal/granular.

Expression pattern of RBPs partners in T. cruzi growth curve

Groups of mRNAs associated with *Trypanosoma cruzi* RNA-binding proteins (RBPs) were retrieved from literature search: TcDHH1 (Holetz et al. 2010), TcEF-1α (Alves et al. 2015), TcNRBD1 (Oliveira et al. 2016), TcPUF6 (Dallagiovanna et al. 2008), TcZC3H39 (Alves et al. 2014); ZFP2 (Mörking et al. 2012). For each RBP, partners IDs were crossed with present *T. cruzi* growth curve RNA-Seq supra genes (SGs) data using CL Brener representative IDs. RBPs partners differentially expressed genes (DEGs) in the present data [false discovery rates (FDR) threshold of 0.05] were visualised by heat maps after Hierarchical clustering performed in MultiExperimentViewer (MeV) version 4.8.1, using the Euclidean distance and average linkage clustering metrics. As can be seen in Supplementary data (Table VI), only TcPUF6 mRNA was found modulated in our data. However, a percentage of these RBPs partners were modulated (minimum of 9,4% of Dhh1 partners and maximum of 50% of PUF6 partners). The expression pattern of DEGs are presented in Supplementary data (Figs 1-10).

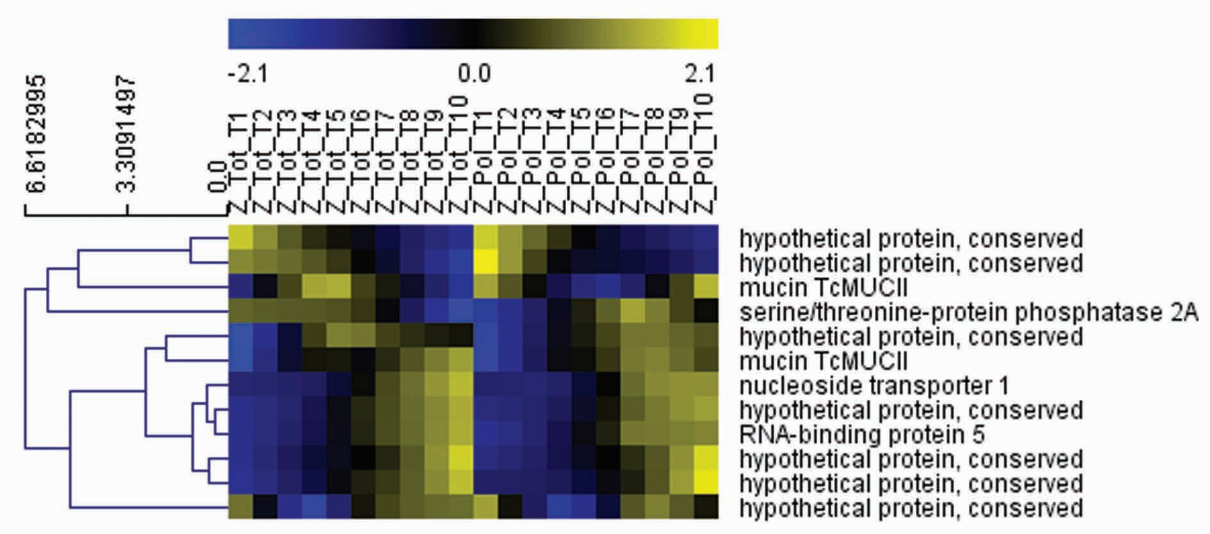

Fig. 1: Dhh1 (epimastigote). Heat map of Dhh1 partners differentially expressed genes (DEGs) (epimastigote), representing total (Tot) and polysomal/granular (P/G) modulation across the time points of the growth curve. Grades of blue and yellow represent decrease or increase, respectively, of log2-ratio time points against day 1 of the growth curve.

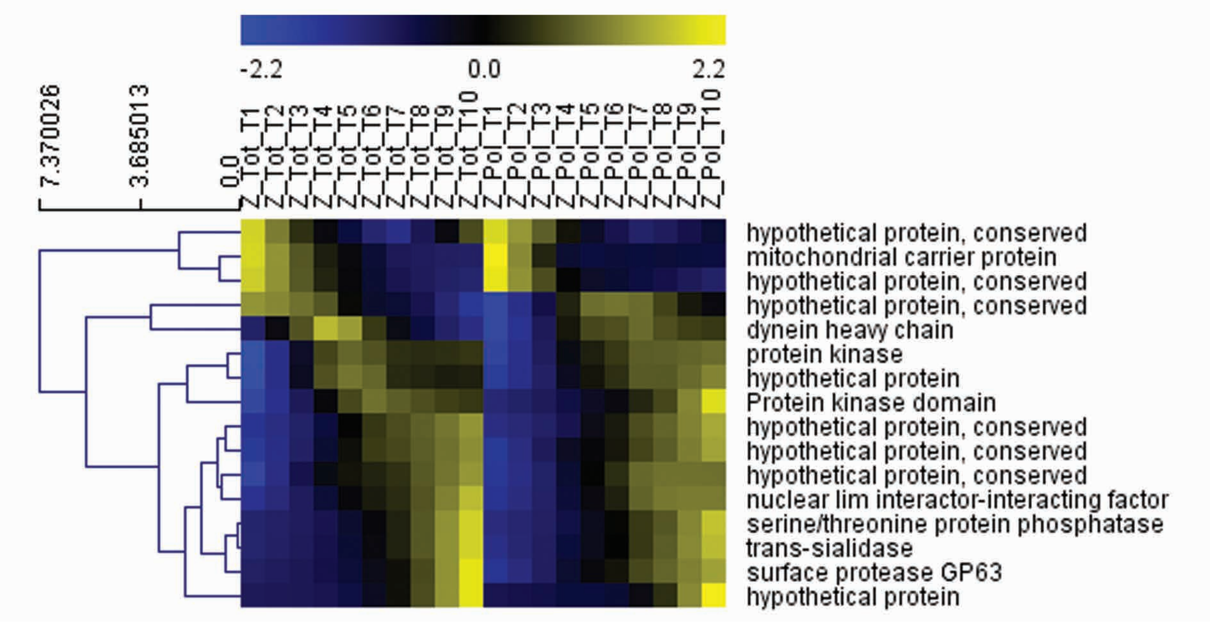

Fig. 2: EF-1α (epimastigote). Heat map of EF-1α partners differentially expressed genes (DEGs) (epimastigote), representing total (Tot) and polysomal/granular (P/G) modulation across the time points of the growth curve. Grades of blue and yellow represent decrease or increase, respectively, of log2-ratio time points against day 1 of the growth curve.

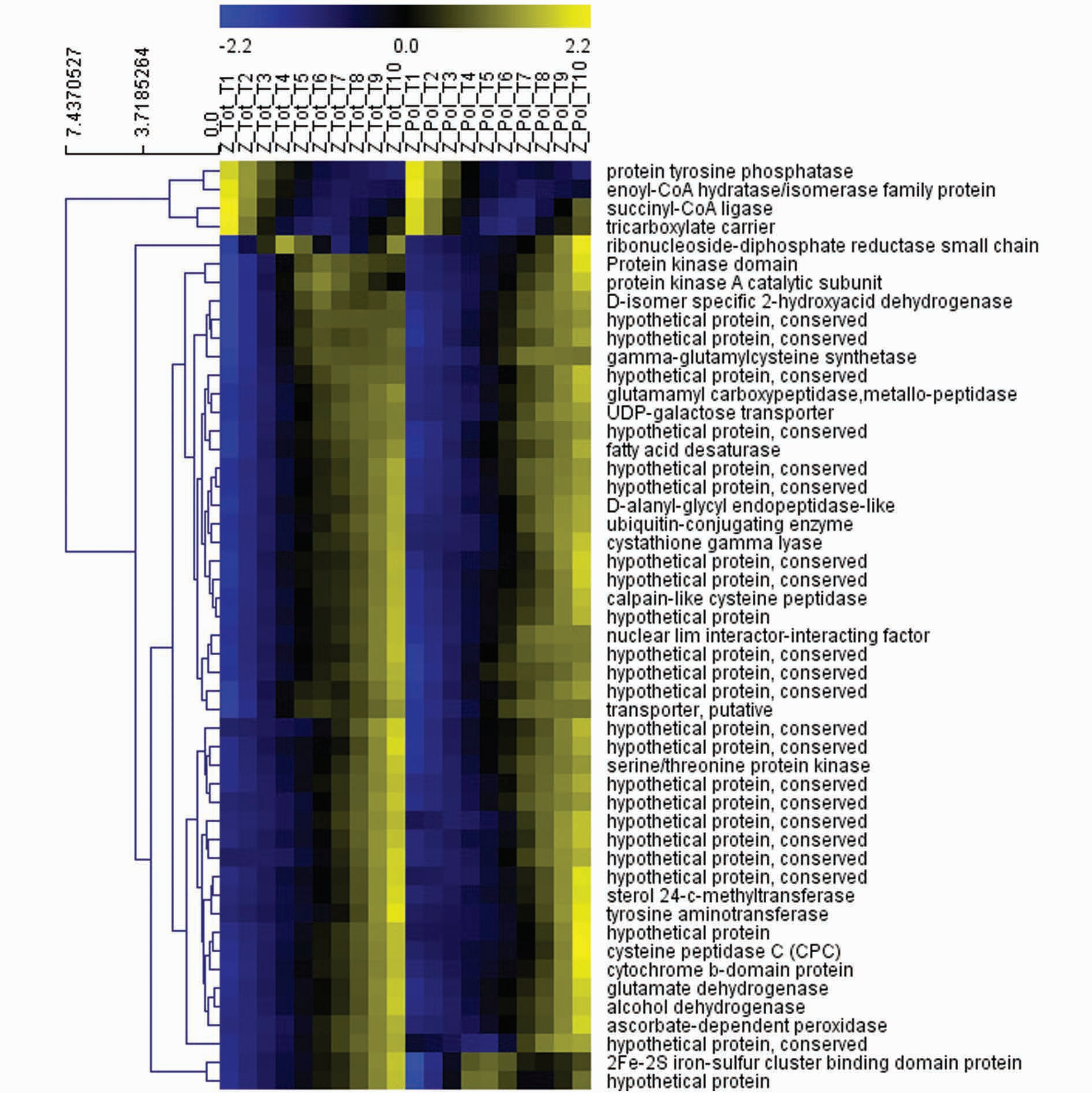

Fig. 3: EF-1α (epimastigote under nutritional stress). Heat map of EF-1α partners differentially expressed genes (DEGs) (epimastigote under nutritional stress), representing total (Tot) and polysomal/granular (P/G) modulation across the time points of the growth curve. Grades of blue and yellow represent decrease or increase, respectively, of log2-ratio time points against day 1 of the growth curve.

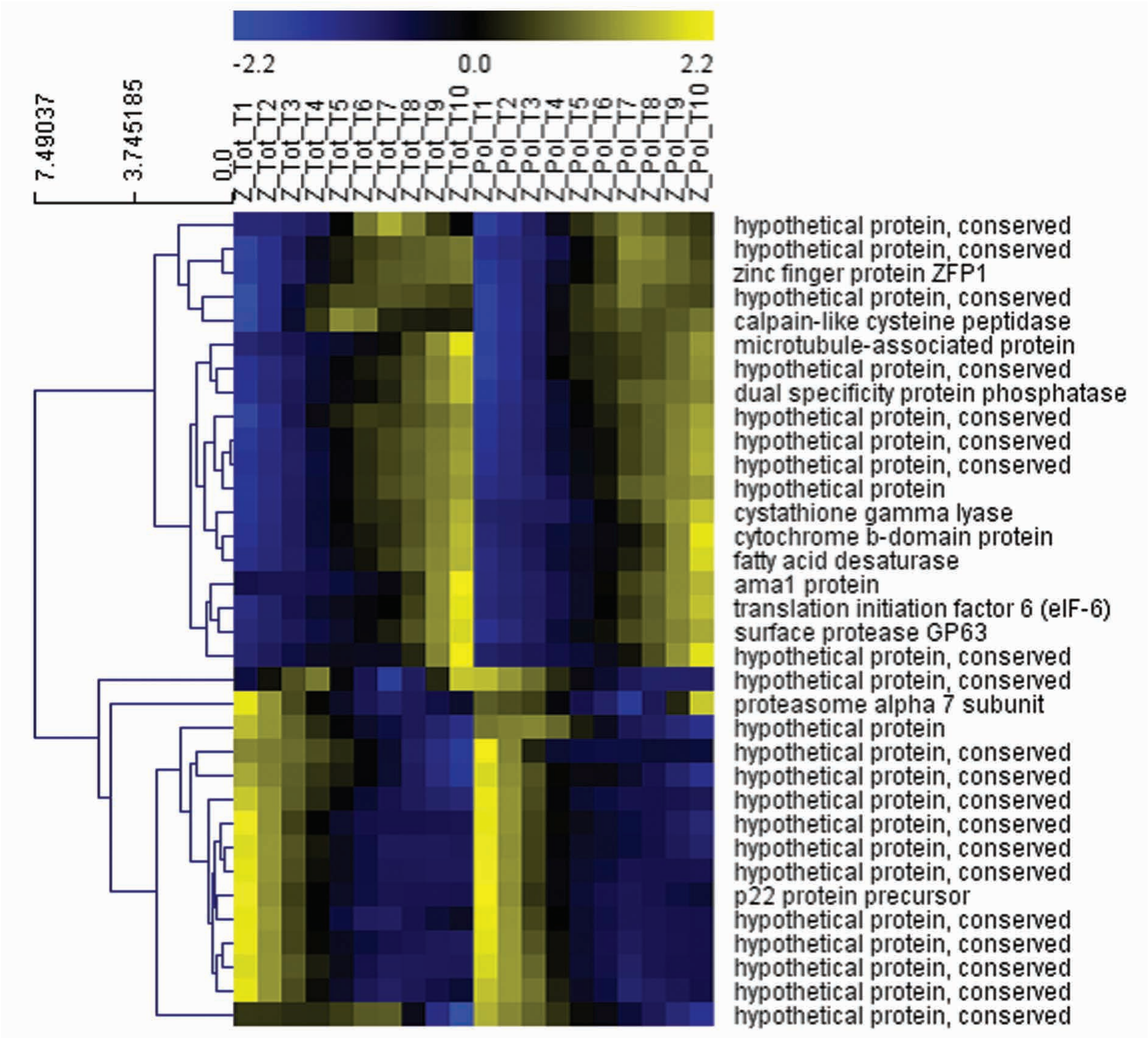

Fig. 4: NRBD1 (epimastigote). Heat map of NRBD1 partners differentially expressed genes (DEGs) (epimastigote), representing total (Tot) and polysomal/granular (P/G) modulation across the time points of the growth curve. Grades of blue and yellow represent decrease or increase, respectively, of log2-ratio time points against day 1 of the growth curve.

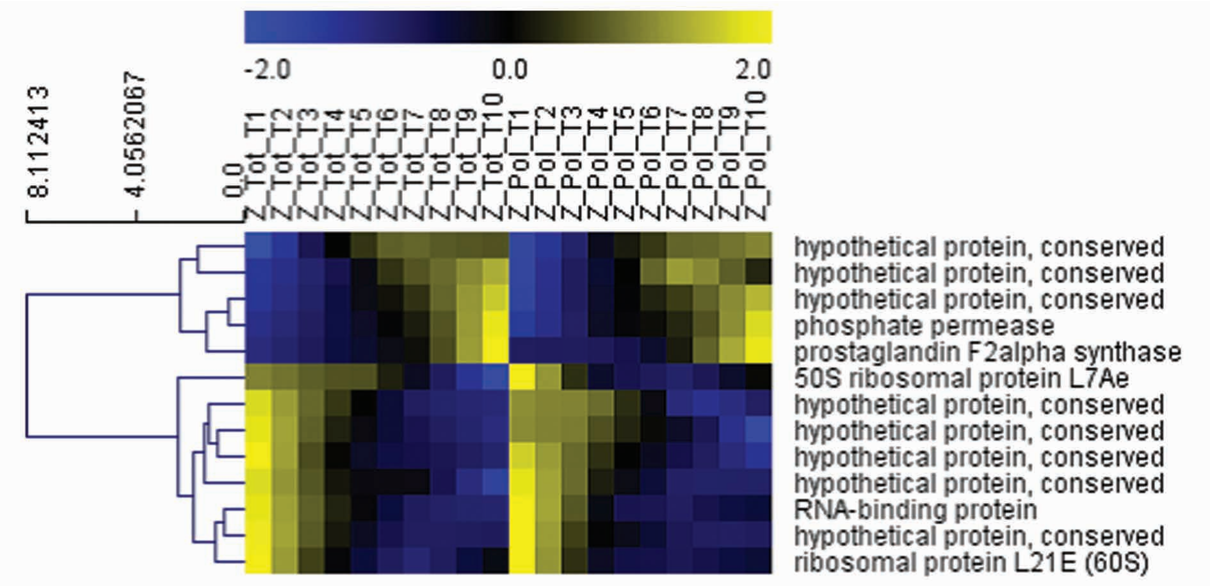

Fig. 5: NRBD1 (epimastigote under nutritional stress). Heat map of NRBD1 partners differentially expressed genes (DEGs) (epimastigote under nutritional stress), representing total (Tot) and polysomal/granular (P/G) modulation across the time points of the growth curve. Grades of blue and yellow represent decrease or increase, respectively, of log2-ratio time points against day 1 of the growth curve.

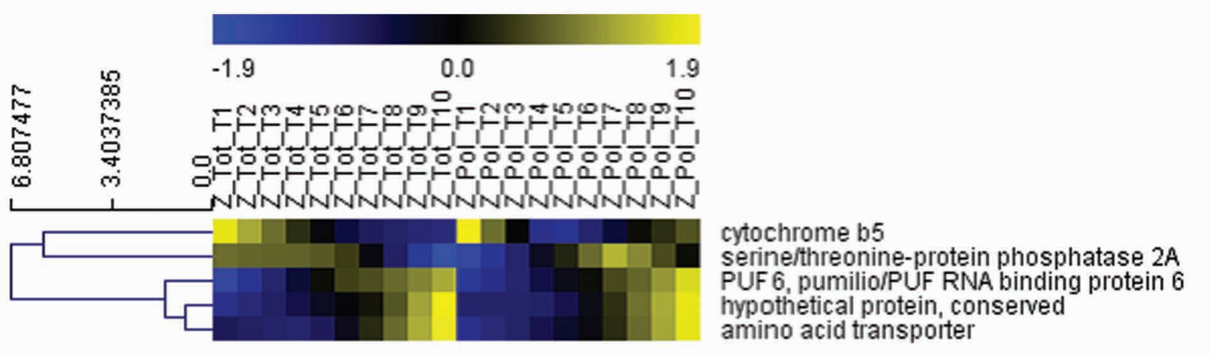

Fig. 6: PUF6 (epimastigote). Heat map of PUF6 partners partners differentially expressed genes (DEGs) (epimastigote), representing total (Tot) and polysomal/granular (P/G) modulation across the time points of the growth curve. Grades of blue and yellow represent decrease or increase, respectively, of log2-ratio time points against day 1 of the growth curve.

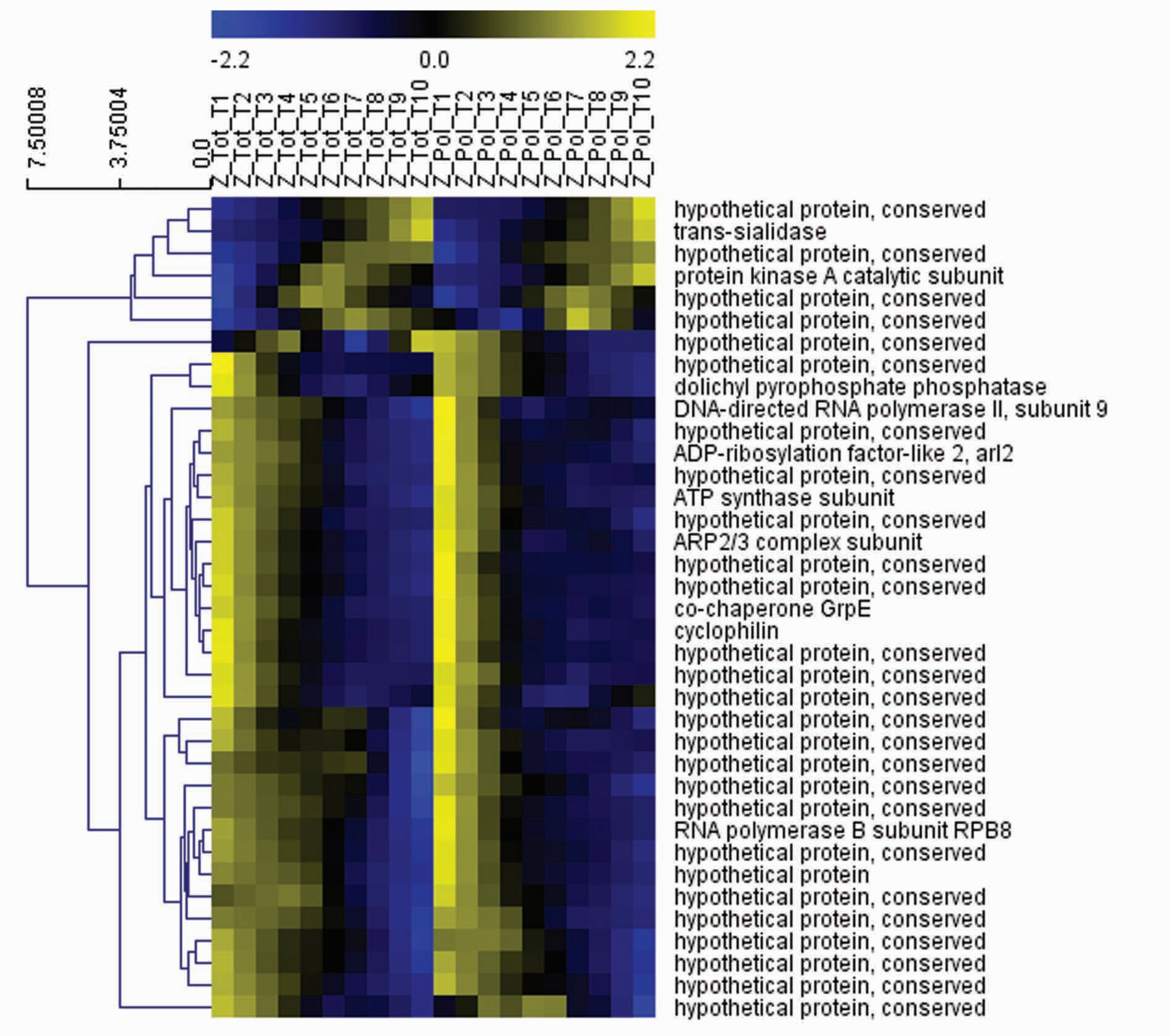

Fig. 7: RBP40 (epimastigote). Heat map of RBP40 partners differentially expressed genes (DEGs) (epimastigote), representing total (Tot) and polysomal/granular (P/G) modulation across the time points of the growth curve. Grades of blue and yellow represent decrease or increase, respectively, of log2-ratio time points against day 1 of the growth curve.

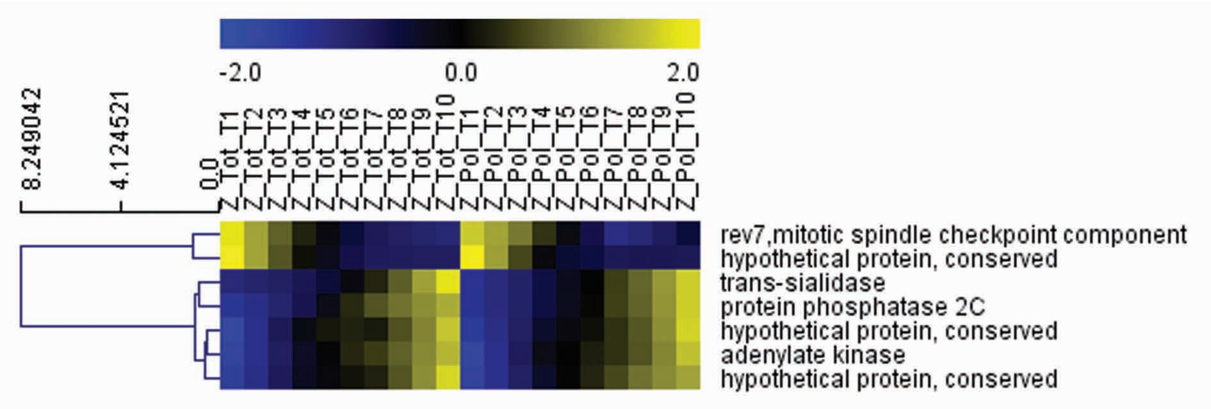

Fig. 8: ZC3H39 (epimastigote). Heat map of ZC3H39 partners differentially expressed genes (DEGs) (epimastigote), representing total (Tot) and polysomal/granular (P/G) modulation across the time points of the growth curve. Grades of blue and yellow represent decrease or increase, respectively, of log2-ratio time points against day 1 of the growth curve.

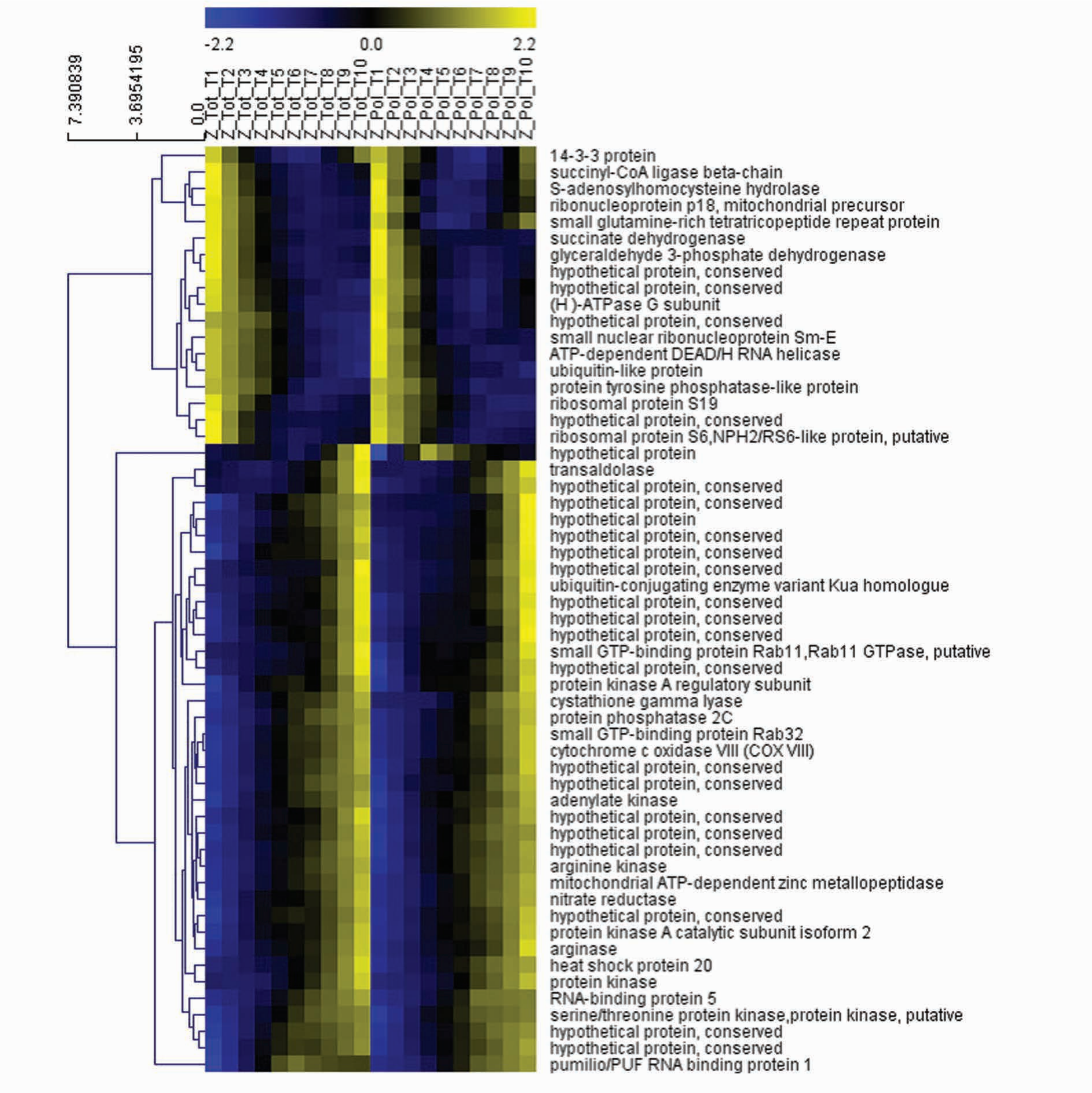

Fig. 9: ZC3H39 (epimastigote under nutritional stress). Heat map of ZC3H39 partners differentially expressed genes (DEGs) (epimastigote under nutritional stress), representing total (Tot) and polysomal/granular (P/G) modulation across the time points of the growth curve. Grades of blue and yellow represent decrease or increase, respectively, of log2-ratio time points against day 1 of the growth curve.

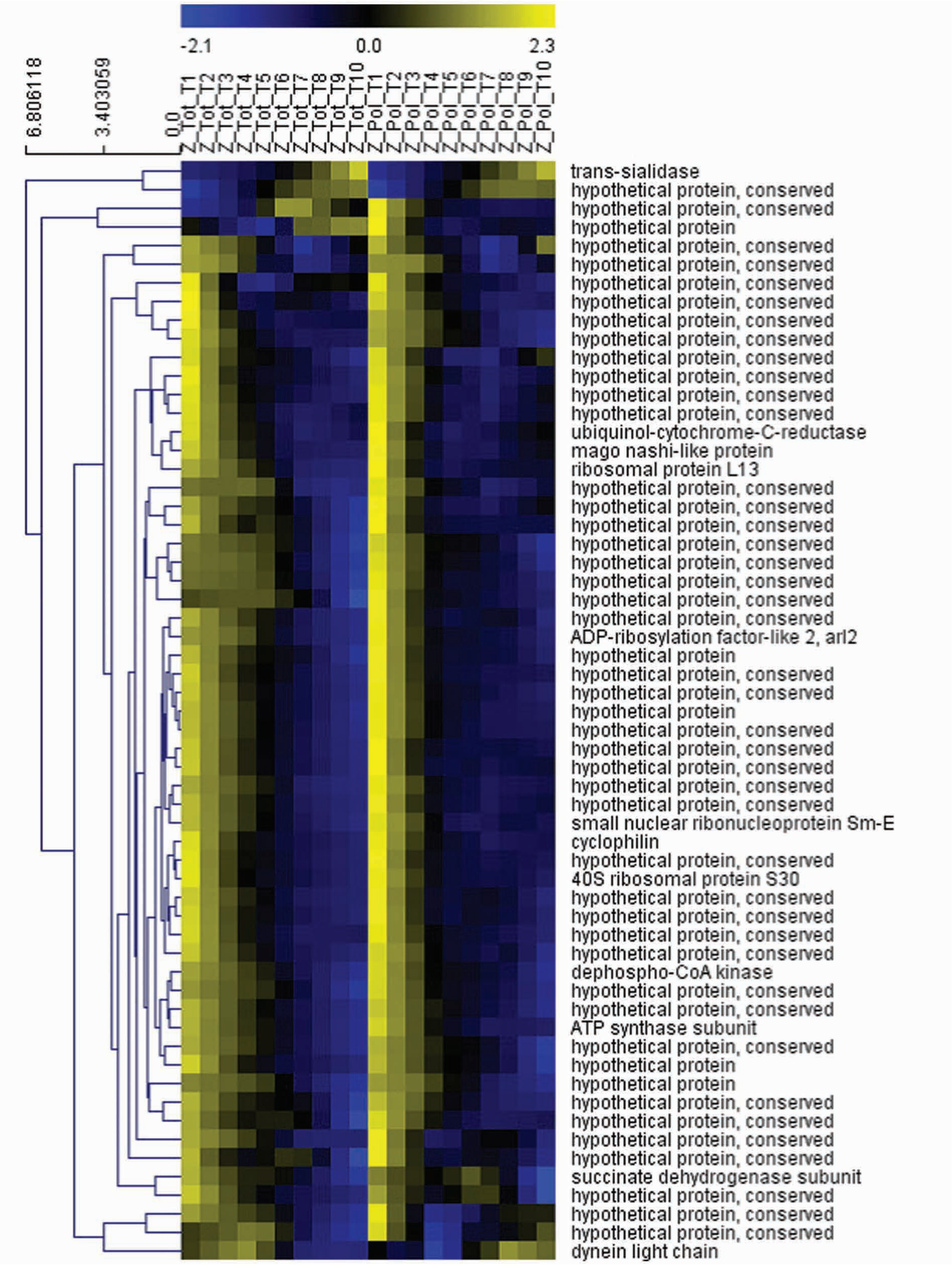

Fig. 10: ZFP2 (epimastigote). Heat map of ZFP2 partners differentially expressed genes (DEGs) (epimastigote), representing total (Tot) and polysomal/granular (P/G) modulation across the time points of the growth curve. Grades of blue and yellow represent decrease or increase, respectively, of log2-ratio time points against day 1 of the growth curve.

REFERENCES

Alves LR, Oliveira C, Goldenberg S. Eukaryotic translation elongation factor-1 alpha is associated with a specific subset of mRNAs in *Trypanosoma cruzi*. BMC Microbiol. 2015; 15: 104.

Alves LR, Oliveira C, Mörking PA, Kessler RL, Martins ST, Romagnoli BA, et al. The mRNAs associated to a zinc finger protein from *Trypanosoma cruzi* shift during stress conditions. RNA Biol. 2014; 11(7): 921-33.

Dallagiovanna B, Correa A, Probst CM, Holetz F, Smircich P, de Aguiar AM, et al. Functional genomic characterization of mRNAs associated with TcPUF6, a pumilio-like protein from *Trypanosoma cruzi*. J Biol Chem. 2008; 283(13): 8266-73.

Holetz FB, Alves LR, Probst CM, Dallagiovanna B, Marchini FK, Manque P, et al. Protein and mRNA content of TcDHH1-containing mRNPs in *Trypanosoma cruzi*. FEBS J. 2010; 277(16): 3415-26.

Mörking PA, Rampazzo RC, Walrad P, Probst CM, Soares MJ, Gradia DF, et al. The zinc finger protein *Tc*ZFP2 binds target mRNAs enriched during *Trypanosoma cruzi* metacyclogenesis. Mem Inst Oswaldo Cruz. 2012; 107(6): 790-9.

Oliveira C, Carvalho PC, Alves LR, Goldenberg S. The role of the *Trypanosoma cruzi* TcNRBD1 protein in translation. PLoS One. 2016; 11(10): e0164650.
